# Mapping of dynamic allostery within p38 alpha kinase via network analyses and NMR spectroscopy

**DOI:** 10.1038/s41467-026-76885-7

**Published:** 2026-08-22

**Authors:** Suchandra Roy Acharyya, Jörn Weisner, Rafael C. Bernardi, Rasmus Linser

**Affiliations:** 1 Department for Chemistry and Chemical Biology, TU Dortmund University, Otto-Hahn-Str. 4a, Dortmund, Germany; 2 Department of Physics, Auburn University, 380 Duncan Drive, Auburn, AL USA

**Keywords:** Molecular conformation, Computational biophysics, Solid-state NMR

## Abstract

Kinases are major drug targets especially in cancer therapy. However, the high degree of conservation of their active sites hinders the development of selective inhibitors, motivating a deeper understanding of kinase conformational ensembles and allosteric communication pathways. Here, we use dynamical network analysis to identify key residues involved in a dynamic allostery between the N- and C-lobes that connects the major functional units of the MAP kinase p38α. By combining NMR spectroscopy, activity assays, and in silico analysis of wildtype protein and mutants in the presence or absence of an active-site inhibitor, we experimentally validate the obtained architecture with respect to global protein motion and long-range allosteric modulation. Notably, the identified network highlights communication pathways across several functional sites, prominently involving the allosteric site, the activation loop, and even the lipid-binding domain with its embedded cryptic pocket in the C-lobe. These findings provide mechanistic insight into p38α allostery and suggest viable opportunities for the rational design of allosteric modulators of MAP kinases.

## Abstract (web summary)

Here, the authors apply dynamic network analysis, alongside a set of experimental validations, to probe correlated motions within p38α kinase. The data corroborate communication between its N- and C-lobes, including in particular the lipid pocket.

## Introduction

The family of p38 mitogen-activated protein kinases (MAPK) represents one of the principal elements within the MAPK signaling cascade, critically involved in mediating cellular responses to stress and inflammatory stimuli. A robust cluster of experimental evidence suggests that p38 can exert pro-oncogenic functions in various types of cancer^1^, which has made p38 one of the most important drug targets across a wide range of cancers. p38 kinases are prototypical kinases comprising a bi-lobal structural core that is divided into a smaller N-terminal and a larger C-terminal lobe. The catalytic active site lies between the two domains, forming a hinge bearing high flexibility, which is considered to be an important parameter for nucleotide exchange^2^.

Canonical activation of p38α MAP kinase requires dual phosphorylation of the activation loop, which promotes reorganization of the kinase domain towards catalytically competent conformations. However, activation is not adequately described as a simple binary transition between inactive and active structures. Enhanced-sampling simulations and NMR studies have shown that phosphorylation, nucleotide binding, and substrate/docking interactions progressively reshape the conformational free-energy landscape of p38α, shifting the ensemble toward active-like states without necessarily locking the kinase into a single active conformation^3^. This ensemble-based view highlights that the regulation of p38α activity involves several highly dynamic structural elements, including the catalytic loop, the N-terminal segment of the activation loop containing the conserved “DFG-motif”, the αC helix, and the hinge region^4^. Drug design efforts for this kinase have been focused predominantly on small-molecule inhibitors of the ATP binding site, prohibiting the activating phosphorylation of residues T180 and Y182. Addressing this critical functional element, however, a component widely conserved across the entire kinome, entails dose-limiting adverse effects owing to non-selective inhibition.

**Fig. 1:**
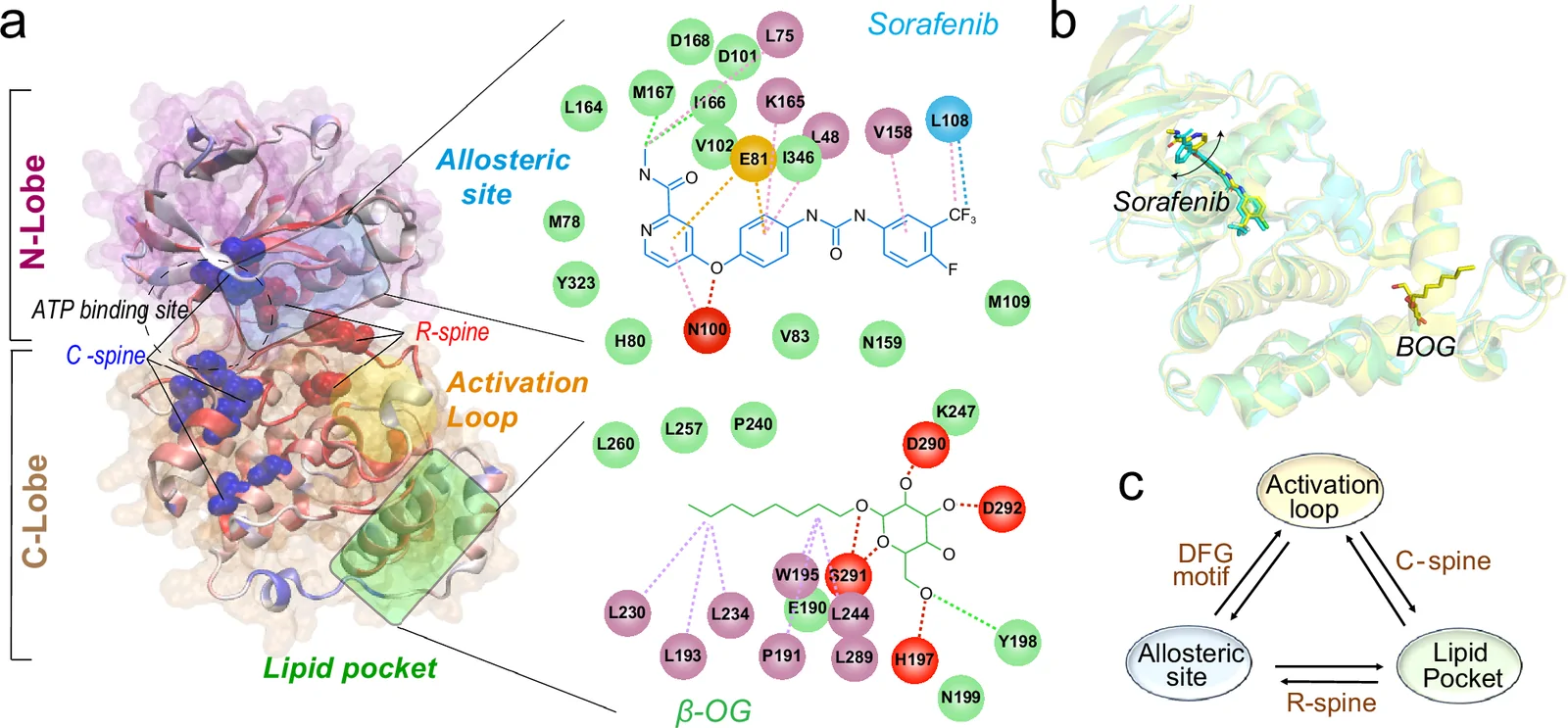
The interdomain communication to the lipid binding domain in p38α in question. **a** Structural representation of p38α kinase, highlighting the interactions of ligands binding to the allosteric pocket (exemplified for sorafenib) and the lipid pocket (shown for β-OG), modified from an output of Discovery studio^73^. Key structural features such as the activation loop, the catalytic (C-) spine, and the regulatory (R-) spine are highlighted. Interactions are color-coded as van-der-Waals (green), H-bonded (red), anion-pi (orange), alkyl (purple), and halogen interactions (blue). **b** Observed ring flip of the type-II inhibitor sorafenib between structures in the presence (semi-transparent yellow, PDB 3GCS) or absence (semi-transparent cyan, PDB 3HEG) of a lipid pocket binder (β-OG). **c** Sketch of the allosteric pathways between the active site, the adjacent, so-called allosteric pocket, and the lipid pocket in question.

Consequently, the identification of other potential locales that bind substrates, inhibitors, or allosteric effectors is of great interest^1^. In this context, compounds targeting TAB1-induced p38α autophosphorylation have recently been described. TAB1 acts as a non-canonical activator of p38α. These compounds, as well as inhibitors that selectively interfere with this pathway while only weakly affecting canonical MAPK activation, support the idea that p38α contains regulatory surfaces outside the ATP-binding site that can be exploited for pathway-selective modulation^5^. Conversely, modulators that bind in the “DFG-out” conformation, exploiting an additional site opening up close to the active site, represent the more common strategies^6^. Sorafenib (Nexavar®) is such a (“type II”) inhibitor, binding to this site but potently inhibiting nine other kinases^7^ (Fig. 1a). Its strategic use in combination with the drug SB202190 at the ATP-binding site, e.g., entails a synergistic effect that increases the apoptotic response in colorectal cancer cells^7^. However, the resemblance of this site to the conserved phosphate-binding site has resulted in little or no increase in the specificity of this combination regarding drug binding, toxicity, and dosage constraints. Much in contrast, a rather distant, cryptic pocket, the “lipid pocket”^8^ (Fig. 1a), is associated to the lipid-binding domain, hosting the “MAP kinase insert”^8^. Even though no physiological impact of its pharmacological addressing could be demonstrated as of yet, it is known to accommodate – in conjunction with a significant conformational change – a range of lipophilic molecules like *n*-octyl-β-glucopyranoside (β-OG) as well as suitable covalent binders addressing a conserved Cys closeby^9^. This hidden pocket in the C-terminal region of the protein has been shown to bear a primary sequence conserved over the p38 family and potentially allows for binding of more drug-like small molecules specifically generated in the quest for allosteric p38 inhibitors^10^.

Allosteric communication refers to an intramolecular mechanistic crosstalk between spatially distant sites that participates in modulating functionality as a function of external events^11^. Allostery, in a wider sense, is the event in which one site somehow “feels” changes in a different site^12,13^. As such, it can either be based on significant structural changes or rather – even in the complete absence of the latter – hinge on dynamic features that are mutually dependent across the protein^14^. This latter mechanism, commonly referred to as “dynamic allostery”^11^, involves the redistribution of correlated motions across the protein, which can be modulated or induced by regulatory events such as ligand binding, protein–protein interactions, or phosphorylation. In p38α complexes, evidence for long-range dynamic coupling is provided by both structural and dynamical observations: Structural superimposition of the “DFG-out” p38α complexes bound to sorafenib alone (PDB 3HEG^15^) and to both sorafenib and β-OG (PDB 3GCS^16^) reveals a flip of the pyridyl nitrogen and methyl substituent of sorafenib, despite a separation of more than 30 Å from β-OG bound in the lipid pocket (Fig. 1b). This slight but noteworthy difference in ground-state structures suggests that ligand binding at the distal lipid pocket alters the conformational ensemble of the kinase in solution. In addition, activation of p38α incurs dynamics differentially modulated across timescales, with activation-loop phosphorylation having been observed to quench ps–ns motions without altering the average conformation. Instead, uniform µs–ms backbone dynamics are induced by substrate binding, flattening the energy landscape and rendering key allosteric sites accessible^17^. A cross-lobe interdependency in the realm of dynamic allostery also seems to involve the lipid pocket, occupation of which was observed to lead to widespread chemical-shift perturbations and changes in dynamics^18^. Similarly, for p38γ kinase, another one of the four MAPK isoforms and a close relative to p38α with ~60 % sequence identity, motional changes in the kinase due to distant effectors have been assessed and observed from various angles^19–21^. These experimental insights for the two p38 kinases align with the identification of long-range community networks in protein kinase A (PKA), fundamentally orchestrating overall dynamics and hence functionality^22^, as well as findings in the Scr kinases^23^, epidermal growth factor receptor (EGFR)^24^, and Abl kinase^25^, where catalytic activity was seen to be modulated by very distant sites^21^. Corroborating Cooper and Dryden’s original theory, activation of PKA has recently been ascribed to a redistribution of fast and intermediate-timescale thermal fluctuations (the “violin model”), which explains the lack of apparent structural changes. Thereby, the formation of “hydrophobic spines”, specifically the C-spine assembled upon ATP binding, and the R-spine, linked to positioning of the αC-helix, an assembly regulated by upstream processes (also shown in Fig. 1a), is thought to aid in orchestrating suitable dynamic networks^26^. In MAP kinases, insights into such motional dependencies are of general interest from a biological perspective. However, they are of particular importance in the light of a motional connectivity between the different regulatory sites, the active site, or the lipid binding domain. A residue-specific mapping of such a dynamic network and identification of its key participants may aid understanding the molecular underpinnings of p38 regulation and even launch future pharmacological avenues based on the underlying pathways (Fig. 1c).

An in-depth interrogation of dynamic networks has become possible using molecular-dynamics simulations in combination with dynamic network analyses such as Dynetan^27^ or griNN^28^, where the correlations of motions assessed as generalized correlation coefficients based on mutual information are exploited to determine the strength of dynamic connectivity between different residues of the system^27^. The accuracy of dynamic network analysis is coupled to the limitations of MD simulations, also usually only interrogating one out of a plethora of different physiological states of a protein, and slightly variable results are usually obtained using different algorithms, node definitions, edge weighting, correlation metrics, and other details. Still, the approximate mapping of motional networks through dynamic network analysis can be assumed as an established and well-benchmarked procedure^29–31^. Whereas these in silico findings are rich in information, they do require verification by experimental means. Providing site-specific access to a large range of chemical and motional parameters, NMR spectroscopy is particularly well suited as a complement to such simulations^32^. While phosphorylation-dependent activation has been extensively characterized, the intrinsic allosteric communication architecture of the unphosphorylated apo kinase remains important for understanding the basal conformational ensemble from which activation and allosteric regulation emerge.

Here, we use state-of-the-art computational approaches based on a dynamical network analysis software (Dynetan)^27^ to characterize p38α dynamic networks from an NAMD^33^-based molecular-dynamics simulation. Perturbing those residues that turn out to be pivotal for the motional network through site-directed mutagenesis, we then use NMR in solution to monitor the consequences for the various mutants experimentally. Together, these data reveal an extended dynamic network spanning both kinase lobes and comprising several distinct allosteric “hotspots” in p38α. In addition to confirming the motional features deduced for other kinases, famously interrogated by slightly different in silico methods, such as for protein kinase A, or experimentally as for p38γ, as mentioned in more detail above, our analysis here not only integrates the in silico data with a large complementary set of observables, particularly from the NMR spectroscopy, but also identifies the MAP kinase-specific lipid-binding domain and its associated cryptic lipid pocket as integral components of the dynamic network, highlighting their potential relevance for allosteric therapeutic intervention.

## Results

Following the workflow summarized in Supplementary Fig. 1, and the system setup summarized in Supplementary Table 1, we performed molecular dynamics (MD) simulations of unphosphorylated forms of apo p38α, of sorafenib-bound p38α, and of p38α bound to both sorafenib and β-OG using NAMD 3^33^ with the CHARMM36 force field^34^. (See methodological details in the SI text. For the apo protein, Supplementary Figs. 2 and 3 show root-mean-square deviations as a function of time as well as root-mean-square fluctuations as a function of residue.) Note that the apo, unphosphorylated form of the kinase was specifically selected to understand the basal characteristics of the p38α kinase architecture and to warrant direct compatibility within the integrated approach involving both in silico analyses and extensive NMR studies. While phosphorylation and binding to interaction partners are known to further modulate the conformational ensemble and potentially reweight communication pathways, the qualitative existence or absence of long-range dynamic coupling between functional regions can be considered fundamental properties of the structure. Note that similarly, the formation of a disulfide bond, suggested to potentially form under certain physiological conditions in the “back” of the kinase^35^, might slightly impact the correlations obtained below but is not expected to have a fundamental bearing on the overall results of this study. For each system, five independent replica simulations of 1 µs each were carried out. For subsequent network and correlation analyses, the final 200 ns of each replica were concatenated and analyzed using Dynetan^27^ (described in more detail in the Methods and graphically summarized in Supplementary Fig. 4). This analysis included community detection and quantification of betweenness centrality, which identifies residues that are central to allosteric communication.

**Fig. 2:**
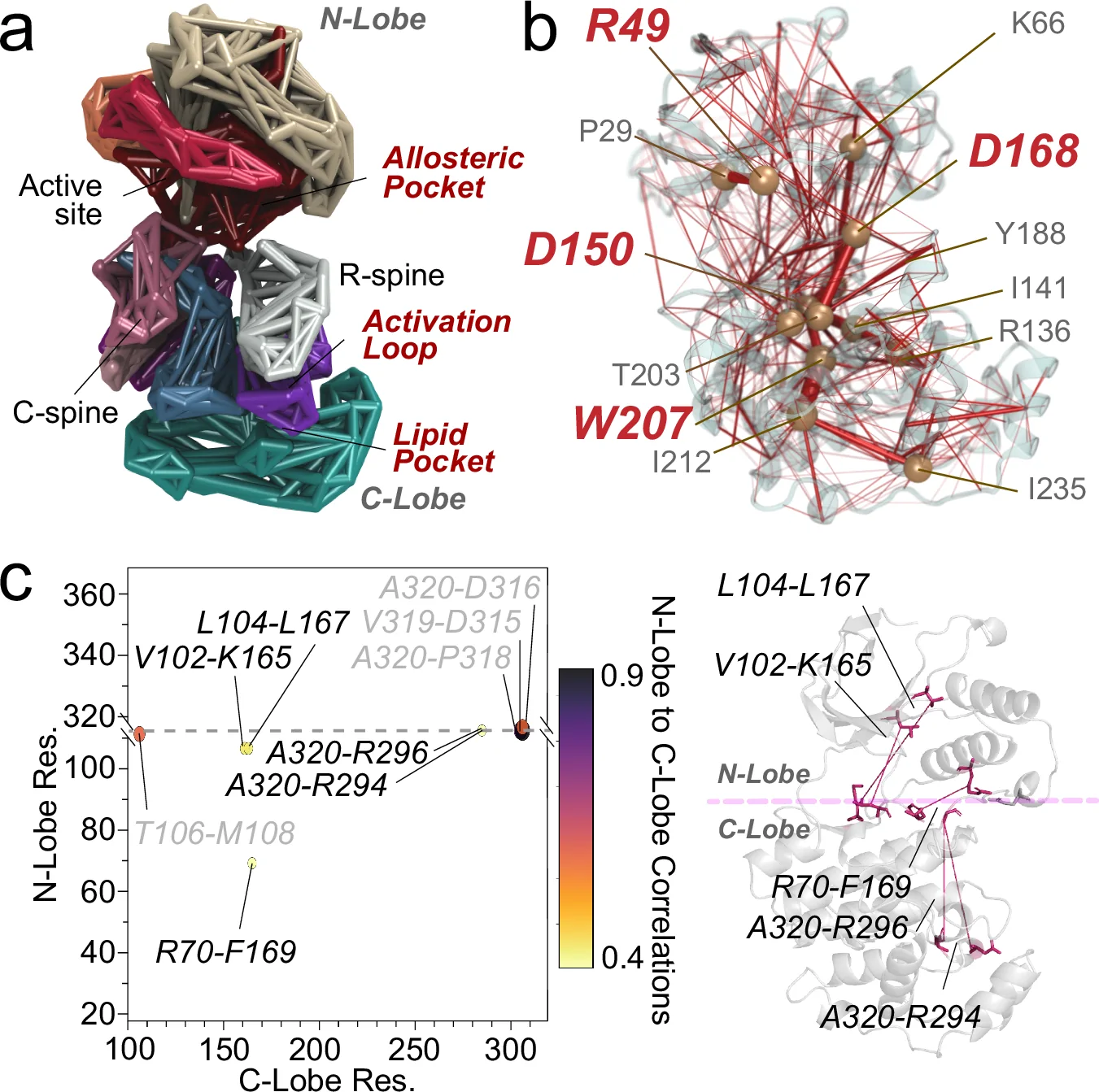
Dynamic network analysis of p38α kinase. **a** Clustering of the protein into 14 communities, largely reminiscent of the different structural elements of the kinase. **b** Residues with top betweenness centrality, i.e., the highest degree of information flow traversing them, also highlighting those network sites chosen for mutagenesis studies for network validation (red highlights). **c** Map of inter-residue dynamic-network correlations, specifically focusing on connections between N- and C-lobe residues. Pairs in black writing denote long-range correlations with a correlation coefficient above 0.3. Note that part of the C-terminus (320-360) lies in the N-Lobe, while the first transition from N- to C-lobe occurs at around residue 107. Correlations involving these covalent linkages are grayed out.

Community detection, the first output of network analyses, partitions the network into clusters that share common motion. This enables probing of consistency between the rich, atomic-resolution MD data here and coarser experimental studies on this or similar systems in the past. For the wild-type apo protein, this analysis yields 14 clusters, shown by different colors in Fig. 2a. Here, each community denotes a group of residues that exhibit strongly correlated motions with each other but weaker correlations with residues outside the group, reflecting coherent dynamical behavior within the protein. Despite the ~60% sequence identity between the isoforms only as well the stark differences in methodology, the clusters obtained from the in silico assessment here have a remarkable congruency to those (15) clusters found via purely experimental analyses (methyl scanning and chemical-shift perturbations) for p38γ^19^: Like in this previous study, our clusters divide the N-lobe into different active-site (magenta) and allosteric-pocket (brown) regions and take accountability of the R-spine (gray) and C-spine regions (pink). The C-lobe is divided into three clusters, where the MAP kinase insert (lipid binding domain) is the cluster shown in green in Fig. 2a and deviates from the cluster containing the activation loop (shown in purple). Notably, several of these dynamic features also align with community structures identified previously for a different kinase (protein kinase A, PKA) using Girvan–Newman-based network analysis^22^. Here, communities were segregated into 13 regions: In the N-lobe, the ATP binding pocket (magenta) is reminiscent of the PKA “Com A1”, the C-helix; the extended allosteric pocket of the p38α (brown) corresponds to “Com B”, and the R-spine of PKA (“Com C”) matches the gray cluster in p38α here^22^. In the C-lobe of PKA, “Com D” is a cluster congruent to the activation loop in p38α (purple), “Com E”, the C-spine cluster in PKA, matches the pink cluster in p38α, and “Com F” in the PKA case is the substrate binding region, which bears at least a slight similarity to the lipid pocket in p38α (green), which is part of the lipid binding domain only applicable for MAP kinases^22^.

Even more interestingly, particularly in light of this congruency with other kinases, we interrogated betweenness centrality, a property that identifies allosteric-network hotspots on the basis of their participation extent within different communication pathways. Betweenness is computed independently on the weighted network graph (and hence irrespective of the communities described for comparison with prior work above) and reflects generalized correlation-based edge weights obtained using the Floyd–Warshall algorithms^36^ (compare Methods section below). These results are shown in Fig. 2b and more specifically in Supplementary Table 2. Whereas naturally, a one-to-one comparison with other kinases is compromised due to the different primary structures and differences in the techniques applied, these pathways again seem qualitatively consistent with the experiment-based pathways connecting different regions of p38γ, where methyl mutations of several conserved sites were explored^19^. For example, the memory node L170 in p38γ, which takes additional input from the DFG-motif, appears as a motional hotspot in our study as well. Likewise, the hinge cluster of Abl kinase, constituting the D400 of the DFG-motif, had similar dominance in the long-range communication, suggesting the aspartate of the kinase to be a significant player in dynamic allostery^37^. In case of PKA, the F185 of the DFG motif coordinated together was found to be part of the allosteric communication instead of the vicinal aspartate, evocating the hypothesis whether the DFG motif itself is a necessary allosteric modulator^38^. D150, another node identified by our betweenness centrality study, belongs to the catalytic HRD-motif. In PKA, the homologous aspartate D166 was found to be the most crucial node that preorganizes the substrate’s phospho-acceptor site for efficient phosphotransfer^39^. (For Abl kinase and EGFR, the residues of this central catalytic machinery are the homologous D363 and D813, respectively^40–42^.) R49, another mediator that we identified as a residue with a high betweenness centrality, is a part of the extended allosteric pocket in p38α. While it reinstates its allosteric role in p38α in our study, a homologous residue in similar kinases that pinpoints its governance in their allosteric mechanism has not been found^43^. Most interestingly, however, W207, identified here as another key residue of the network, is positioned near the substrate binding groove. In PKA, the homologous W222 has been found to be an allosteric hub in the αF/FC region and a structural keystone anchoring the C-lobe^44,45^. (Abl and EGFR kinases have similar hydrophobes but there is no exact analog to pinpoint in this region^46^.) In p38α, this residue plays a particular role as it directly faces the lipid pocket, for which the possibility of designing allosteric effectors has been speculated^9^. Its high betweenness centrality hence directly rationalizes the participation of the lipid pocket as part of the dynamic network.

**Fig. 3:**
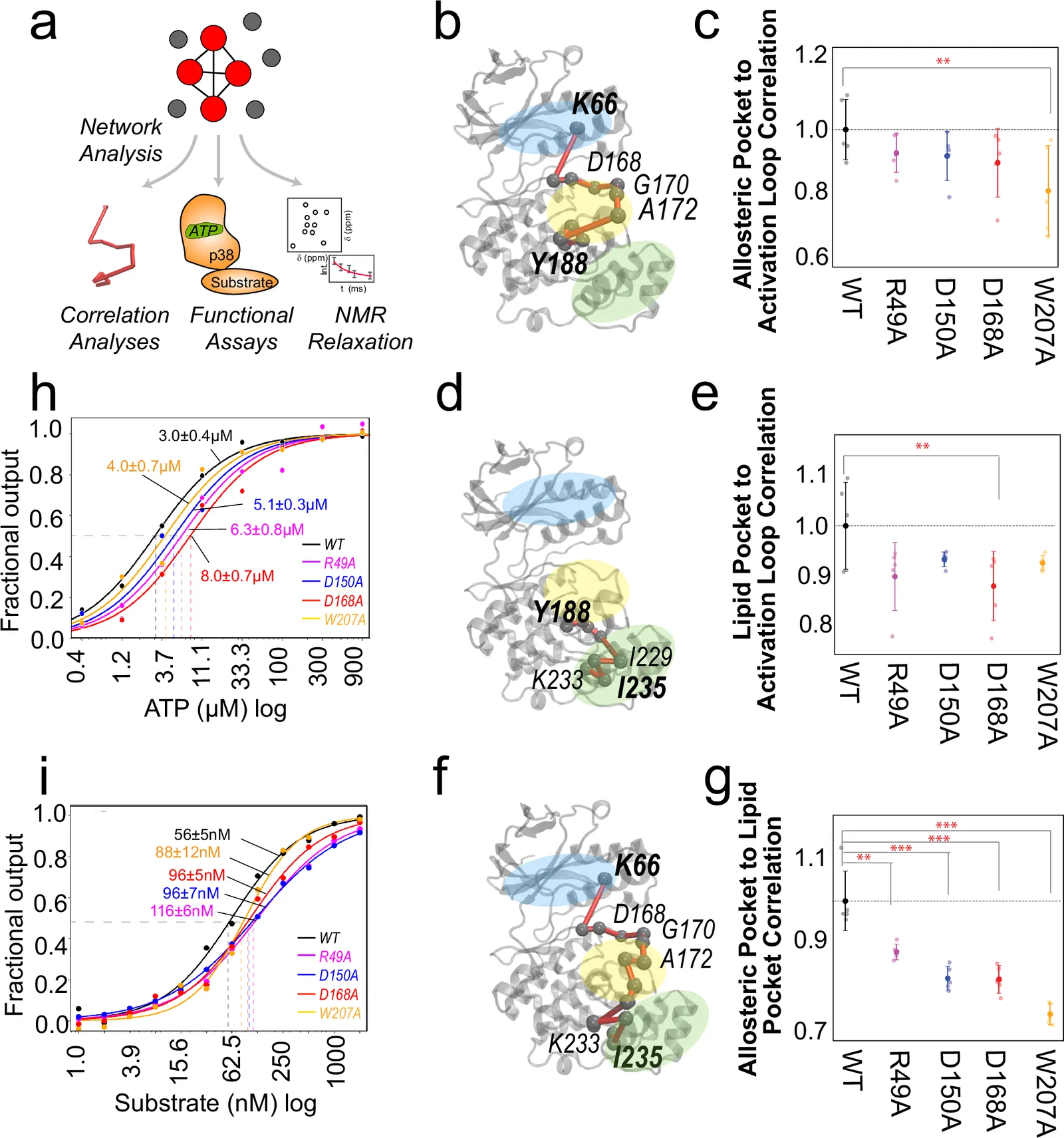
Comparison of shortest-path correlations and kinase activity upon dynamic-network mutation. **a** Strategy used to validate the dynamic-network analysis through in silico mutagenesis, functional assays, and NMR chemical-shift perturbation and relaxation measurements. **b** Residue-wise depiction of the shortest communication pathway from the allosteric pocket to the activation loop. **c** Total correlation of consecutive residue pairs along the allosteric-pocket-to-activation-loop pathway, normalized to WT, with WT set to 1. Exact two-sided permutation-test results were: R49A, *p* = 0.1862; D150A, *p* = 0.1664; D168A, *p* = 0.1414; and W207A, p = 0.0394. **d** Shortest communication pathway from the lipid pocket to the activation loop, with key residues highlighted. **e** Total correlation along the lipid-pocket-to-activation-loop pathway, normalized to WT. Exact *p* values were: R49A, *p* = 0.0701; D150A, *p* = 0.1582; D168A, *p* = 0.0425; and W207A, p = 0.1273. **f** Residue-wise depiction of the shortest communication pathway from the allosteric pocket to the lipid pocket. **g** Total correlation along the allosteric-pocket-to-lipid-pocket pathway, normalized to WT. Exact *p* values were: R49A, *p* = 0.0171; D150A, *p* = 0.0026; D168A, p = 0.0022; and W207A, *p* = 0.0005. In (**c**,**e**,**g**), data are presented as mean ± sample SD from *n* = 5 independently initiated 1 µs MD trajectories per system. The final 200 ns of each trajectory were analyzed. WT was used as the control, and each mutant was compared separately with WT using a two-sided non-parametric permutation test with 10,000 label permutations. The observed difference in mean normalized correlation was used as the test statistic. No adjustment for multiple comparisons was applied, and the reported *p* values are unadjusted. Hedges’ *g* effect sizes and 95 % bootstrap confidence intervals for the mean differences are provided in Supplementary Table 6. Degrees of freedom are not applicable to this permutation-based test. Statistical annotations indicate **p < 0.05 and ***p < 0.01. **h** HTRF-based ATP titration comparing ATP-dependent activity of WT and mutant p38α constructs over an ATP concentration range of 0–1000 µM. The fluorescence ratio *f*_*665*_*/f*_*620*_, measured in the initial-rate regime, was fitted using the Michaelis–Menten equation, and best-fit *K*_M_ values are shown. **i** HTRF substrate titration using increasing concentrations of GST-tagged activating transcription factor 2 substrate. Responses were fitted using the Hill equation, and fitted EC_50_ values are shown. One activated protein preparation was used per construct, and each concentration was measured in four technical replicate wells. Plotted points represent the arithmetic mean of the four wells; variability between technical replicates is not shown. Curves were normalized to the fitted *ν*_*max*_ of the corresponding protein variant to compare fractional saturation independently of absolute signal amplitude. The raw data are shown in Supplementary Fig. 16. Source data are provided as a Source Data file.

Beyond the overall inter-lobe correlations (shown in Fig. 2c and Supplementary Table 3), which confirm both short-range connectivities dictated by the three-dimensional fold of the protein (e.g., R70/F169) and longer-range cross-talk such as between residues L104 and L167 or N102 and K165, we specifically examined the communication pathways linking the allosteric pocket, the activation loop, and the lipid pocket. For this reason, we picked one representative residue of the network for each of these sites/clusters and computed the cumulative pairwise correlation (the sum of degrees of correlation between any two neighbors) within the shortest path of communication. Note that the shortest path only represents one of a multitude of possible routes with decreasing importance but allows for a direct comparison of any changes/rerouting/abrogation of communication between different mutants. For the allosteric site, represented by residue K66, towards the activation loop (Y188), the information flux traverses the D168 and G170 of the DFG motif, similar to p38γ (Fig. 3b compare Supplementary Table 5)^19–21^. Accordingly, the shortest path between the activation loop and the lipid pocket, with residues such as K233 and I235, is shown in Fig. 3d. Pathways between the two pockets (between K66 in the allosteric pocket and K233 and I235 in the lipid pocket) were also calculated (without specific consideration of the activation loop). This path involves the DFG motif, but bypasses the activation loop to channelize the communication to the lipid-binding domain (Fig. 3f). Importantly, all of the above analyses identify a network of allosteric communication that interconnects not only the N- and the C-lobe generally but also involves the lipid-binding domain and specifically the lipid pocket. These in silico results rationalize the existence of functional communication pathways between the active site and the lipid-binding domain, supporting potential avenues for targeting MAP kinases through allosteric modulation.

To assess whether those networks are inherently apparent already from crystallography, the nodes identified were further examined in terms of B-factors derived from the apo/unphosphorylated crystal structures^34^. Supplementary Fig. 5 shows a depiction of B-factors. However, the B-factors of the hotspots of the dynamic network are mostly in the intermediate range (see a list of B-factors for all major nodes of the network in Supplementary Table 4). Even though the absolute values of B-factors can vary between conditions, these residues do not seem to be particularly disordered within their respective crystals. (For example, residues R49, D150, and D168 have relatively low values of 0.509, 0.342, and 0.497, respectively. W207 shows an even lower value of 0.056, making it a very well-defined residue.) Even though a flexible character/flexibility of specific residues at physiological temperatures can sometimes be forecasted from the cryogenic crystallographic viewpoint, it seems that the allosteric hotspots here are not recognizable through a specific degree of disorder. For further elucidation of changes to the networks, we hence turned to room temperature dynamics data in solution, assessed by a collection of NMR parameters (see below).

Overall, to validate the existence and characteristics of the dynamic allosteric network identified by dynamical network analysis, we pursued a two-level approach involving additional in silico analyses and experimental validation. In both cases, the network was probed by “mutating” residues identified as key nodes based on high betweenness centrality. For this downstream analysis, in particular correlation analyses, ligand (sorafenib) binding studies, solution NMR chemical-shift perturbations and NMR relaxation, as well as biochemical assays (see below), four of the nodes (R49, D150, D168, and W207) returned by the network analysis/betweenness centrality as central hubs were selected and (one at a time) replaced by alanine. Note that the number four is an arbitrary choice to broadly validate the presence of the network, rather than specifically verifying individual nodes. MD data (five 1 µs trajectories for each of the four mutants) were obtained using similar strategies as for the wild type (Supplementary Fig. 6) and re-analyzed with the mentioned network framework. These analyses revealed differences in both betweenness-derived pathways and altered community structure compared to the wild type (Supplementary Fig. 7). The effects of mutation on long-range communication were also quantified by evaluating inter-lobe (N–C) correlations and correlations along the wild-type-defined shortest communication paths (Supplementary Tables 2, 3, 5). Total correlation maps and N-C lobe correlation maps for all ensembles are provided in Supplementary Figs. 8 and 9, respectively. Whereas the tertiary structure and hence many correlations are necessarily maintained with respect to Fig. 2c, changes with respect to long-range correlations can be witnessed in each of the cases. (Difference maps (mutant—wild type) are shown in Supplementary Fig. 10.) To focus on pathway-level effects, we again selected one central residue per structural element (K66 for the allosteric pocket, Y188 for the activation loop, and I235 for the lipid pocket as before) and computed the shortest paths between them also for the mutants. The wild-type path served as the reference, and the cumulative amounts of pairwise correlations between any two consecutive residues along this path were evaluated for the wild type and each mutant. Uncertainty was estimated from five replicas, each partitioned into five windows (Supplementary Table 6). Across all paths (allosteric site to activation loop, Fig. 3b; lipid pocket to activation loop, Fig. 3d; and allosteric site to lipid pocket paths, Fig. 3f), mutant correlations were consistently lower than in the wild type protein. Interestingly, W207A, despite its residence in the C-lobe, has a very strong effect on the allosteric-site-to-activation-loop path ( ~ 20%, Fig. 3c). Conversely, for the lipid-pocket-to-activation-loop correlation, D168A, the DFG-mutant lying in the hinge of the N-lobe, has a strong impact. ( ~ 10%, Fig. 3e); both residues lie distal to the respective pathways, consistent with a dense allosteric network. Intriguingly, with respect to the direct pathway between the allosteric pocket and the lipid pocket, all the nodes identified by the network mutated out (R49A with ~10%, D150A, D168A, both with ~20%, and W207A, ~25%) show a substantial, statistically significant drop (Fig. 3g). Note that the wild-type optimal pathways were highly reproducible across the five independent replicas for each system, supporting the routes as robust features of the wild-type network rather than replica-specific (random) artefacts. By contrast, the consistent depreciation of these dominant communication pathways here reveals a redistribution of correlated motions between the two pockets upon mutation.

In addition to the apo proteins, we also examined how the presence of an active/allosteric-site binder would influence the network. For this purpose, we employed the in silico interrogation of wild type and mutants outlined above, however, starting from the sorafenib-bound X-ray structure 3HEG. Supplementary Fig. 11 and Supplementary Table 7 show the embedding of this ligand as interrogated using Discovery Studio^47^ and its docking scores, respectively. MD simulations again afforded trajectories of 5 replicas each (Supplementary Fig. 12). At first, we assessed motional correlations between the protein and the drug molecules. Supplementary Fig. 13 shows these correlations, where for simplicity, the ligand sites were coarse-grained into a Western and an Eastern part analyzed individually. Interestingly, we observed that the total amount of cumulated correlation drops slightly upon mutation, most prominently for mutant R49A (Supplementary Fig. 13). More importantly, however, we then pursued the above examination of interdomain communication within the ligand-bound protein. For the wild-type complex, shortest communication paths connecting the allosteric pocket, activation loop, and lipid pocket were identified using the same representative residues as in the apo analyses. Structural representations (Supplementary Fig. 14A, C, E) reveal that inhibitor binding now reroutes the preferred communication pathways linking the allosteric pocket, the activation loop, and the lipid pocket with each other. For each mutant complex, we quantified the cumulative pairwise correlations along these paths, using the ligand-bound WT as the reference. Now, taking the new, ligand-bound routes of the wild type protein as the reference and mutating the above-mentioned network hotspots, no significant reduction in correlation is observed anymore (Supplementary Fig. 14B, D, F). This indicates that ligand binding causes the motions of the protein to alter and largely suppresses its intrinsic dynamic network. Consistently, no systematic gains or losses in cumulative correlations are detected along the ligand-bound paths.

Together, these results demonstrate that (i) mutations at network sites attenuate communication in a pathway- and residue-dependent manner, (ii) sorafenib reshapes the communication routes relative to the apo protein, and (iii) ligand binding to the active site globally quenches the dynamic network propagation. Assessing a single shortest communication path neglects the fact that allosteric signaling typically arises from the collective contribution of multiple pathways. Moreover, the in silico identification of long-range communication paths spanning many network edges is intrinsically less sensitive than the analysis of shorter network fragments, such as those shown in Fig. 2b. Even with the extensive sampling afforded by five independent microsecond-long MD replicas, these pathway-level correlations retain a certain statistical error, underscoring the need for experimental validation based on vastly larger ensembles of molecules. To experimentally (i.e., with a much bigger ensemble) probe the functional consequences of hijacking the dynamic network, we therefore first assessed the enzymatic integrity of the individual mutants relative to the wild-type protein using Homogeneous Time-Resolved Fluorescence (HTRF) assays^48^ (Fig. 3h i, see details in the Methods).

HTRF is a robust, high-throughput, low-background FRET-based technique that combines the sensitivity of time-resolved fluorescence with a homogeneous assay format. In this study, HTRF was employed as a proxy for protein substrate binding efficacy (using GST-tagged activating transcription factor 2 as a substrate) in the realm of its overall biochemical activity as well as to probe the integrity of binding of ATP between wild-type and mutant constructs^48^. Adding to the wild-type protein, whose expression and purification have been described in the literature^49^, all of the above-mentioned mutants were generated via site-directed mutagenesis, and the protein was expressed recombinantly in *E. coli* and purified according to the existing protocols (see the Methods and Supplementary Fig. 15, for biochemistry details). The dependence of reaction speed on either ATP or substrate concentration serves as a proxy for binding of either interaction partner (see details in the Methods). Kinetic characterization was pursued via Michaelis-Menten fit for ATP^50^ as a standard saturation model for enzyme kinetic data and via Hill fits for the substrate, to allow an additional slope parameter and capture possible sigmoidal behavior^51^. Figure 3h i, as well as Supplementary Fig. 16, confirm the highest binding competencies, with the lowest Michaelis-Menten constants (*K*_M_) and EC_50_, for the wild-type enzyme (Supplementary Table 8). The raw fluorescence signals are shown in the Supplementary Information (Supplementary Fig. 16). To compare relative activity differences, the signal output was normalized to the fitted *v*_*max*_. The normalized ATP- and substrate-dependent responses were then fitted to assess mutation-induced shifts in the apparent concentration required for response. Impaired binding competencies occur in all the mutants, with higher ATP concentrations required to reach a similar fractional saturation as in the wild type. This, even though less pronounced, also applies to substrate binding. W207A appears to be comparatively less affected in terms of apparent ATP affinity and substrate competitiveness. In contrast, D168A shows the strongest reduction in ATP-dependent activity (Fig. 3h, Supplementary Fig. 16A), whereas R49A shows the greatest deviation in substrate competitiveness (Fig. 3i, Supplementary Fig. 16B). It has to be taken into note that neither of these sites are part of the actual enzymatic process, consistent with the possibility of disrupted enzymatic potency due to allosteric modulation from distant sites of mutation. Changes in *K*_M_/*K*_*0.5*_ and hence the affinities to ATP/substrate are returned by the assay in a more robust fashion than the *v*_max_ values (grayed out but still displayed in Supplementary Table 8), as consistent protein concentrations and degree of phosphorylation between samples cannot be guaranteed. It is, however, likely that both of these features are impinged on by changes in dynamic-network integrity upon mutation. Note that, overall, the impact is expected to be minor and the insights from these experiments are limited. They should hence be understood as a mere motivation to follow up with detailed molecular-level assessments, as shown in the following.

If protein motions were purely local and independent, a point mutation would be expected to affect the substituted residue and its immediate environment. By contrast, NMR studies have shown that perturbations within dynamic allosteric networks can induce long-range changes in both site-specific chemical shifts and relaxation properties across multiple timescales^19,52^. To assess such putative long-range changes in protein dynamics, we used solution-state NMR on ^13^C/^15^N/^2^H-labeled, unphosphorylated, apo protein samples expressed in triple-labeled media and purified using successive rounds of Ni-affinity, anion exchange, and size exclusion chromatography according to published procedures^49^. In spite of multiple attempts and in contrast to all other proteins mentioned here, the plasmid of mutant W207A could not be successfully expressed as a stable triple-labeled construct in deuterated minimal media, precluding its structural and dynamic interrogation by NMR. Note that the unavailability of the W207A mutant for the below NMR studies is unfortunate, but even without it, the overall existence of the network can be validated by the three successful network mutations. Peak assignments were achieved via 3D HNCA spectra for both wild-type and mutant constructs, complemented by TROSY-HSQC used as fingerprint spectra (Supplementary Fig. 17), upon which existing assignments (BMRB entry: 17471) could be transferred in a stepwise manner. Independent of the network mutations, we also introduced V38A; in this case, however, specifically as an experimental negative control. V38 lies in a folded element of the tertiary structure, but outside the predicted dynamic network, allowing us to compare any “regular” alterations induced locally to global changes entailed by a tight dynamic network.

**Fig. 4:**
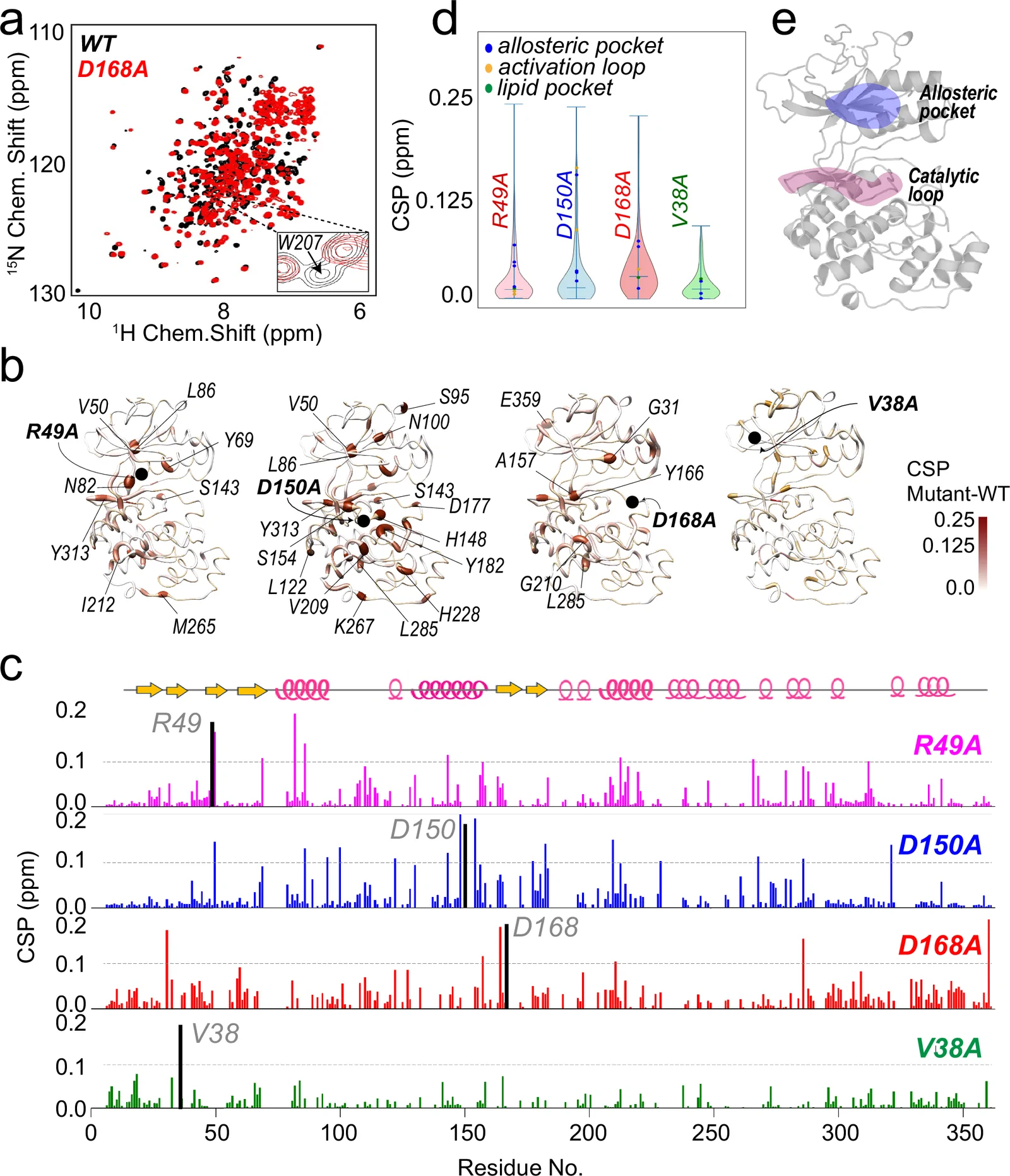
Chemical-shift perturbations (CSPs) induced upon mutation of key dynamic-network residues. **a** Overlay of [^15^N, ^1^H] TROSY-HSQC spectra for wild-type and mutant D168A. The mutation results in substantial spectral reorganization, including disappearance of the W207 resonance in the lipid pocket. (Note that, in contrast to the 2D spectrum shown here, the actual CSPs were read out from 3D spectra.) Spectra for the other mutants are shown in Supplementary Fig. 18. **b** CSP values for the different mutants relative to WT, mapped onto the protein structure. Perturbations extend beyond the local environment of the mutation, indicating widespread conformational changes throughout the protein. **c** Residue-specific CSP values plotted as a function of sequence. Dynamic-network mutants show elevated perturbations at several structurally distant positions, whereas the control mutant V38A shows limited CSPs outside its immediate vicinity. The uncertainty associated with the CSP values, estimated from the linewidths of the ^1^H, ^15^N, and ^13^C_α_ resonances, was approximately 0.069 ppm. **d** Violin plots showing the distributions of residue-specific CSP values for R49A, D150A, D168A, and V38A relative to WT. The numbers of residues included were *n* = 210 for R49A, *n* = 198 for D150A, *n* = 185 for D168A, and *n* = 220 for V38A. Violin widths represent the kernel-density distributions of the CSP values, the central horizontal marker indicates the median, and the capped vertical lines span the observed minimum and maximum values. Each residue represents one observation within the corresponding NMR dataset and not an independent biological replicate. Data were obtained from one independently expressed and purified protein sample per construct and are presented descriptively. Dots corresponding to residues from the allosteric pocket, activation loop, and lipid pocket are identified by the key within the figure. **e** Main affected regions, defined by the consistent occurrence of strong CSP values greater than 0.12 ppm in at least two of the mutants R49A, D150A, and D168A. Source data are provided as a Source Data file.

We first employed chemical-shift perturbation (CSP) analysis to probe changes in the average chemical environment of backbone amides across the protein. In the absence of allosteric communication, the chemical shifts of very distant residues would not be expected to change upon slight structural changes around the mutation site. Instead, however, the analysis revealed widespread deviations across the backbone for all functional mutants involved in the allosteric coupling. Notably, D168A, the “DFG-loop mutant”, exhibited the most dramatic global extent of CSPs across the structure (Fig. 4), including long-range effects such as the complete disappearance of the W207 peak in the C-lobe (Fig. 4a). As mentioned above, apart from its identification as a spatially distant key residue involved in the interdomain communication, W207 also represents a marker for the lipid pocket. The widespread CSPs hence not only corroborate the sensitive influence of D168A on allosteric interactions and domain coupling, but indirectly also the participation of W207 in the allosteric network. The distributed nature of dynamic perturbations throughout the protein for the network mutants can be recognized from Fig. 4b, depicting chemical-shift perturbations for the different variants compared to the wild type. The situation for the mutants R49A and D150A is comparable, with perturbations being slightly lower than in D168A overall. (Spectral overlays for the other mutants are shown in Supplementary Fig. 18.) In contrast to the mutants focused on hotspots found for the dynamic network, the negative control V38A showed indeed very limited chemical-shift changes (last mutant in Fig. 4b–d and Supplementary Fig. 18). The stark differences in chemical-shift perturbations between mutations within the dynamic network and the negative control is also seen from the violin plots shown in Fig. 4d, supporting V38A’s role as a mutation benign to the conformational ensemble and underscoring the global conformational remodeling induced by the actual network-disruptive mutations on the contrary. Consolidated patterns of common CSPs across the network mutants, and numerical changes pertaining to specific mutations are deciphered in Fig. 4e and Supplementary Table 9, respectively. In general, the N-lobe to C-lobe connection, the substrate-binding groove in the C-lobe, and the N-lobe regulatory region, comprising the P-loop elements and the allosteric pocket, seem to be consistently perturbed. The trends therefore indicate both mutation-specific and shared effects: individual mutants perturb distinct local residues, but the recurrence of CSP changes in common regions supports a broader, network-level impact rather than isolated local structural changes. These findings provide experimental support for mutations within hotspots identified by dynamical network analysis to indeed induce substantial allosteric rewiring of the kinase conformational ensemble. Note that also the residues shown to form a disulfide bridge under certain cellular conditions (see above)^35^ appear in two mutants, which may suggest the possibility of network modulation by oxidation state.

**Fig. 5:**
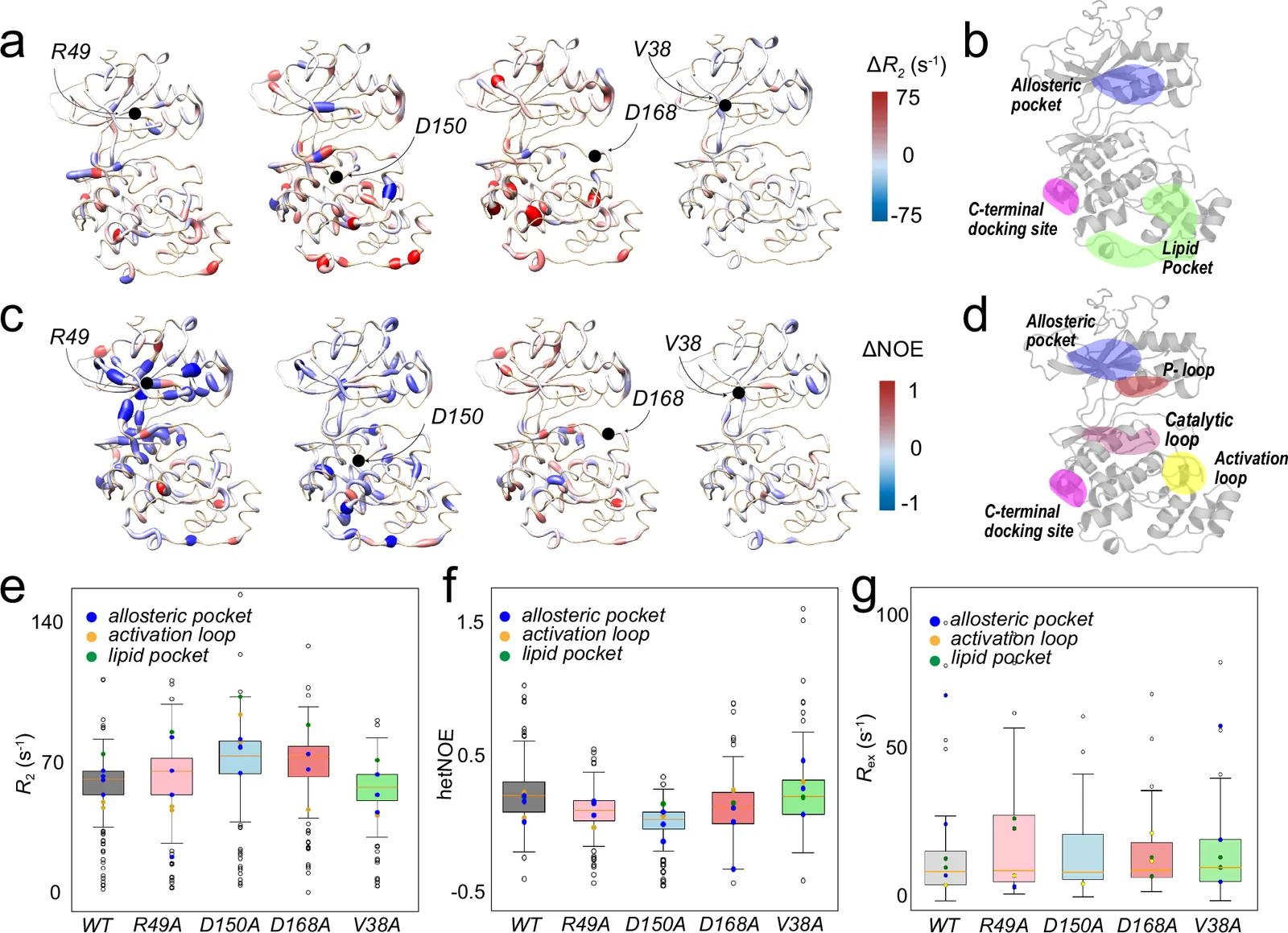
Changes in p38α dynamics, as seen by NMR relaxation. **a** Structural mapping of Δ*R*₂ (mutant – WT) values onto the three-dimensional structure of p38α kinase. Red regions indicate residues with *R*₂ higher in the mutants, predominantly localized to the catalytic core and distal C-lobe, supporting widespread conformational exchange. **b** Dominant regions of protein showing an increased *R*_*2*_ upon mutation of the dynamic network (across R49A, D150A, and D168A), also specifically including the lipid pocket. **c** Structural visualization of ΔhetNOE values. Blue regions highlight residues with reduced NOE values in the mutants, indicative of enhanced backbone flexibility. **d** Regions portraying increased fast time-scale dynamics consistent across dynamic network mutants**. e, f** Box plots showing the residue-level distributions of *R*_*2*_ and hetNOE values, respectively, for WT, R49A, D150A, D168A, and V38A. The numbers of residues included were *n* = 225 for WT, *n* = 210 for R49A, *n* = 198 for D150A, *n* = 185 for D168A, and *n* = 220 for V38A. **g** Box plots showing the residue-level distributions of *R*_*ex*_ values. The numbers of residues included were *n* = 46 for WT, *n* = 37 for R49A, *n* = 40 for D150A, *n* = 33 for D168A, and *n* = 45 for V38A. For panels e–g, boxes extend from the 25th to the 75th percentiles, and the center line indicates the median. Whiskers extend to the most extreme values within 1.5 times the interquartile range. Values beyond the whiskers are plotted individually as outliers. The minimum and maximum correspond to the lowest and highest plotted observations, respectively, including outliers. Each data point represents one residue within the corresponding NMR dataset and not an independent biological replicate. Data were obtained from one independently expressed and purified protein sample per construct and are presented descriptively. Colored points identify residues belonging to the activation loop, allosteric pocket, and lipid-pocket/MAP kinase insert regions. The network mutants showed higher median *R*_*2*_ values and lower median hetNOE values than WT and V38A, whereas the *R*_*ex*_ distributions were broadly similar across constructs. Source data are provided as a Source Data file.

To determine whether the observed chemical-shift perturbations really reflect changes in global protein dynamics, we employed solution-state ^15^N relaxation, more specifically, [^15^N, ^1^H] heteronuclear NOE (hetNOE) and ^15^N transverse relaxation rates (*R*_*2*_). These partly complementary relaxation parameters probe site-specific motion on the ps-ns timescale, with *R*_2_ being additional influenced by motion on the µs timescale. Among all mutants tested, D150A exhibited the most pronounced deviations from wild-type behavior across both hetNOE and *R₂* datasets (Fig. 5a–f and Supplementary Fig. 19). The further reduction in hetNOE values compared to the wildtype at several positions throughout the protein suggests a further increase in fast-timescale local flexibility. In parallel, increased *R*₂ values, especially in the C-lobe (residues around 200 – 250, which accommodate the lipid pocket), indicate an additional increase of conformational-exchange contributions, stemming from increased µs timescale fluctuations and suggesting a widely altered energy landscape introduced by the mutation. These observations also align with significant exchange broadening, which is usually incurred by enhanced µs–ms conformational dynamics. The differences to the wild type are also visualized through structural mapping of per-residue deviations (Fig. 5a, c), witnessing dynamic perturbations extending well beyond the respective mutation sites. Intriguingly, D168A and R49A also show vivid alterations in the C-lobe regarding the *R*_2_ rates, and more slight alterations for hetNOE values, again supporting that these sites represent nodes within a network of long-range allosteric couplings. Also, the extent of dynamic reorganization in D150A appears to be globally distributed. Collectively analyzing *R*_*2*_ differences across all network mutants relative to WT revealed recurrent changes in the lipid-pocket/MAP kinase insert region, consistent with dynamic coupling of this pocket to the broader kinase network (Fig. 5b and Supplementary Table 11) and coherent with the recent findings within p38α ligand binding studies^18^. The hetNOE comparison further showed shared perturbations in several regulatory elements, including the activation-loop boundary, HRD/catalytic-loop-adjacent region, and P-loop/glycine-rich loop (Fig. 5d, Supplementary Table 12). Previous studies have identified these same structural elements as dynamic and functionally coupled regulatory sites for this protein. The activation loop and its N-terminal boundary undergo phosphorylation- and inhibitor-dependent conformational rearrangements^17^, the HRD/catalytic-loop-adjacent residue D150 participates in communication with the activation-loop residue T185 during non-canonical autophosphorylation, and the P-loop/glycine-rich loop, including Y35, samples distinct conformations linked to ATP-site accessibility^5,53^. The C-terminal docking groove also shows recurrent perturbation in the relaxation data, for both hetNOE and *R*_*2*_, suggesting that the MAPK docking surface involved in TAB1-mediated regulation is dynamically coupled to the broader allosteric network, corroborating earlier findings^19,54–56^. Thus, although the global distributions of motional parameters as shown in the box plots overlap, the site-specific analysis identifies recurrently affected regulatory sectors, supporting an allosteric entanglement of the lipid pocket and canonical kinase-regulatory regions within the p38α dynamic network. In disparity, the control variant V38A, with its mutation site outside the predicted dynamic network, exhibited again only minimal, local changes. Both its hetNOE and *R₂* profiles remain largely aligned with the wild-type except for subtle shifts near the site of mutation (Supplementary Fig. 19), confirming its neutrality regarding the kinase’s conformational ensemble. To probe conformational-exchange processes on the microsecond-to-millisecond timescale, we finally performed ^15^N CPMG relaxation dispersion (RD) measurements on wild-type protein and the mutants (Supplementary Fig. 20). Data quality was insufficient to perform a more comprehensive analysis, but exchange contributions *R*_*ex*_ to the effective transverse relaxation values could be obtained with reasonable accuracy, offering residue-specific identification of transient conformational substates. In RD experiments, generally, the exchange contribution *R*_*ex*_ is derived from the difference in *R*_*2eff*_ measured at varying CPMG refocusing efficiency. High *R*_*ex*_ values indicate residues undergoing chemical exchange between conformers on the microsecond-to-millisecond timescale, such as allosteric transitions, functional loop dynamics, or domain-breathing motions. *R*_*ex*_ can increase when the population of an excited state is higher, when the chemical-shift difference between exchanging states is larger, or both. Globally, wild-type samples exhibited the largest number of residues with significant *R*_*ex*_ contributions throughout the N-lobe and the C-lobe, indicating widespread conformational plasticity (Supplementary Fig. 20a, b). As expected, a highly similar pattern as for the wild type is observed for V38A, both with respect to the residue level (Supplementary Fig. 20a) and regarding the distribution of *R*_ex_ contributions depicted in the form of a histogram (Supplementary Fig. 20b), consistent with its position outside the dynamic network and confirming its dynamically silent nature in terms of allosteric communication. The mutant construct R49A shows a slight loss of ms timescale exchange in the N-lobe and a slight increase in the C-lobe, confirming the above reorganization of motional behavior (Supplementary Fig. 20c). For mutants D150A and D168A, by contrast, a slight decrease of *R*_*ex*_ in the C-lobe and a slight increase in the N-lobe was observed (Supplementary Fig. 20c). All of these changes are, however, rather sporadic and any trends are difficult to discern. The apparent lack of strong differences between the samples results on the one hand from a rather noticeable error associated with the individual *R*_*2eff*_ rates in these experiments. Supplementary Fig. 20d exemplifies this for A172, a residue lying in between the DFG motif and the activation loop, via comparison of dispersion curves for the different samples. A number of other dispersion curves are shown in Supplementary Fig. 21. However, the higher overall similarities across the *R*_*ex*_ histograms in Supplementary Fig. 20b suggests that the differences induced by the perturbations to the dynamic networks, which were consistently seen upon CSPs, hetNOEs, and *R*_*2*_ analyses, are rather limited to motions faster than the ms regime. Boxplot comparisons across the variants (Fig. 5e–g) support a general enhancement of this fast-timescale flexibility (decreased hetNOE), including a concurrent rise in µs timescale dynamics (higher *R*₂ values), while *R*_*ex*_ remain more similar. The overall picture suggests that mutations in dynamic network positions generally increase protein plasticity rather than promoting large-scale conformational exchange. These observations are consistent with a loosening of the dynamic network upon mutation, upon which motion of the individual residues becomes slightly less coupled and hence less restricted overall.

## Discussion

The results presented here demonstrate that p38α kinase harbors a dynamic allosteric network that connects the N- and C-lobes and explicitly incorporates the lipid-binding domain and its associated lipid pocket. Mutations at residues identified as central nodes within this network induce substantial perturbations in both local and global dynamics, as evidenced by a consistent set of computational and experimental observations, including molecular dynamics simulations, dynamical network analysis, pathway-level correlation changes, chemical-shift perturbations, heteronuclear NOE measurements, and transverse relaxation rates. While ligand binding partially modulates these effects, the dominant impact of network disruption is observed in the apo ensembles. Collectively, these findings provide experimental validation for the simulation-derived prediction that the p38α scaffold supports long-range dynamic communication between distinct functional regions of the protein. Note that the accuracy of dynamic network analysis in general and for a biological system as complex as a kinase is not only limited by the force fields and duration of sufficiently long MD simulations but also due to the manifold chemical states that the kinase cycles through in a physiological context. Even with the general confirmation of the network by complementary techniques, the insights hence need to be understood as rather qualitative, i.e., confirming the existence and giving the approximate extent of the network, not as a precise reproduction of physiology. Still, this framework helps rationalize previous observations on phosphorylation-dependent activation, TAB1-mediated regulation, the global impact of lipid-pocket binding ligands, and kinase dynamic allostery generally. Because our simulations and experiments were performed in the unphosphorylated apo state, they do not reproduce the full phosphorylated or TAB1-bound activation pathways. Still, they show the existence of a basal communication architecture from which these regulatory mechanisms may emerge. Whereas the firm connectivity between dynamic-network residues tethers the plastic kinase architecture tightly together, thereby conducting concerted dynamics across long distances, mutations at key network sites abrogate long-range communication, entailing increased fast-timescale flexibility and conformational exchange. This conclusion can rationalize prior observations in a more biological context in a residue-specific manner. For example, local perturbations, such as activation-loop phosphorylation or binding of upstream regulators, can induce dynamical changes at distal sites within the kinase, albeit on slower timescales than those primarily interrogated here^17^. With the revelation of a widespread dynamic network prominently involving the activation loop and encompassing, in particular, the active site, the top part of the C-lobe, and the lipid pocket, the various prior observations hinting to dynamic allostery across the kinase architecture and regulation come to no surprise. Also interestingly, the partial quenching of the allosteric network by sorafenib binding observed here in silico nicely fits to the experimentally observed reduction of CSPs and relaxation effects otherwise induced upon lipid pocket occupation by the active-site inhibitor^18^.

Residues identified as key network nodes impose long-range effects on the regulatory elements previously implicated in p38α activation, TAB1-mediated autoactivation, nucleotide-site control, and kinase pathology. D150, located in proximity to the catalytic site^57^ of the protein, forms an intramolecular H-bond with T185 that is required for TAB1-induced autoactivation^58^. R49K/A is used as a convenient tool mutation for studying PRMT1-mediated control of p38α^59^. Also, D168 mutation is known to render the enzyme dysfunctional and has been used as a valuable mechanistic probe^60–62^. Physiologically, its prominent role for the in/out conformational switch of the DFG motif, required for exchanging ATP/ADP at the active site, clearly exceeds the role as a mere dynamics-transducing element of the network. It is still important to realize that this site is part of the general, long-range communication, transducing information across the protein scaffold. This suggests that any modulations of the DFG motif may also have a long-range impact on the kinase’s motional properties and modulate conformational states far away from the site. Importantly, our data establish the lipid-binding domain as an integral component of the p38α dynamic network, which corroborates an allosteric connectivity between the lipid pocket and other regulatory units of the protein and supports prospects for pharmacological avenues based on lipid-pocket-binding allosteric modulators^9^. Taken together, these observations demonstrate how widespread dynamic networks, rather than the local mobility associated with the individual structural elements, underlie the integration of regulatory signals in p38α and likely in other kinases as well. The comparison with p38γ and other kinases in our study places the present p38α network within a broader framework of kinase dynamic allostery. Previous studies established that conserved kinase motifs, including the DFG segment, HRD catalytic motif, hinge region, regulatory spines, and substrate-binding regions, can participate in long-range communication. Our study extends this framework to p38α by providing a residue-resolved and experimentally supported dynamic-network map. Importantly, this network explicitly incorporates the MAP kinase-specific lipid-binding domain and its associated cryptic lipid pocket. Thus, the lipid pocket does not seem a mere distal/isolated binding cavity, but does present itself as a dynamically integrated regulatory region coupled to canonical kinase-control elements. Beyond the sheer identification/mapping of communication pathways between the p38α N- and C-lobes in the presence and absence of the inhibitor, the above results may also offer a conceptual framework for identifying additional dynamically connected allosteric regions in p38α. In the long term, the knowledge about, in particular, the existence and whereabouts of surface sites associated with its dynamic networks could facilitate the design of allosteric modulators that do not target the known pockets, such as the ATP binding site or the “allosteric pocket”, hence avoiding the common off-target effects of kinase inhibition.

For the lipid pocket, it is still unclear whether it can ever be addressed pharmacologically. Despite some initial trials to address the lipid pocket, binders to this site have not been able to impose any functionally relevant changes to the kinase. With first-generation, low-affinity binders (lipid pocket ligands) in particular, lipid pocket occupation (at the low populations obtained) has not been found to bear a strong impact on kinase activity^9^. Still, the confirmation that a dynamic network extends to this site and in particular the identification of W207 at the rim of the lipid pocket may be interpreted as a motivation to re-evaluate the theoretical possibility that the site may serve as a starting point for alternative allosteric p38α effectors. Given that for a desired systemic effect even a weak molecular impact can suffice, the above data encourage further work, involving bulkier or sterically optimized ligands, which might result in distinct perspectives to overcome the off-site effects usually associated with targeting kinases. Note, however, that the above data do not establish this pocket as a pharmacologically validated target. Such a conclusion would require additional studies using optimized ligands, direct functional assays, and selectivity or pathway-level validation. More generally, awareness and spatial mapping of dynamic networks in enzymes may guide the identification of regulatory sites amenable to therapeutic intervention or rational engineering. It remains to be seen whether dynamic allostery will prove valuable not only for pharmacological inhibition but also for fine-tuning of enzymatic activity in biotechnological applications through targeted allosteric mutations^63–65^.

In summary, we identify and map a dynamic allosteric network within p38α MAP kinase that spans both lobes, prominently involves inter-lobe segments of the architecture and the activation loop, and explicitly includes the lipid-binding domain and its associated lipid pocket. By integrating computational and experimental structural biology approaches, we reveal specific residues that act as key information relays within a coordinated motional framework, transmitting dynamics across spatially distant regions of the protein. Mutations performed at some of these hotspots indeed largely corrupt the integrity of the network, thereby significantly reducing inter-lobe communication and inducing global changes in residue-specific dynamics, underscoring their central role in long-range allostery. Together, these findings provide insights into the dynamic basis of kinase regulation and pathology. With the lipid pocket surface, in particular residue W207, found to form an integral part of the dynamic network, the study also highlights opportunities for identifying and targeting allosteric sites in future pharmacological strategies.

## Methods

### Dynamical network analysis

All atomic molecular dynamics (MD) simulations were performed using NAMD 3.0^33^ in conjunction with the QwikMD plugin^66^. To quantify ensemble-level differences associated with the reported ligand-dependent conformational changes, simulations were initiated from two crystallographic structures of p38α in which the inhibitor ring flip was observed (PDB IDs: 3HEG [https://doi.org/10.2210/pdb3HEG/pdb]^15^ and 3GCS [https://doi.org/10.2210/pdb3GCS/pdb]^16^). For apo p38α (1WFC [https://doi.org/10.2210/pdb1WFC/pdb]^34^), missing segments were rebuilt by comparative modeling. NOESY-derived restraints were applied during energy minimization (20,000 steps), slow heating to 300 K, and a ~ 1 μs restrained equilibration; restraints were then released and five independent 1 μs production replicas were generated. Dynamical network analysis identified betweenness-centrality hotspots, which were alanine-scanned using QwikMD; each mutant underwent the same minimize–anneal–equilibrate protocol, followed by five 1 μs replicas for correlation and network metrics. Sorafenib was docked into the allosteric pocket (Supplementary Table 7), prepared identically, and simulated in five 1μs replicas. Correlations and communication pathways were compared across apo, mutant, and ligand-bound ensembles. A total of ~50 μs simulations were used for this study.

All simulations employed the CHARMM36^34^ force field for proteins and ligands. Covalent bonds involving hydrogen atoms were constrained using SHAKE, and equations of motion were integrated using a 1 fs time step. Long-range electrostatics were treated using the particle-mesh Ewald method under periodic boundary conditions, with a real-space cutoff of 12 Å and a pair-list distance of 14 Å. Systems were equilibrated under NVT conditions for thermalization and NPT conditions for pressure coupling prior to production. Temperature was maintained at 300 K using Langevin dynamics, and pressure was controlled at 1 atm using the Langevin piston method under periodic boundary conditions.

The topology and trajectory files from the MD simulations were incorporated into the Python notebooks of Dynetan^27^. The software uses the incorporation of MDAnalysis to analyze the trajectory simulation files^67^. Contact detection optimization of the code was already performed via Numba^68^ and Cython^69^. Network statistics and the determination of optimal paths were carried out using the Floyd–Warshall algorithm, provided by the NetworkX package. For Floyd–Warshall calculations, the “distance” between nodes was defined as

1 $$d=-\log \left({r}_{{MI}}\right),$$

which uses mutual information as a method for contact detection consistent with previous applications of this method which can be seen as:

2 $${r}_{{MI}}\left[i,j\right]={\left(1-{e}^{-2/3I\left[i,j\right]}\right)}^{1/2}$$

where i and j are the position of the alpha carbon of each residue and *I*[*i,j*] are the mutual information between them which is computed by the density estimator described as:

3 $$I\left[i,j\right]=\psi \left(k\right)-1/k- \left\langle \psi \left({n}_{i}\right)+\psi \left({n}_{j}\right)\right\rangle+\psi \left(N\right)$$

where here N is the total number of simulation frames, ψ (x) is the digamma function, n_i_ is the number of frames in which residue i is close to the one in the reference, and stands for the average of the trajectory, using a neighboring parameter k of 6.

Each amino acid residue is regarded as a node. The network is structured by these nodes and nodes lie within a cutoff distance (4.5 Å) for at least 75% of an MD trajectory. The residues that lie farther than the cutoff value are excluded, which mainly includes some solvent and ion molecules. The nodes are connected via links known as “edges”.

Betweenness centrality was computed as:

4 $${b}_{i}=1/c\,{\sum }_{s,t\epsilon v}\sigma \left(s,t,|,i\right)/\sigma (s,t)$$

where *σ* (*s*, *t*) is the number of shortest paths between nodes *s* and *t*, *V* is the ensemble of graph nodes, and *c* is a normalization factor.

The simulations were analyzed using in-house Python and TCL scripts along with VMD^70^. Contact maps for residue–residue interactions were generated with VMD and PyContact. For the statistical testing, we normalized all measurements by the cumulative WT (or liganded WT) row. For each variant, we report the per-variant mean and plot error bars as the sample standard deviation across replicates. Group comparisons with WT were performed using a two-sided non-parametric permutation test. The two groups were pooled, group labels were permuted, and the difference in means was recalculated over 10,000 permutations. The *p* value was calculated as the proportion of permuted differences whose absolute value was equal to or greater than the observed absolute difference. The observed difference in mean normalized correlation was used as the test statistic. No adjustment for multiple comparisons was applied. Hedges’ was calculated as the standardized effect size, and 95% bootstrap confidence intervals were calculated for the mean differences. No parametric assumptions (normality or equal variances) were required and plotted using Python notebooks. All the structural figures for simulations are rendered through VMD. The figures showing residue-wise properties are extracted through Chimera attributes.

### *Sample Preparation*

The plasmid map of the kinase was elucidated by nanopore sequencing to entail the specific design of mutagenesis primers. The primers for the mutants were designed such that the mutation residue lies at the center of the forward and reverse primers. *Phusion™ High-Fidelity DNA Polymerase*, a fusion polymerase containing a Mastermix of nucleotides, enzymes, and buffers, was used to carry out the polymerase chain reaction. The PCR product was digested using the DpnI enzyme at 37 °C for 1 hr and was immediately transformed overnight at 37 °C to *E.coli XL 10 gold* cells. The transformed colonies were grown in an overnight culture at 37 °C and the plasmid was extracted using a PEG gold miniprep kit and sent to Eurofins for Sanger Sequencing. The wild type as well as the mutants were expressed in BL21(DE3) *E. coli* cells using ^13^C and ^15^N labeling for assignment and relaxation experiments. D_2_O adaptation was carried out, and the cultures were grown in 1 L at 37 °C to reach the OD_600_ of 0.6-0.7. They were then cooled for 30 min to room temperature and induced with 1 mM IPTG overnight (∼20 h) at 18 °C while shaking at 180 rpm. For the mutant W207A, expression in deuterated minimal media could not be achieved. The cells were lysed using a 50 mM Tris buffer, benzonase, and lysozyme in the presence of a protease inhibitor, centrifuged, and the supernatant was exposed to affinity chromatography (binding buffer: 50 mM Tris, 500 mM NaCl, 25 mM imidazole and 5% glycerol; elution buffer: 50 mM Tris, 500 mM NaCl, 500 mM imidazole, and 5% glycerol) using a nickel column. The eluted fractions were dialyzed to a pH of 7 and were subjected to an overnight cleavage in the presence of thrombin. Anion exchange (binding buffer: 25 mM Hepes and 5% glycerol; elution buffer: 25 mM HEPES, 1 M NaCL, 5% glycerol) and Size Exclusion Chromatography (SEC buffer: 20 mM HEPES, 50 mM NaCl, 100 mg/L methionine and 5% glycerol) were then used to obtain pure protein.

### HTRF Analysis

Wild-type and mutant p38α constructs were activated with constitutively active MKK6 S207E/T211E (Thermo Scientific, lot no. 877061 F) in activation buffer containing 50 mM Tris, 10 mM MgCl_2_, 1 mM ATP, 1 mM DTT and 0.001% Tween-20, pH 7.4. Activation was performed at 37 °C for 90 min with shaking at 400 rpm. The reaction mixtures were subsequently dialyzed overnight at 4 °C against storage buffer containing 20 mM HEPES, 50 mM NaCl and 5% glycerol, pH 7.1, concentrated to approximately 0.2 mg/mL and stored at −80 °C. HTRF-based activity measurements were performed using the HTRF KinEASE assay format. Reactions were carried out in white, small-volume, medium-binding, flat-bottom 384-well plates (Greiner Bio-One, catalog no. 784075). For determination of ATP *K*_M_ values, activated p38α was added at 0.04 ng per well for WT, 3.5 ng per well for unlabeled mutant constructs or 20 ng per well for labeled mutant constructs. The proteins were incubated with 1 µM GST-ATF2 substrate and ATP concentrations ranging from 0.4 to 900 µM in reaction buffer containing 50 mM HEPES, 0.1 mM Na_3_VO_4_, 0.02% NaN_3_, 0.01% (w/v) BSA, 10 mM MgCl_2_, 1 mM MnCl_2_, 1 mM DTT and 0.01% Triton X-100, pH 7.0. WT, unlabeled mutant and labeled mutant reactions were incubated for 10, 10 and 20 min, respectively. For substrate titrations, increasing concentrations of GST-ATF2 were incubated with activated WT or mutant p38α in the same reaction buffer, using a constant ATP concentration. Substrate-dependent responses were fitted using the Hill equation. One activated protein preparation was used per construct, and each ATP or substrate concentration was measured in four technical replicate wells. Reactions were stopped by adding detection solution containing 50 mM HEPES, 0.1% (w/v) BSA, 800 mM KF and 20 mM EDTA, pH 7.0. Phosphorylated GST-ATF2 was detected using HTRF pAb anti-phospho-ATF2 Thr69/71–Eu conjugate (Cisbio/Revvity, catalog no. 61P12KAZ; 1:200 working dilution; concentration in the detection solution, 0.666 nM) and HTRF mAb anti-GST–d2 conjugate (Cisbio/Revvity, catalog no. 61GSTDLB; reconstituted concentration, 400 µg/mL, corresponding to approximately 2.67 µM; approximately 1:27 dilution; concentration in the detection solution, 100 nM). Plates were incubated for 60 min at room temperature before measurement. Time-resolved fluorescence was measured using an EnVision 2104 plate reader (PerkinElmer) at emission wavelengths of 620 and 665 nm, 60 µs after excitation at 317 nm. The f665/f620 fluorescence ratio was calculated for each well. Data points represent the arithmetic mean of four technical replicate wells. ATP-dependent responses were fitted using the Michaelis–Menten equation, and substrate-dependent responses were fitted using the Hill equation in Origin (OriginLab). Curves shown in the main figure were normalized to the fitted *v*_max_ of the corresponding protein construct, whereas non-normalized responses are shown in Supplementary Fig. 16. The official 61P12KAZ manual specifies a 1:200 working dilution for the 10,000-test format. Revvity identifies 61GSTDLB as the 20,000-assay-point anti-GST–d2 product, and its available technical sheet specifies reconstitution in 1 mL water to an active-moiety concentration of 400 µg/mL.

### Solution NMR

The protein from the SEC Buffer was exchanged to NMR Buffer containing 50 mM of HEPES and 150 mM NaCl (pH ~6.8), and the sample was concentrated to approximately 450 µM. NMR experiments were recorded on an 800 MHz Bruker NEO spectrometer. 2D ^15^N-^1^H TROSY HSQC and 3D TROSY HNCA were recorded to transfer assignments from the BMRB (entry: 17471 [https://doi.org/10.13018/BMR17471]) to the mutants. ^15^N-^1^H TROSY hetNOE was performed to assess fast-timescale dynamics using interscan delays of 5 and 8 s in scans with or without 3 s of proton saturation, respectively. ^15^N TROSY-CPMG experiments were acquired in an interleaved manner as follows for the CPMG frequencies: 0, 2000, 25, 1500, 1000, 100, 750, 200, 1250, 500, 200 Hz with a CPMG delay of a total of 0.022 s and an interscan delay of 1.5 seconds. The experiments were analyzed with NESSY software^71^. Graphs and analysis were produced using in-house Python scripts.

### NMR Data Analysis

CCPN version 3.1 was used for assigning the mutant peaks^72^. Chemical-shift perturbations were obtained as √(δ_H_^2^ + δ_N_^2^/100 + δ_Ca_^2^/16). ^15^N-^1^H TROSY-NOESY was analyzed using a CCPN macro for relaxation. ^15^N TROSY-CPMG experiments were analyzed using NESSY software^71^. Regions collectively affected by the dynamic-network mutations were identified by mapping the perturbations onto predefined protein regions and selecting those that were affected in more than one mutant. Graphs and analysis were produced using in-house Python scripts.

### Reporting summary

Further information on research design is available in the Nature Portfolio Reporting Summary linked to this article.

## Supplementary information

Supplementary Information

Reporting Summary

Transparent Peer Review file

## Data Availability

All raw data have been deposited in the public repository of Technical University Dortmund (https://doi.org/10.17877/RESOLV-2026-MK5LFITB) and can be freely accessed. Previous crystal structure data used in this study are PDB 3GCS, 1WFC and PDB 3HEG. Previous assignment data used here are listed under BMRB 17471.
