# Supplementary material for: Mapping of dynamic allostery within p38 alpha kinase via network analyses and NMR spectroscopy
Source: Nat Commun. 2026 Aug 22;17:10069. doi: 10.1038/s41467-026-76885-7 (PMC13594348; doi:10.1038/s41467-026-76885-7)
Supplement: Supplementary file 1 [file 41467_2026_76885_MOESM1_ESM.pdf]

Supplementary Information for

Mapping of dynamic allostery within p38 alpha kinase via network analyses and NMR spectroscopy

Suchandra Roy Acharyya, Jörn Weisner, Rafael C. Bernardi, and Rasmus Linser

Table of contents - Figures

Figure Number	*Caption*	*Page No*
1	Schematic representation of the methodology to identify the communicators and validate them experimentally and computationally.	S2
2	Backbone RMSD and RMSF of the replicas of apo p38α	S2
3	RMSD and RMSF of ligand bound crystal structures of p38α	S3
4	Dynamical network analysis of MD trajectories starting with the crystal structures	S3
5	Beta factor analysis	S4
6	RMSDs and RMSFs of 5 replicas per mutant.	S5
7	Network analysis by Dynetan, mainly betweenness and communities of the mutants	S6
8	Total correlation map of the WT and the mutants	S7
9	N-C lobe correlations of the mutants using the correlation	S7
10	Differences (Mutant – WT) in the correlations from the N to C lobe for each of the mutants	S8
11	Interacting residues when ligand Sorafenib is docked with the WT protein and the mutants	S8
12	RMSDs and RMSFs of 5 replicas per ligand-bound mutant	S9
13	Correlations of the ligand with the entire protein for WT and mutants	S10
14	Differences in the pathways and their respective cumulative correlations upon drug binding, using apo and holo pathways as a reference.	S11
15	Preparation of WT and mutant samples for p38α	S12
16	Raw output of HTRF data	S12
17	Transfer of assignments using TROSY HSQC and HNCA spectra of the WT	S13
18	TROSY-HSQC overlay of the mutants	S13
19	Residue-wise hetNOE and R_2 representation and comparison of the values	S14
20	Slow timescale analysis of the mutants with the WT	S15
21	CPMG profiles of the wildtype and the mutants	S18

Table of contents - Tables

Table Number	*Caption*	*Page No*
1	Summary of Molecular Dynamics system parameters	S18
2	Residues showing highest betweenness centrality for the wildtype and mutants	S19
3	Correlation Values Between N-lobe to C-Lobe	S19
4	Normalised beta factors for network residues	S20
5	Pathways to and from different pockets	S21
6	Statistical values for shortest-path analyses	S22
7	Docking results of WT and mutant with Sorafenib at the allosteric pocket	S23
8	K_M, v_{max} and EC_{50} values	S24
9	Mean CSP and their calculated error using Linewidth	S24
10	Important regions with high CSP for all mutants	S24
11	Important regions around the protein as shown by all mutants determined by difference in R_2	S25
12	Important regions around the protein as shown by all mutants determined by difference in R_2	S26

Supplementary Figures

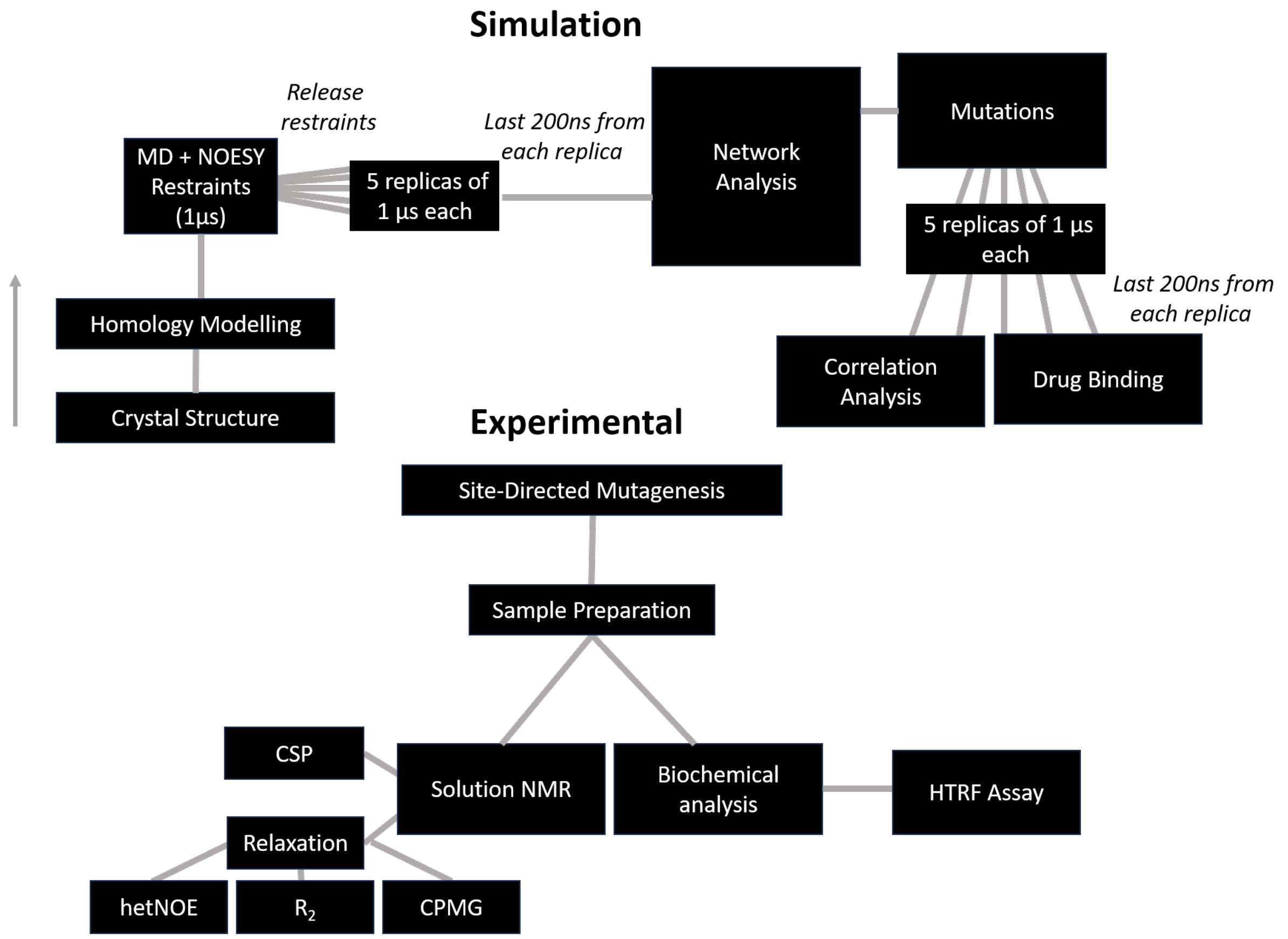

Supplementary Figure 1: Schematic representation of the methodology to identify the communicators and validate them experimentally and computationally.

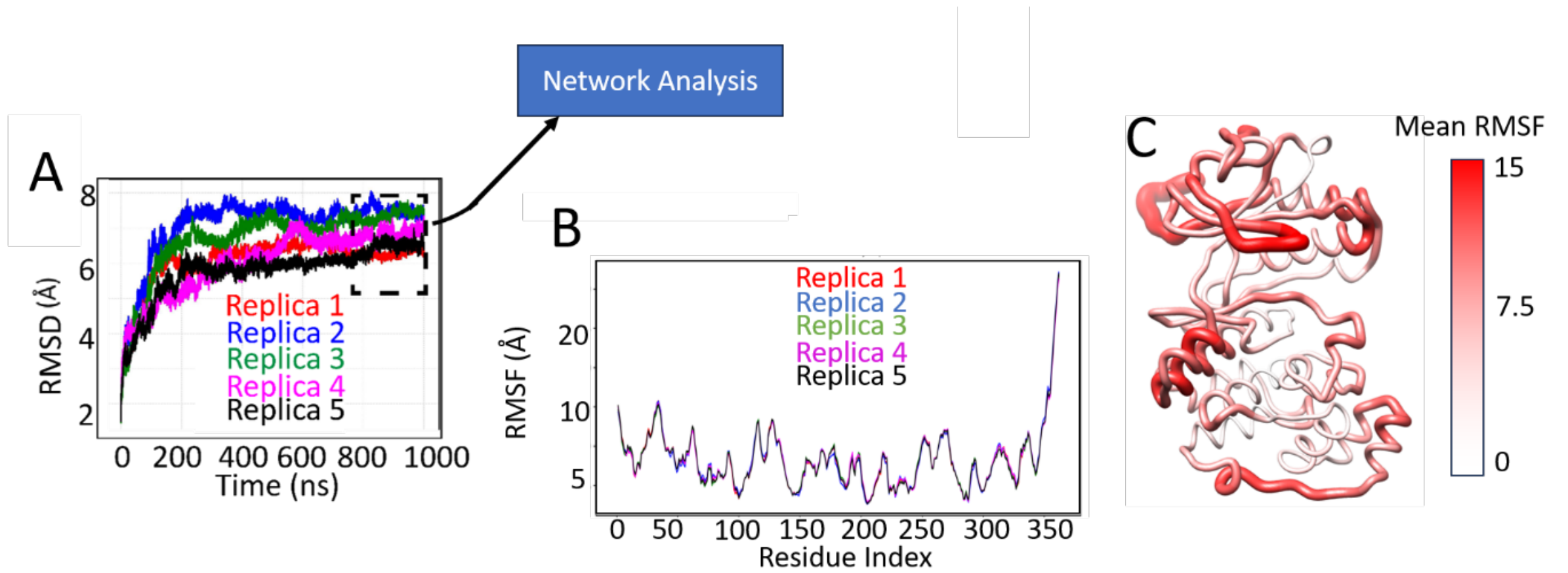

Supplementary Figure 2: **A**) Backbone RMSD of the apoprotein across five independent 1 μs replicas; the final 200 ns of each replica were used for network analysis. **B**) Overlay of RMSD traces for all five apoprotein replicas, highlighting convergence. **C**) Average per-residue RMSF mapped onto the protein structure to visualize flexibility.

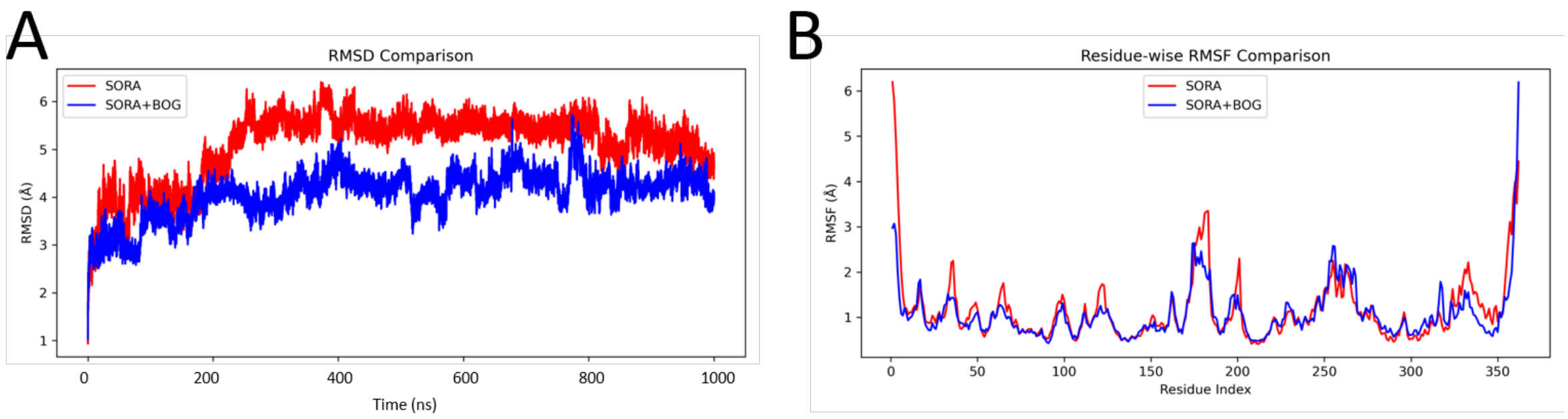

Supplementary Figure 3: A) RMSD of the simulations using starting crystal structures 3HEG (p38a bound to sorafenib only; red) and 3GCS (Sora + BOG; blue) **B**) RMSF of the simulations using starting crystal structures 3HEG (Sorafenib with p38α; red) and 3GCS (Sora + BOG with p38α ; blue).

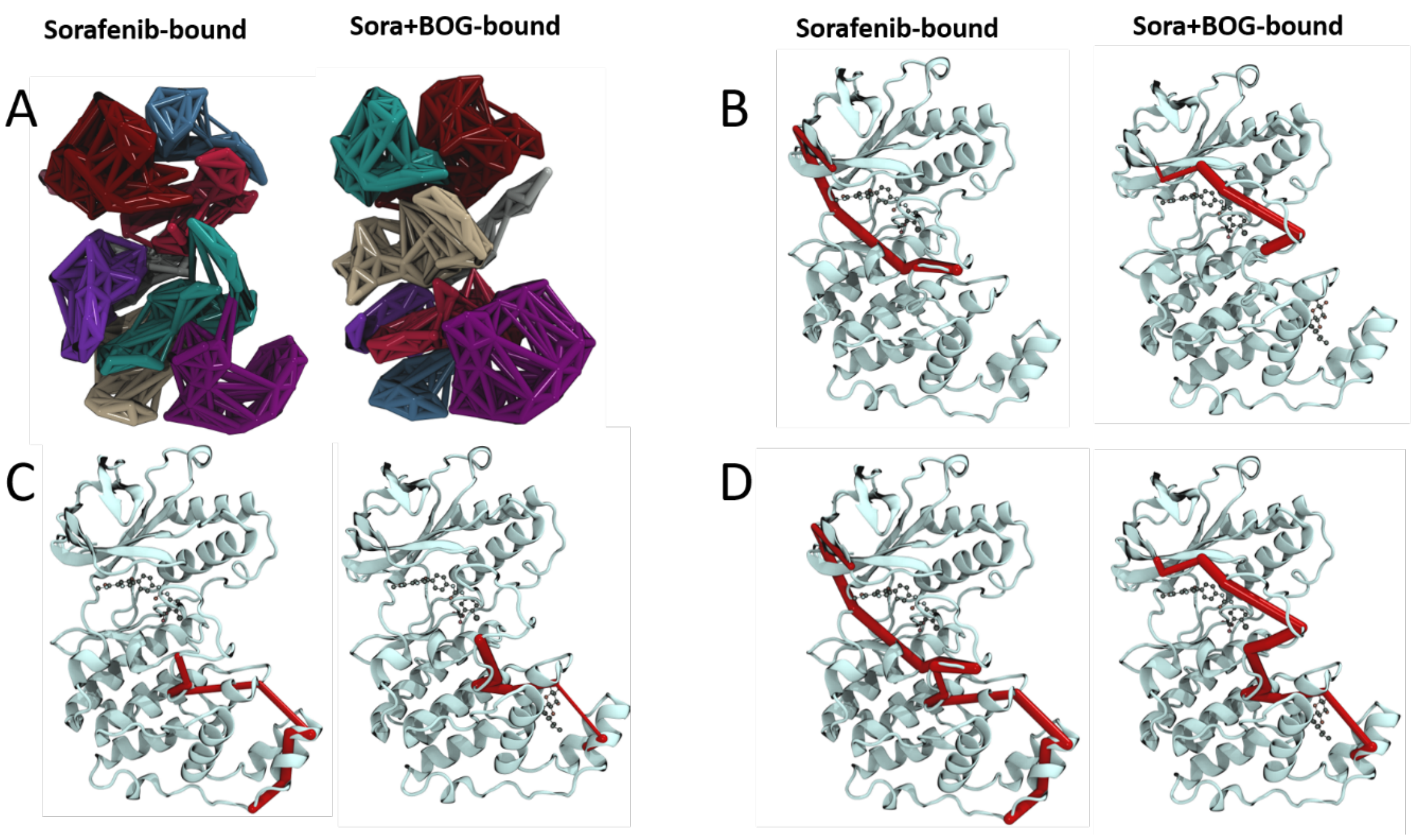

Supplementary Figure 4: Dynamical network analysis of MD trajectories starting with the crystal structures. A) Differences in communities when BOG binds to the lipid pocket. **B**) Differences in pathways upon BOG binding from the allosteric pocket to the activation loop. **C**) Differences in pathways upon BOG binding from the lipid pocket to the activation loop. **D**) Differences in pathways upon BOG binding from the lipid pocket directly to the allosteric pocket.

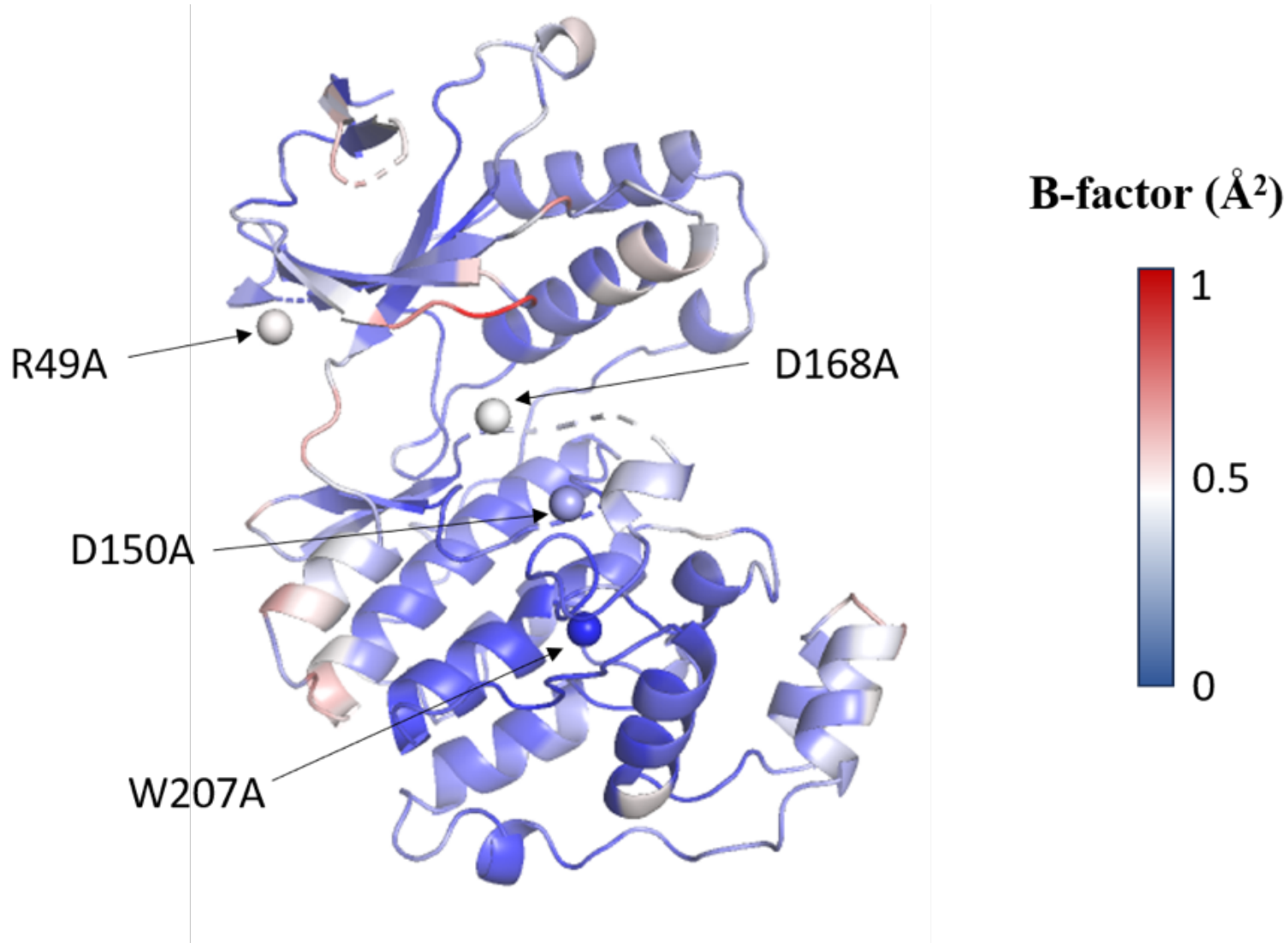

Supplementary Figure 5: B-factors of X-ray crystal structure along the protein. Temperature B-factors (Debye-Waller factors) are shown for the crystal structure PDB 3HEG.

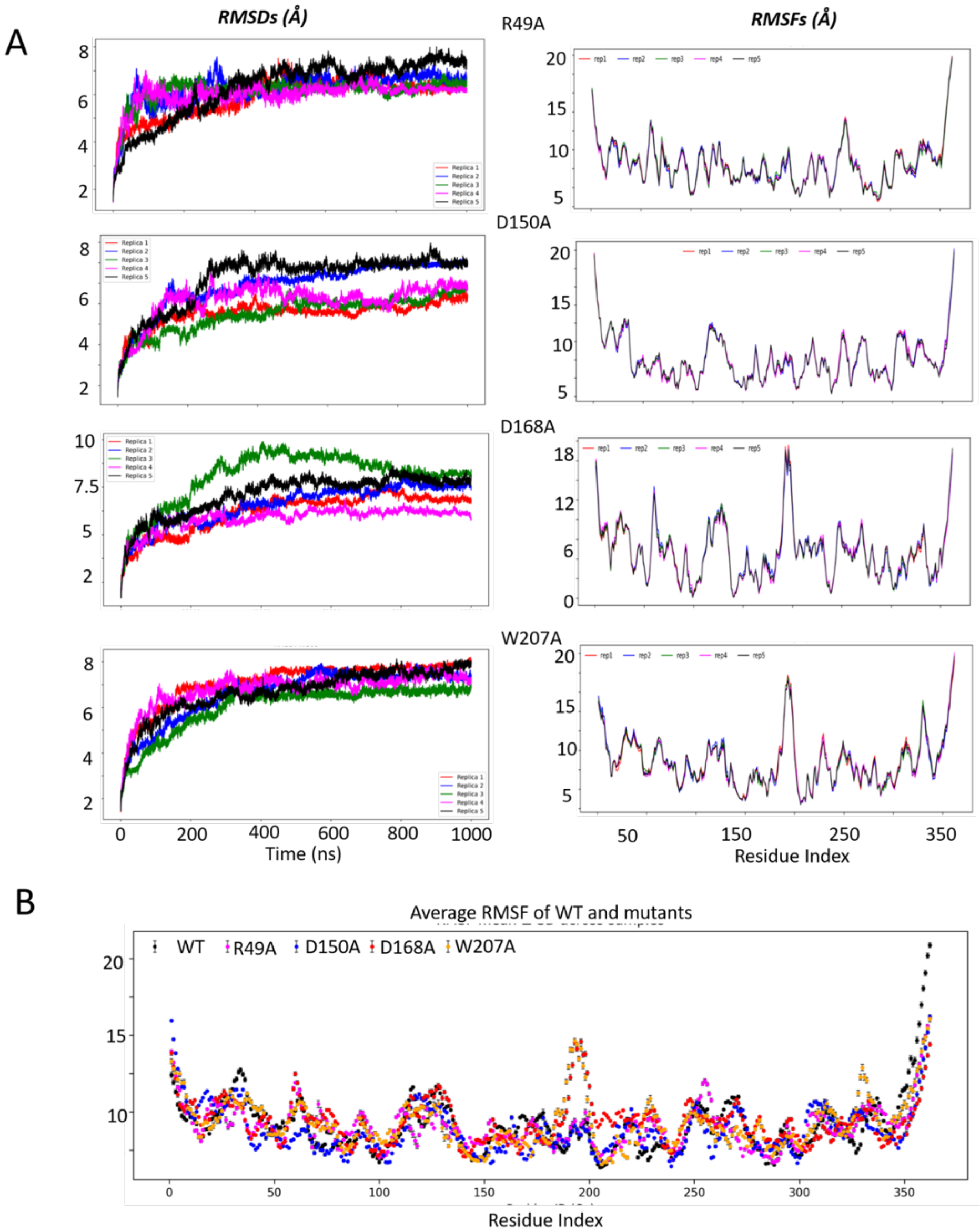

Supplementary Figure 6: A) RMSDs and RMSFs of five replicas per mutant. The last 200 ns were used for network and correlation analysis. **B)** Mean and SD of all the RMSFs compared with the wildtype.

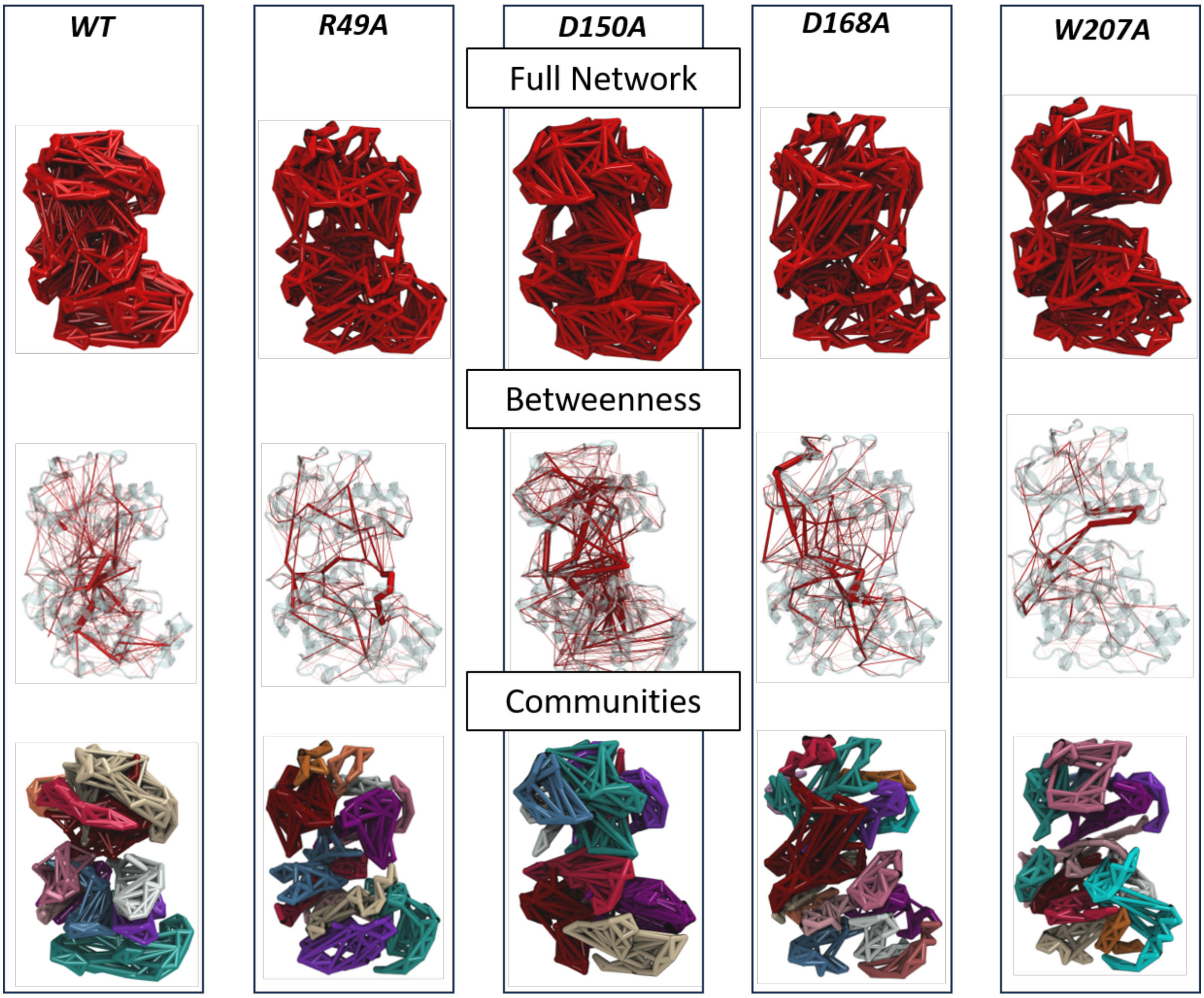

Supplementary Figure 7: Network analysis by Dynetan, mainly betweenness and communities of the mutants. The top shows the entire network, while the center depicts betweenness, and the bottom shows the clusters found in each of the mutants.

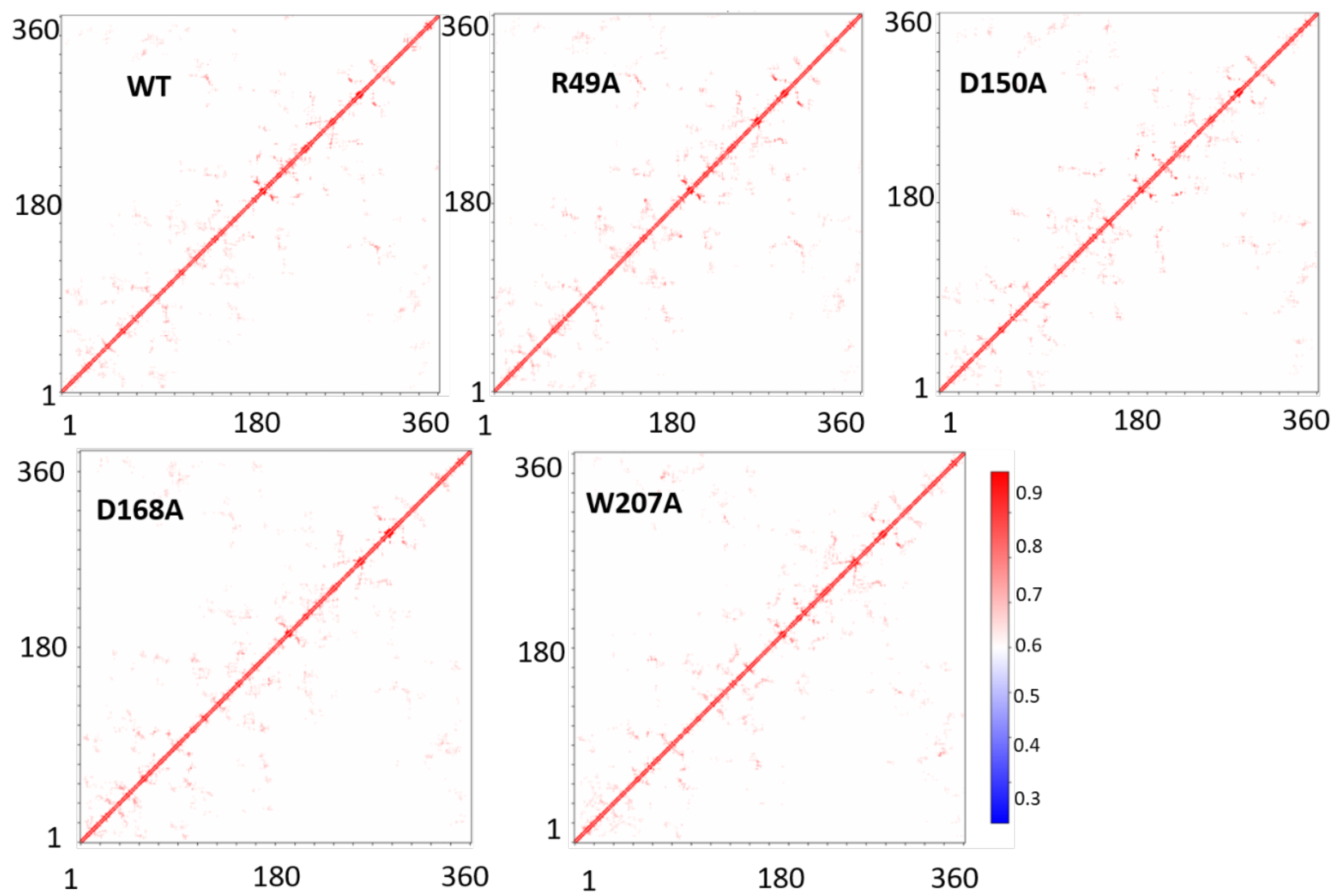

Supplementary Figure 8: Total correlation map of the WT and the mutants, obtained as generalised correlation coefficients through mutual information.

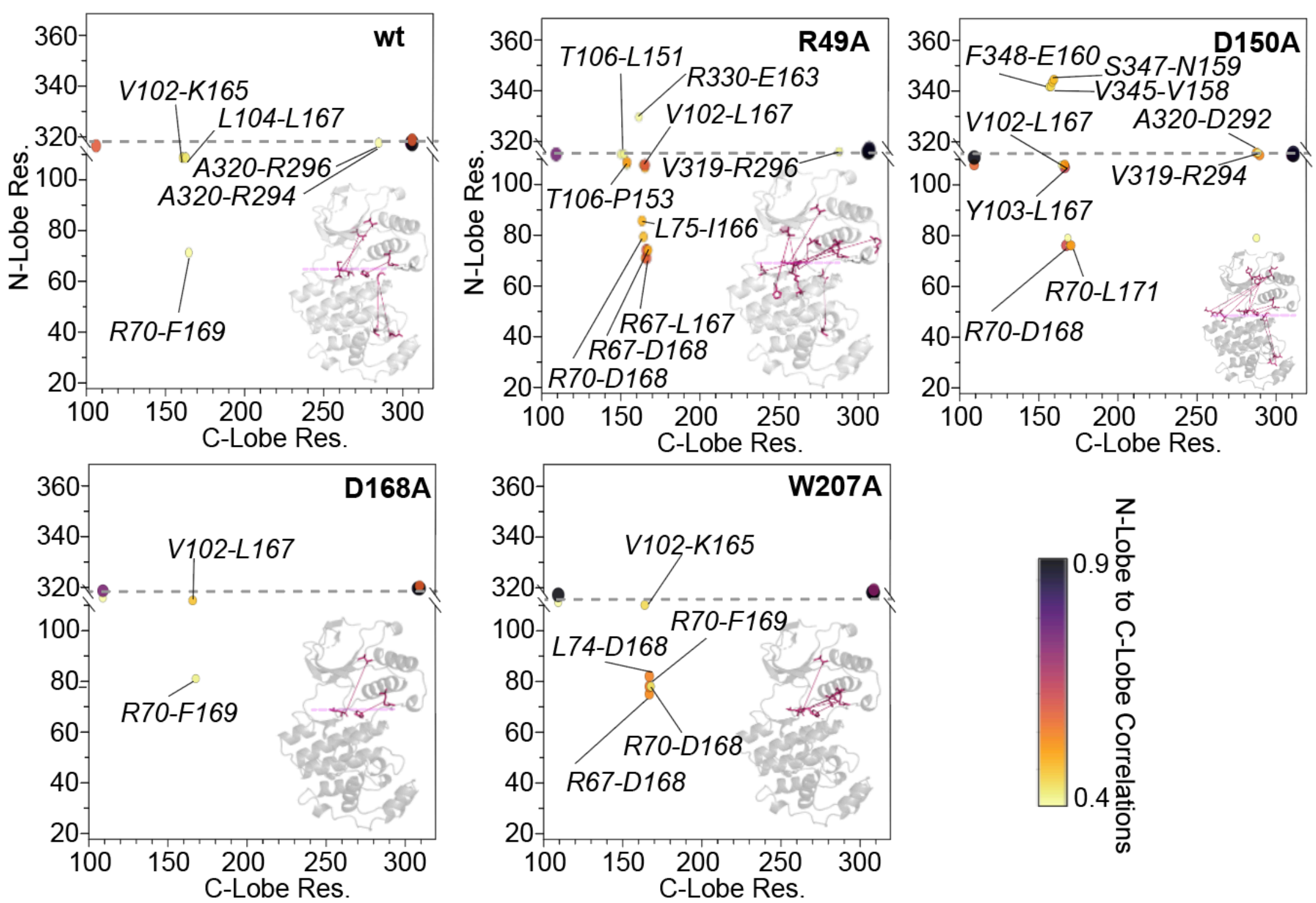

Supplementary Figure 9: N-C lobe correlations of the mutants using the correlation plots shown in Fig. S8 but focusing on the inter-domain regions. The correlation pairs are stated below in Table S3.

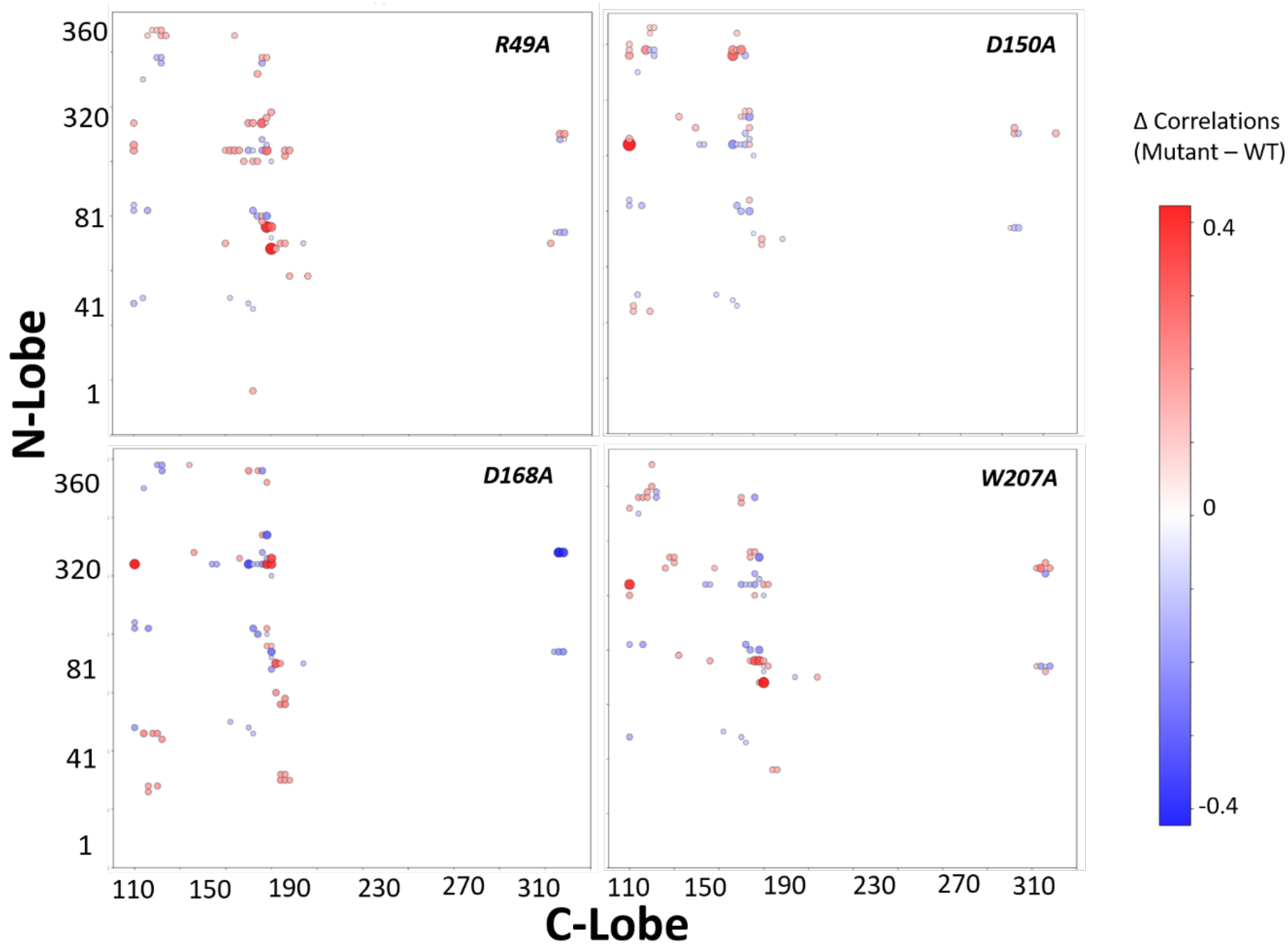

Supplementary Figure 10: Differences (mutant – WT) in the correlations from the N to C lobe for each of the mutants.

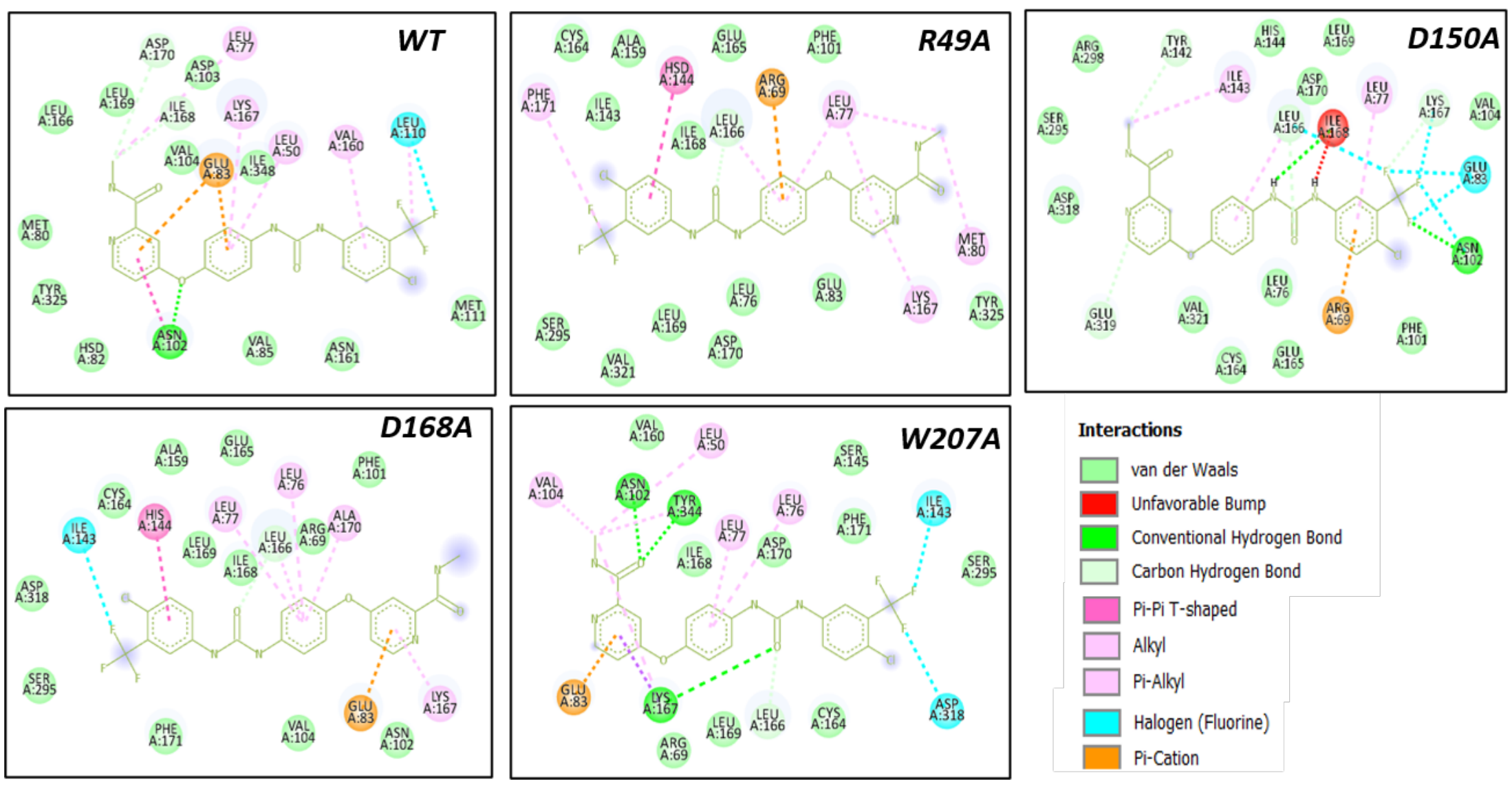

Supplementary Figure 11: Interacting residues when ligand sorafenib is docked with the WT protein and the mutants as seen through Discovery studio[13]. Note that the numbering of interacting residues provided by Discovery studio in this figure is shifted by +2 from the normal convention.

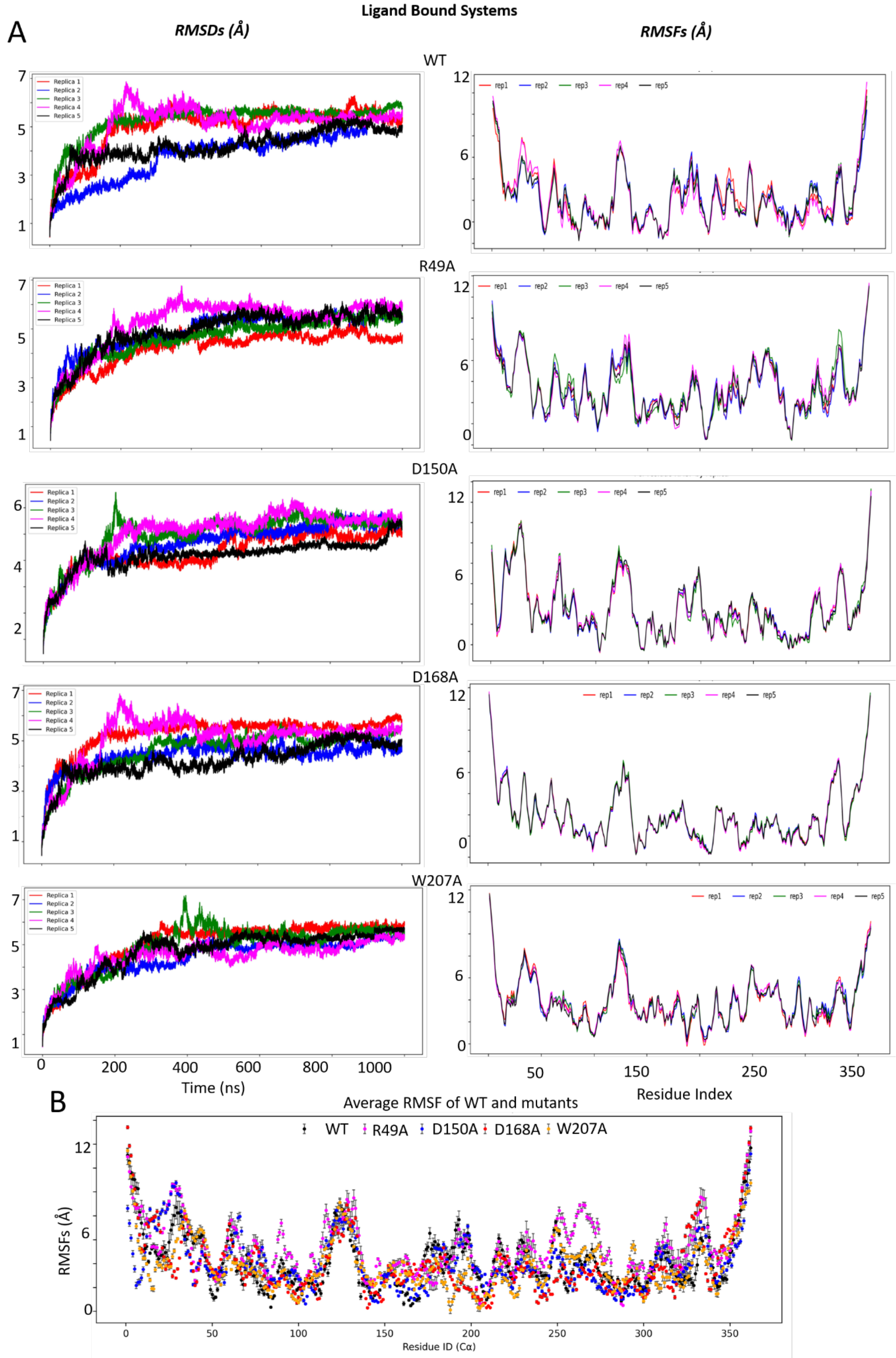

Supplementary Figure 12: A) RMSDs and RMSFs of five replicas per ligand-bound mutant. The last 200 ns each were used for network and correlation analysis. **B)** Mean and standard deviation of the RMSFs of the ligand-bound ensembles compared to each other.

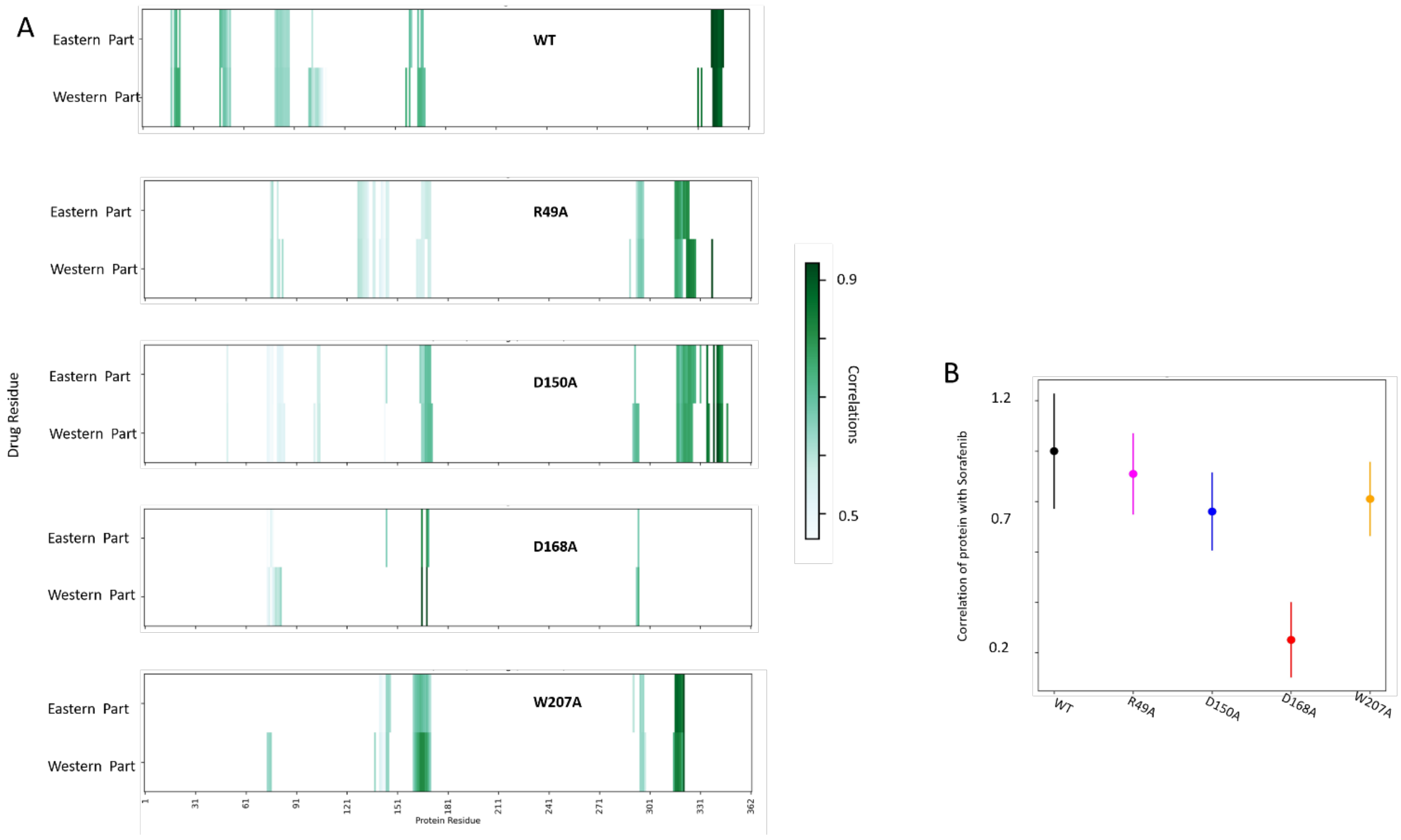

Supplementary Figure 13: Correlations of the ligand with the entire protein for WT and mutants. **A)** Relative correlations between the protein and the ligand (divided into two groups, the eastern and western part), resolved by protein residue. **B)** Total correlation of the ligand sorafenib with the mutants and the WT (the latter being normalized to 1).

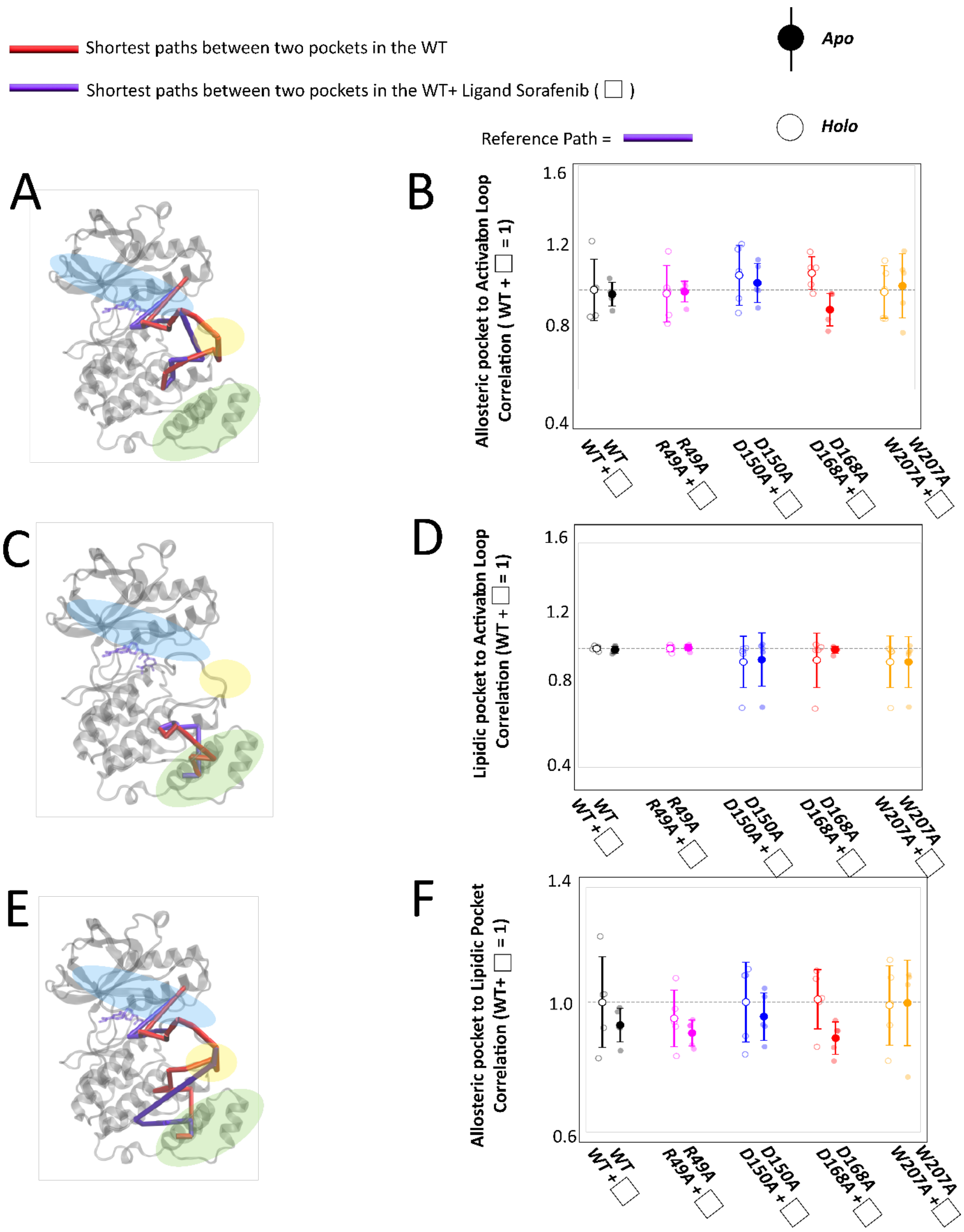

Supplementary Figure 14: A) Structural depiction of the communication pathway from the allosteric pocket to the activation loop in WT p38α. The apo WT shortest pathway is shown in red, and the sorafenib-bound WT pathway is shown in purple. **B**) Cumulative correlations along the allosteric-pocket-to-activation-loop pathway, calculated using the sorafenib-bound WT pathway as the reference. For more concise x-axis labels in panels B, D, and F, the ligand (sorafenib) is always denoted as a hollow square. **C)** Structural comparison of the lipid-pocket-to-activation-loop pathways in apo WT (red) and sorafenib-bound WT (purple). **D**) Cumulative correlations along the lipid-pocket-to-activation-loop pathway, calculated using the sorafenib-bound WT pathway as the reference. **E)** Structural comparison of the allosteric-pocket-to-lipid-pocket pathways in apo WT (red) and sorafenib-bound WT (purple). **F**) Cumulative correlations along the allosteric-pocket-to-lipid-pocket pathway, calculated using the sorafenib-bound WT pathway as the reference. In panels **B**, **D**, and **F**, filled and open symbols represent apo and sorafenib-bound systems, respectively. Large symbols indicate mean values, error bars represent ± SD, and smaller pale symbols represent individual trajectory values. Data were obtained from n=5 independently initiated 1 μs MD trajectories per system, with the final 200 ns of each trajectory used for analysis.

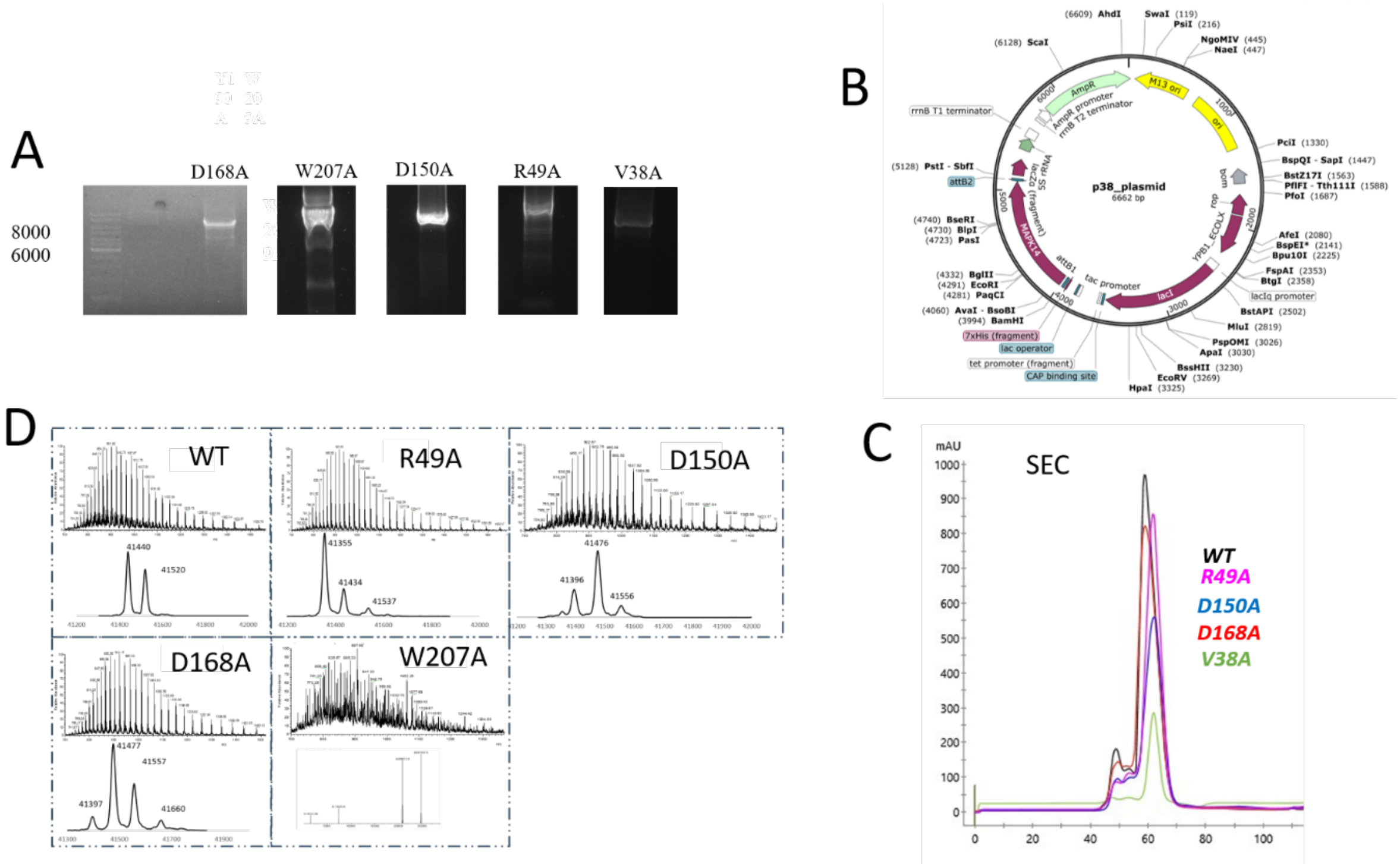

Supplementary Figure 15: **A**) Mutant DNA as seen through agarose gel electrophoresis. **B**) Plasmid of the entire p38α obtained through nanopore sequencing. **C**) SEC of the pure triple labelled (DCN) protein (WT and the mutants). **D**) Mass spectrometry results of the mutants and the WT, W209A shows a mix of different species.

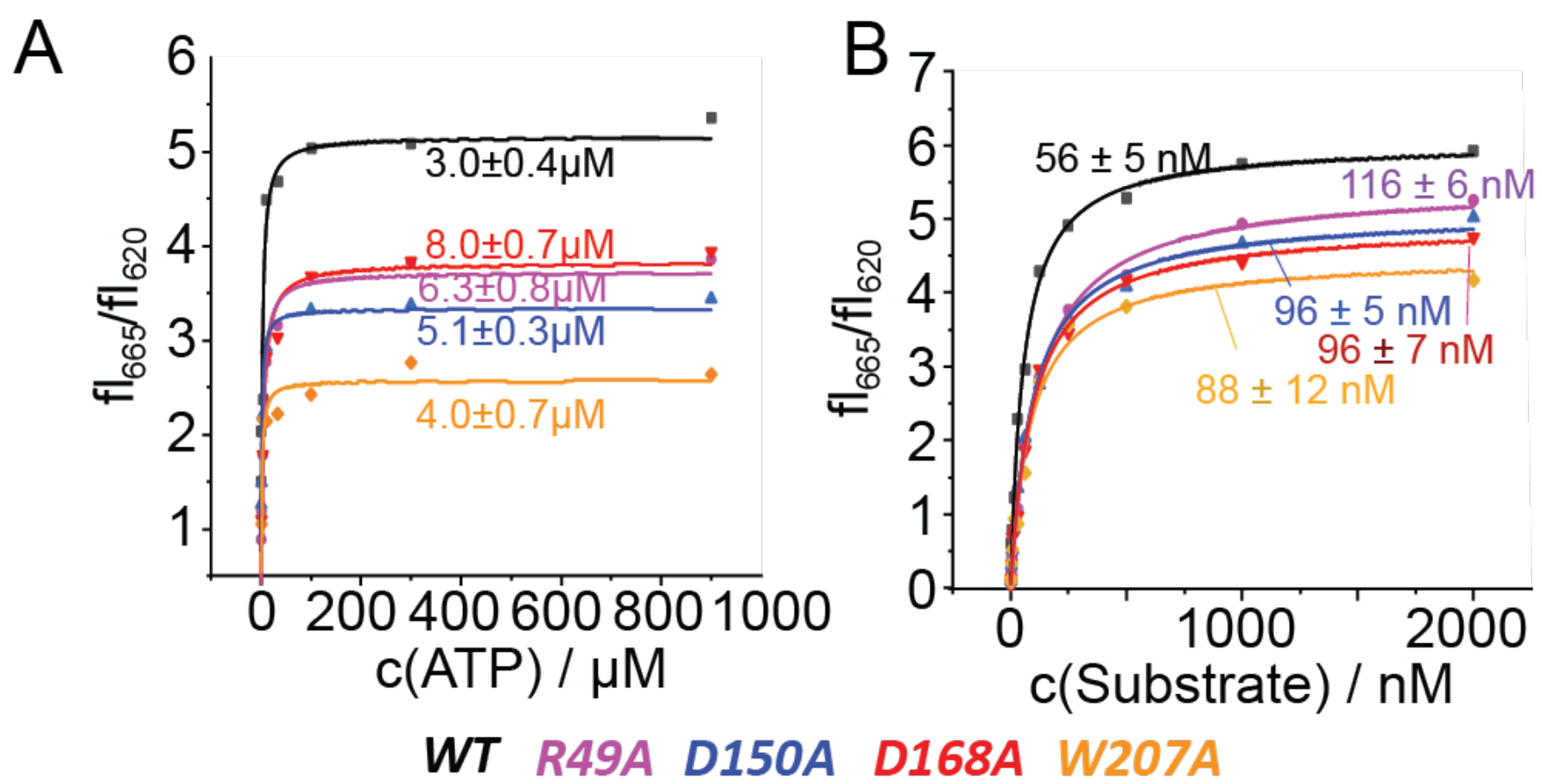

Supplementary Figure 16: HTRF responses for ATP and substrate titrations. **A)** ATP-dependent responses were fitted using the Michaelis–Menten equation. **B)** Substrate-dependent responses were fitted using the Hill equation. One activated protein preparation was used per construct, and each concentration was measured in four technical replicate wells. Data points represent the mean of the four wells.

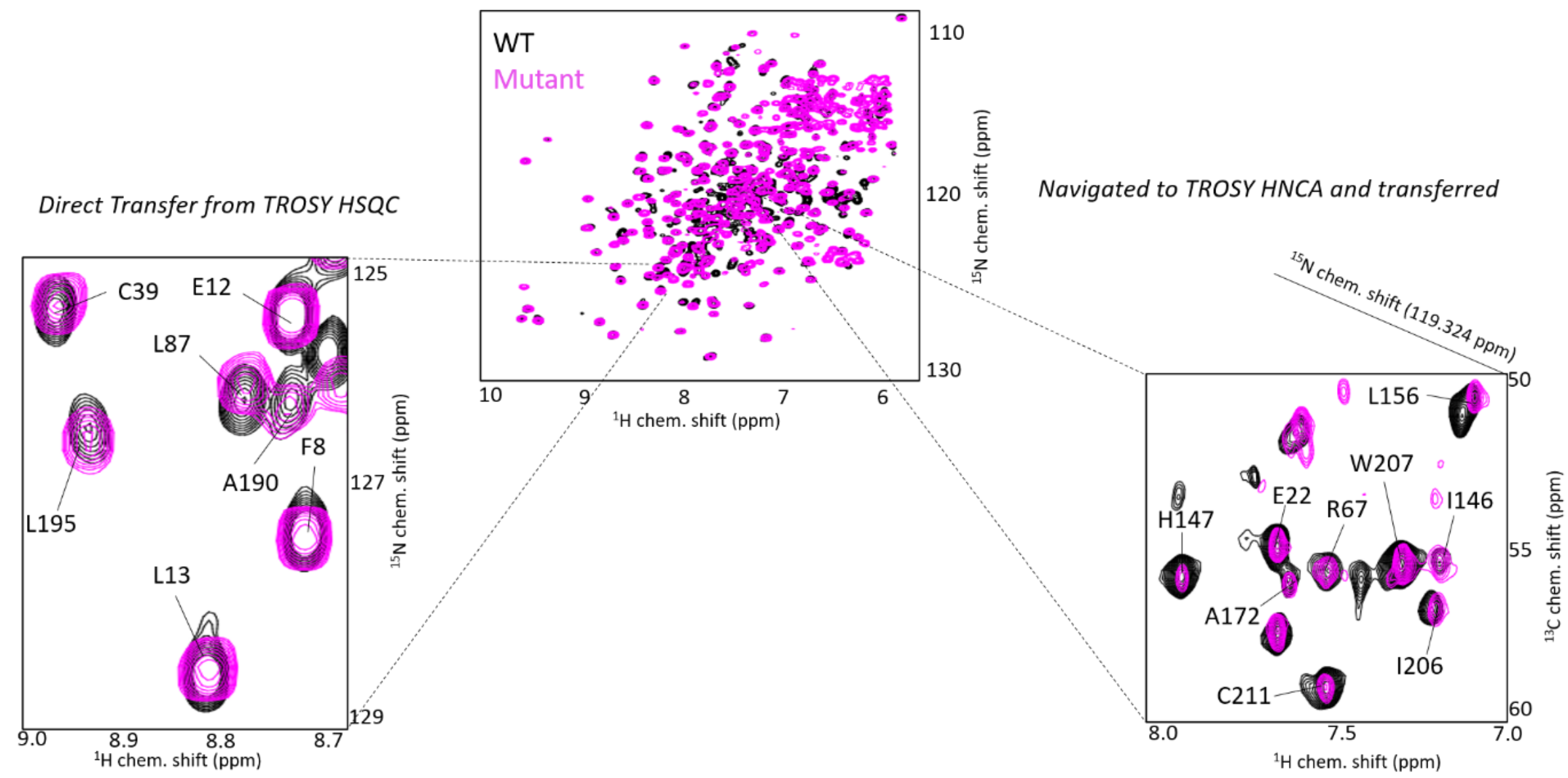

Supplementary Figure 17: Transfer of assignments using TROSY HSQC and HNCA spectra of the WT. (Chemical shifts of the WT are also available in the BMRB under accession code 17471.)

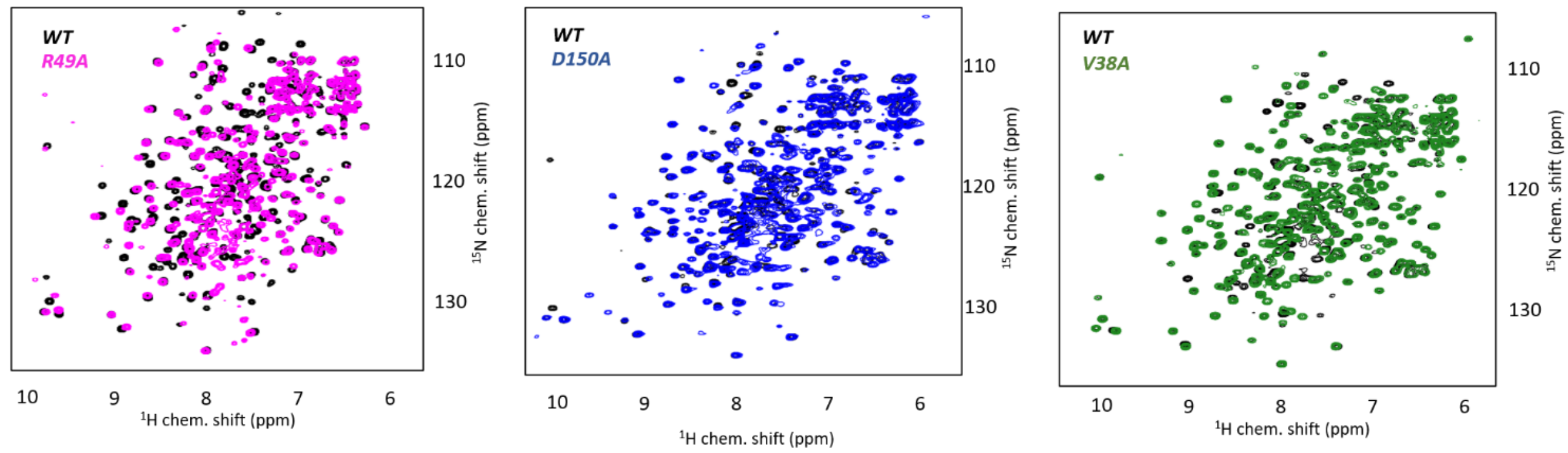

Supplementary Figure 18: A) TROSY-HSQC overlay of the mutants R49A, D150A, and negative control V38A with the WT.

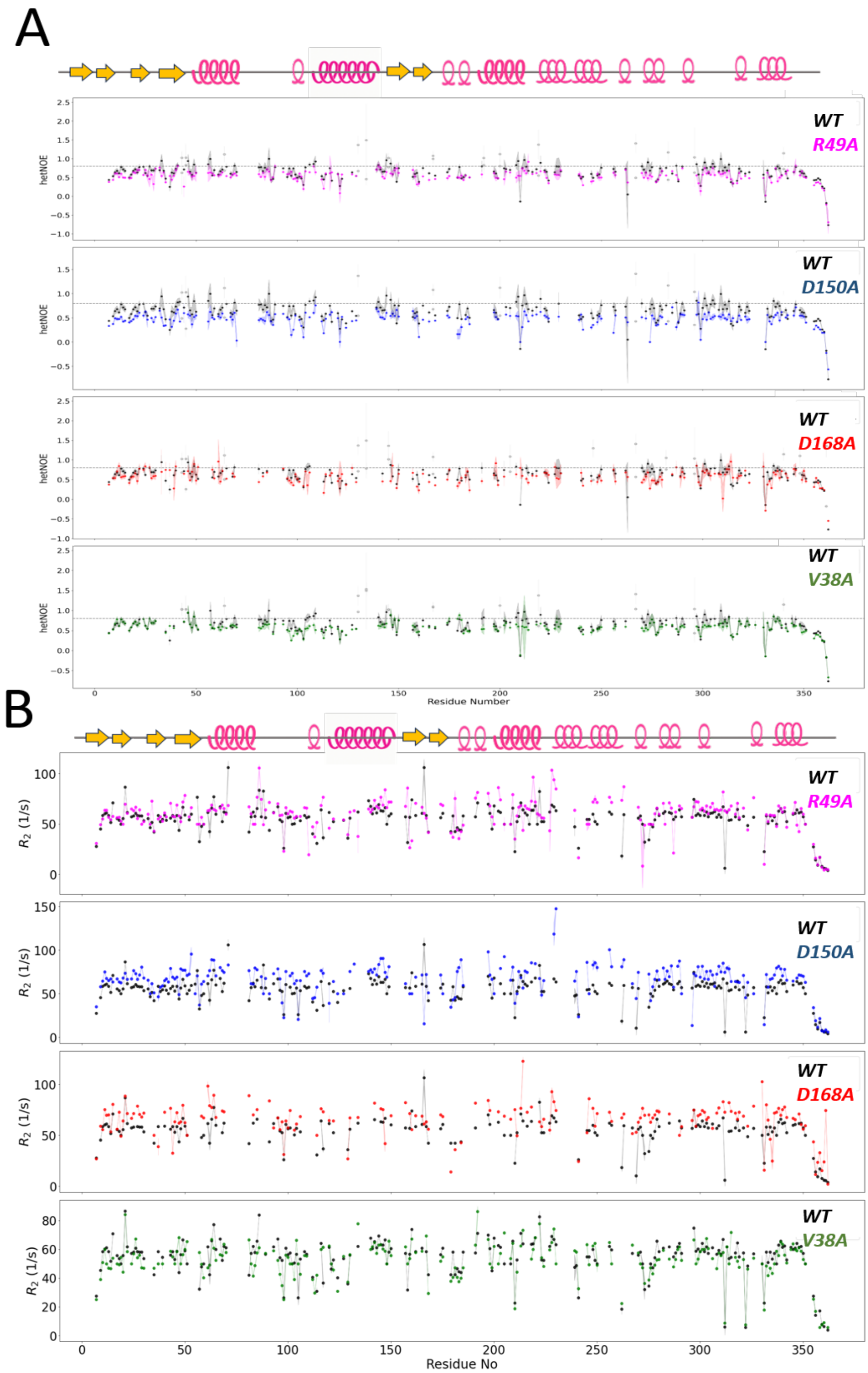

Supplementary Figure 19: A) Residue-wise hetNOE representation and comparison of the values (WT vs. mutants), including the negative control V38A. Note that the molecular weight of the protein, in conjunction with a relatively low temperature, incurred a suboptimal signal-to-noise ratio for the insensitive hetNOE experiment. hetNOE values of larger than ~0.9 are usually not observed and have to be judged with caution. Values above 1 are hence marked out in gray. These (usually poorly constrained) outliers must be excluded upon any discussions of the hetNOE dataset. **B)** Residue-wise R_2 representation and comparison of the values (WT vs. mutants), including the negative control V38A.

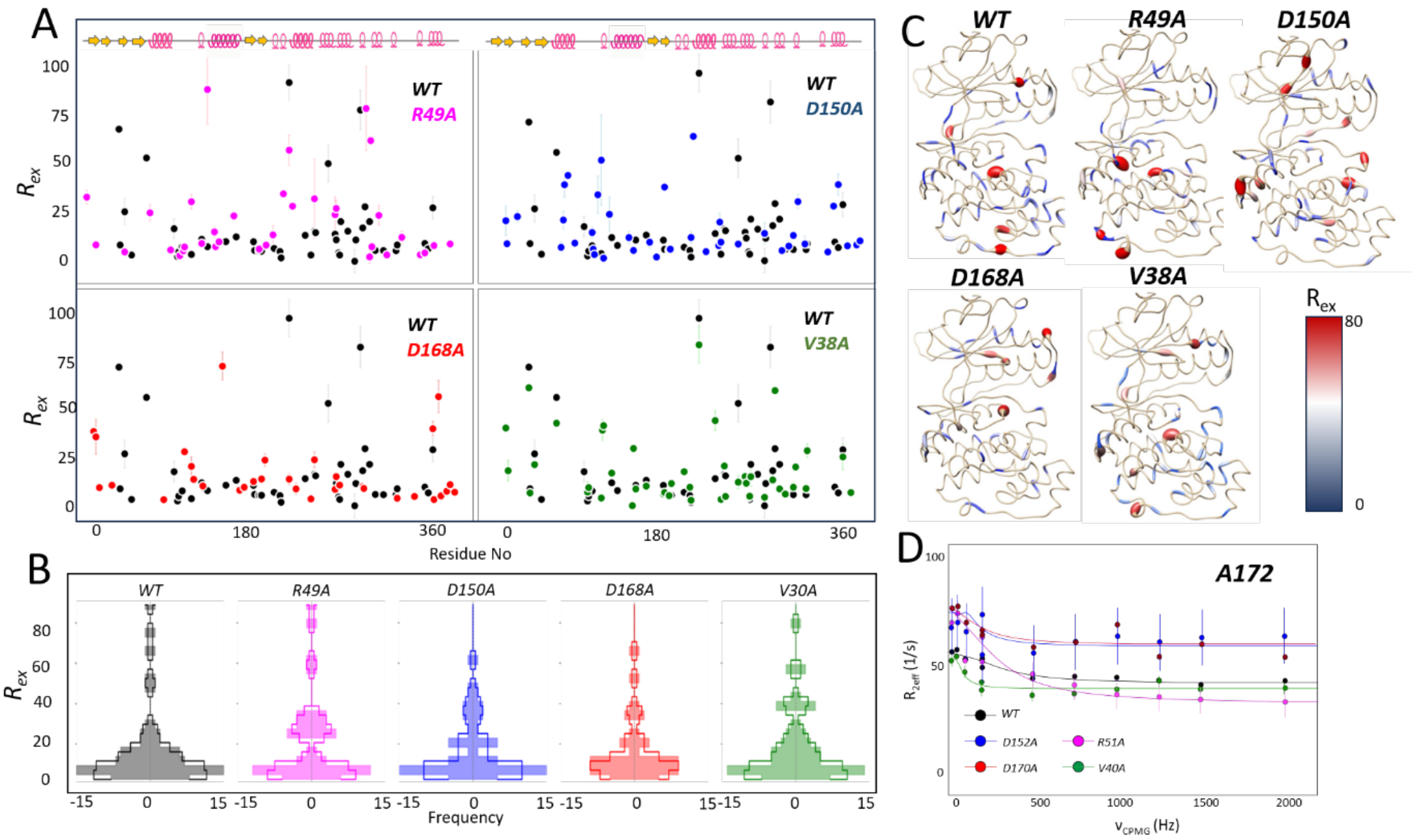

Supplementary Figure S20: Microsecond-to-millisecond conformational-exchange contributions in WT and mutant p38α. **A**) Residue-specific R_{ex} values derived from CPMG relaxation-dispersion measurements for WT and the indicated variants R49A, D150A, D168A, and V38A. Points represent fitted R_{ex} values, and error bars represent Monte Carlo-derived fitting uncertainties calculated by NESSY. **B**) Frequency distributions of residue-specific R_{ex} values. The numbers of residues included were n = 46 for WT, n = 37 for R49A, n = 40 for D150A, n = 33 for D168A, and n = 45 for V38A. Histogram bars indicate the number of residues within each R_{ex} interval. The accompanying box plots extend from the 25th to the 75th percentiles, with the centre line indicating the median. Whiskers extend to the most extreme values within 1.5 times the interquartile range, and values outside the whiskers are shown individually as outliers. The minimum and maximum correspond to the lowest and highest plotted observations, including outliers. **C**) Structural mapping of residue-specific R_{ex} values for WT and the indicated variants. **D**) Representative CPMG relaxation-dispersion profiles for residue A172. Points represent fitted $R_{2,eff}$ values, error bars represent Monte Carlo-derived fitting uncertainties calculated by NESSY, and solid lines represent the best-fit relaxation-dispersion curves. Data were obtained from n = 1 independently expressed and purified protein sample per construct.

A **WT**

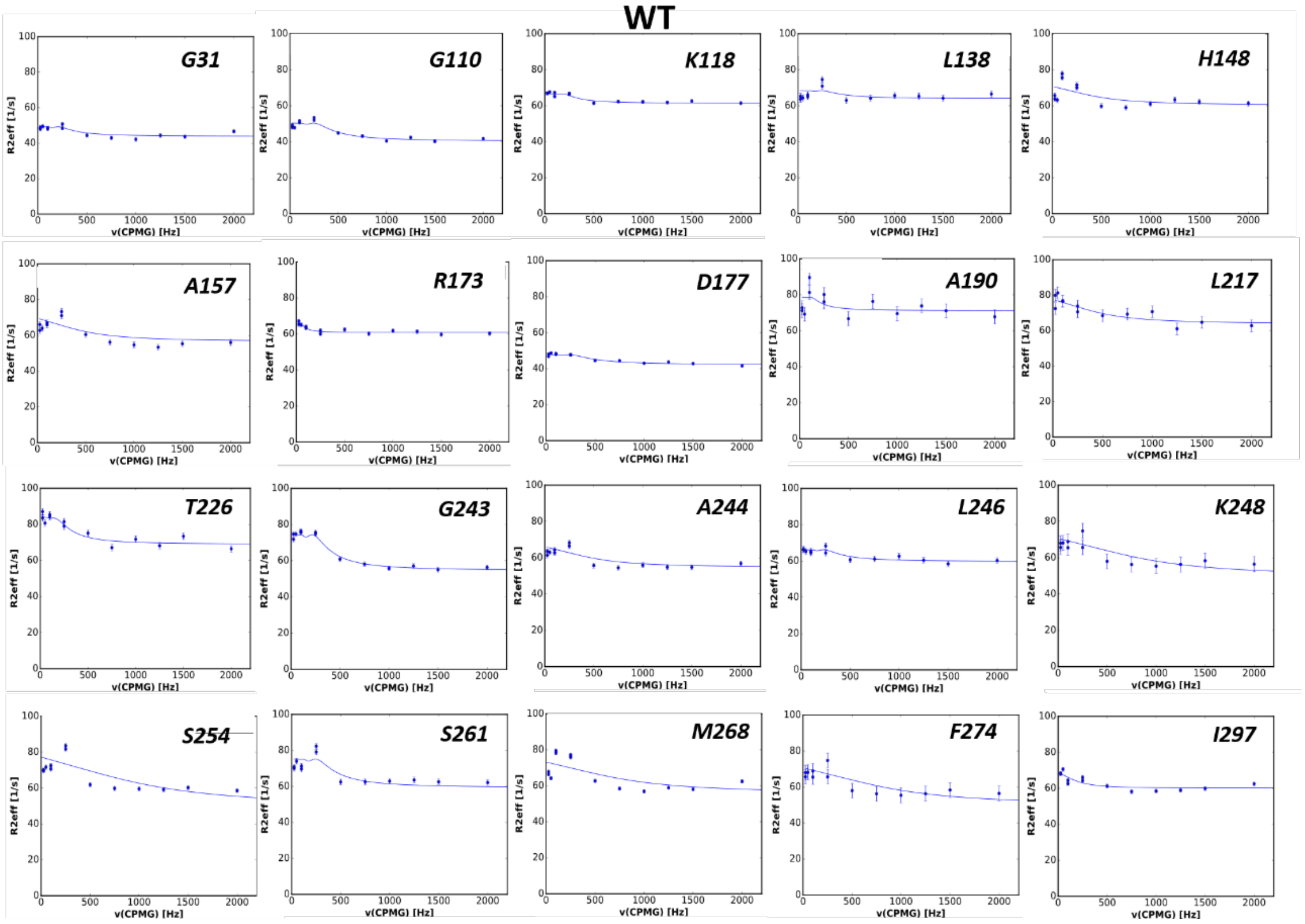
G31
G110
K118
L138
H148
A157
R173
D177
A190
L217
T226
G243
A244
L246
K248
S254
S261
M268
F274
I297
R2eff [1/s]
v(CPMG) [Hz]

B **R49A**

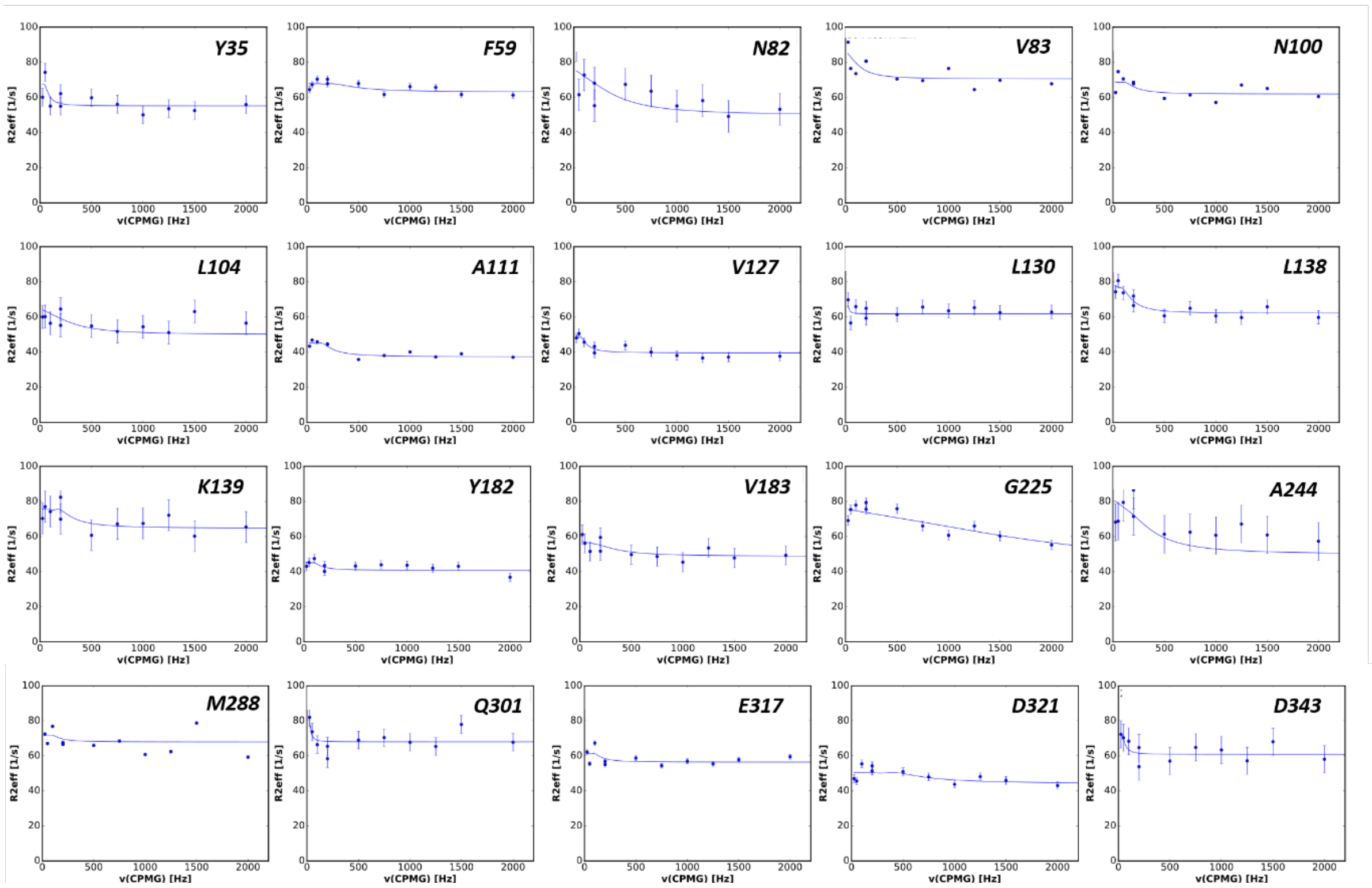
Y35
F59
N82
V83
N100
L104
A111
V127
L130
L138
K139
Y182
V183
G225
A244
M288
Q301
E317
D321
D343
R2eff [1/s]
v(CPMG) [Hz]

C **D150A**

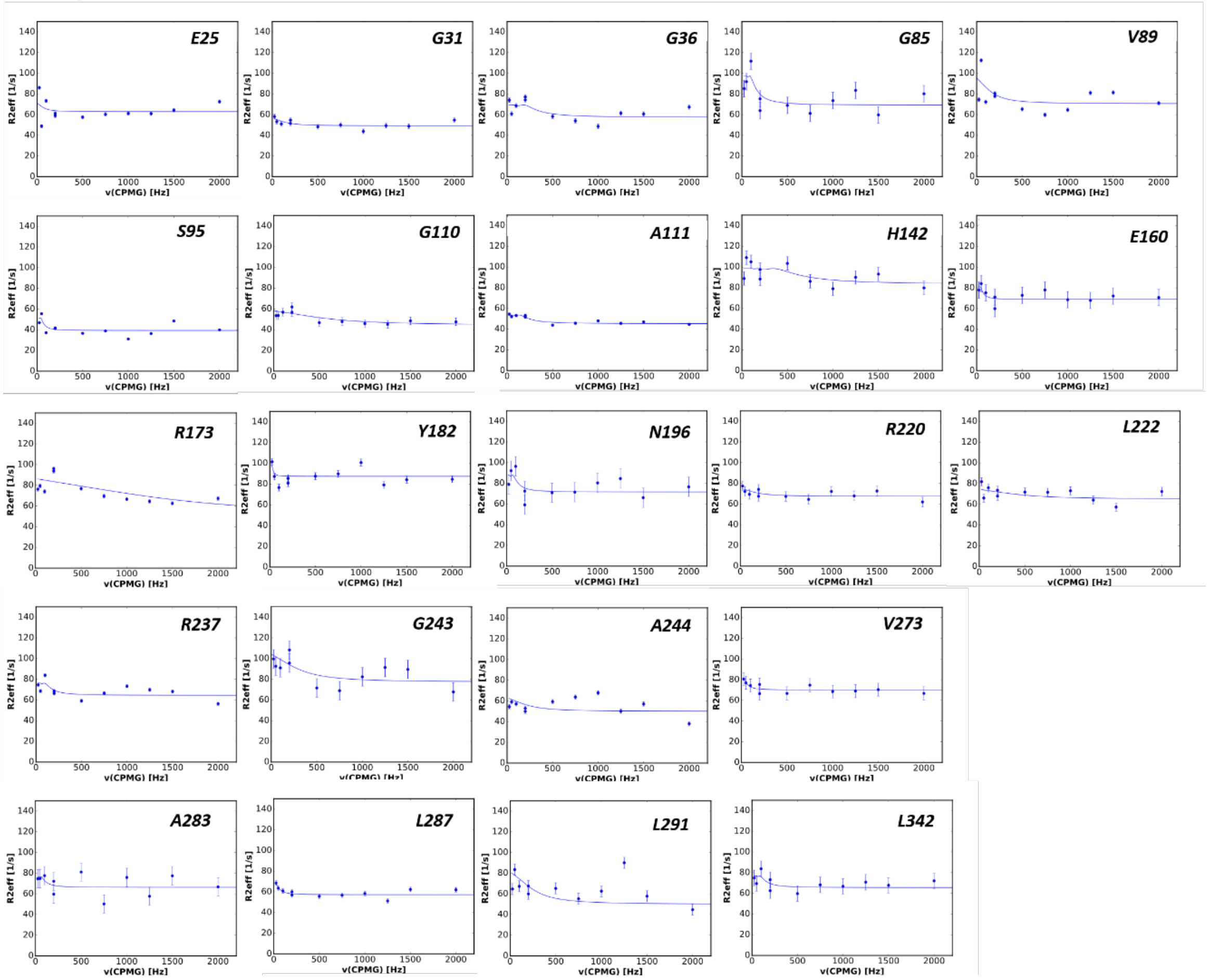

D **D168A**

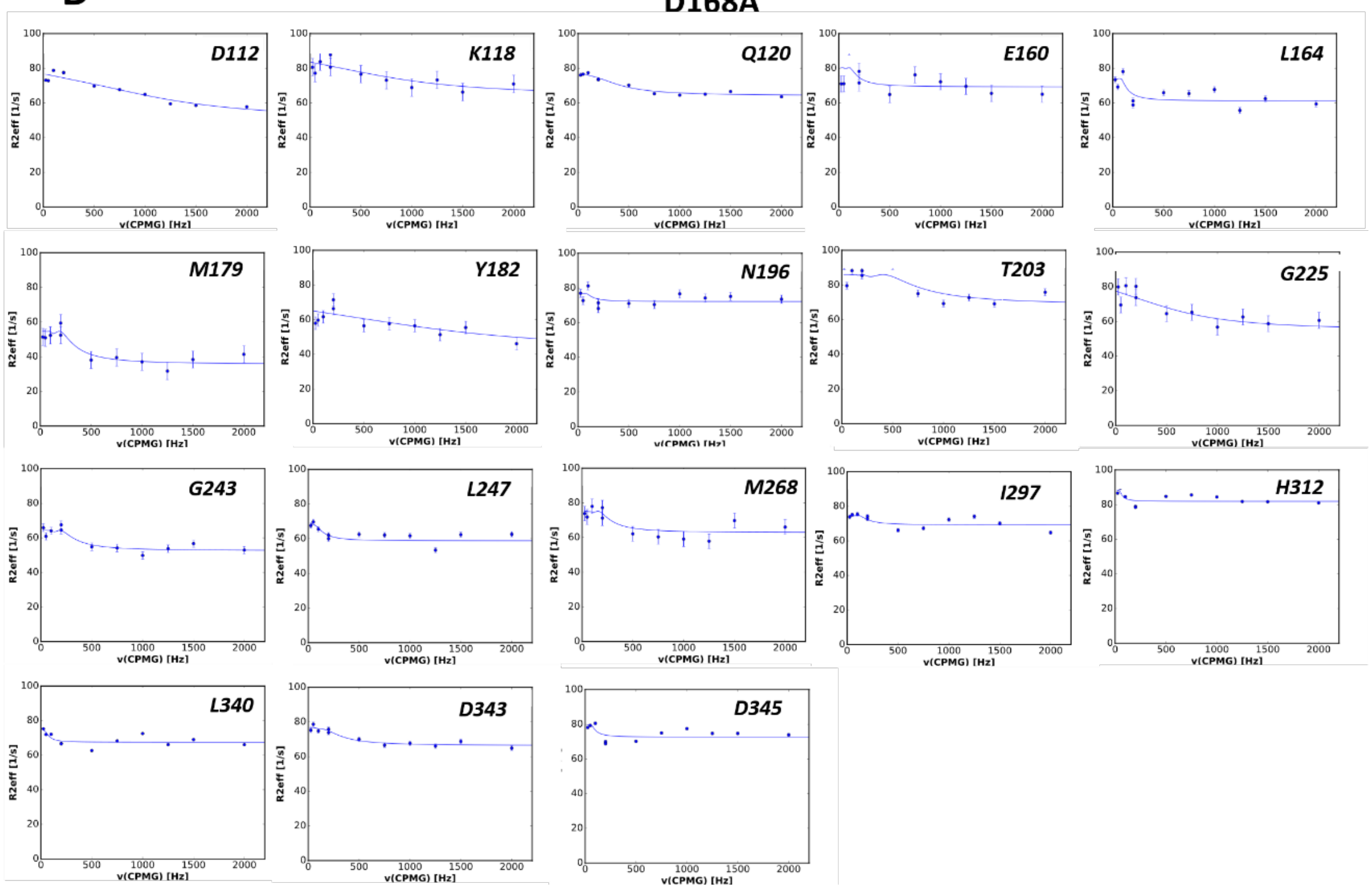

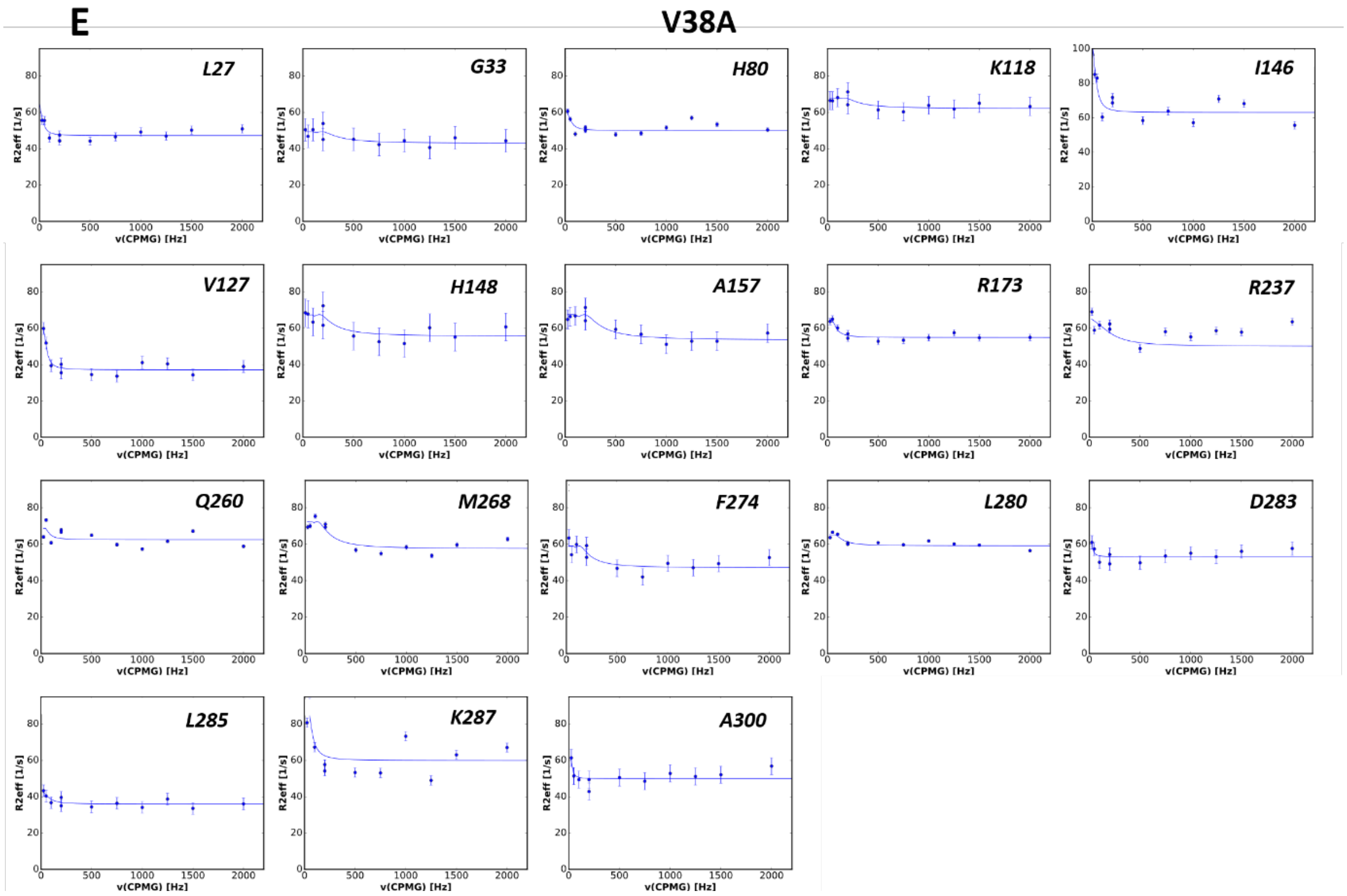

Supplementary Figure S21: Representative CPMG relaxation-dispersion profiles for WT and mutant p38α. Representative residue-specific profiles are shown for **A**) WT, **B**) R49A, **C**) D150A, **D**) D168A, and **E)** V38A. Points represent fitted $R_{2,eff}$ values at the indicated CPMG frequencies, error bars represent Monte Carlo-derived fitting uncertainties calculated by NESSY, and solid lines represent the best-fit relaxation-dispersion curves. Data were obtained from one independently expressed and purified protein sample per construct.

Supplementary tables

Supplementary Table 1: Summary of Molecular Dynamics system parameters.

**System**	**Box** (Å)	**Total No. of Atoms**	**Salt Pairs (NaCl)**	**Salt Concentration** (M)	**No. of Water Molecules**
Wildtype apo	63.65 * 100.91* 84.81	66,679	56	0.151	19,896
R49A apo	79.60* 102.97 * 81.84	68,577	58	0.146	20,883
D150A apo	79.59 * 102.97* 81.84	68,593	59	0.146	20,885
D168A apo	79.85 * 102.87 * 81.35	68,566	59	0.147	20,678
W207A apo	79.61* 102.99 * 81.85	68,576	59	0.146	20,883
Wildtype holo	106.46 * 70.03 * 90.98	69,469	60	0.147	21,159

R49A holo	102.93 * 85.05 * 71.93	64,445	55	0.145	19,492
D150A holo	101.80 * 84.49 * 71.93	63,037	54	0.145	19,020
D168A holo	100.66 * 85.60 * 71.41	62,995	54	0.146	19,006
W207A holo	102.50 * 85.11 * 72.13	64,201	55	0.145	19,411
3HEG (crystal structure with Sorafenib)	96.32 * 76.65 * 67.13	50,932	42	0.140	14,979
3GCS (crystal structure with Sorafenib and β-OG)	94.64 * 68.08 * 74.74	49,421	41	0.141	14,489

Supplementary Table 2: Residues showing highest betweenness centrality for the wildtype and mutants (with decreasing values from top to bottom in each of the columns).

**WT**	**R49A**	**D150A**	**D168A**	**W207A**
W207, I212	L135, N155	K165, D343	I131, D343	R14, I52
R136, I141	R149, T185	V105, L156	I131, L135	E141, L166
R136, T203	N155, N159	D205, S208	G210, L236	L205, V236
D150, D168	N14, E160	S154, R173	L135, G210	R139, E141
P29, R49	V117, I134	S154, S208	V102, I142	R135, M165
R136, W207	N14, N159	Y140, M288	I134, G137	I52, E66
K66, I141	I116, V158	K338, D343	N100, V102	E141, T167
W205, I210	I134, G137	H142, L280	G47, V102	G145, G170
Y140, D205	K66, K165	H142, L156	L236, S254	E274, T293
Y188, T203	G137, V273	H80, C162	C39, G47	E48, I102

Supplementary Table 3: Correlation Values Between N-lobe to C-Lobe.

**WT**			**R49A**			**D150A**			**D168A**			**W207A**		
N-lobe res.	C-lobe res.	Corr. val.	N-lobe res.	C-lobe res.	Corr. val.	N-lobe res.	C-lobe res.	Corr. val.	N-lobe res.	C-lobe res.	Corr. val.	N-lobe res.	C-lobe res.	Corr. val.
319	316	0.96	319	316	0.96	319	316	0.96	317	316	0.96	317	316	0.95
319	315	0.75	319	315	0.94	106	108	0.95	317	315	0.93	106	108	0.94

106	108	0.55	320	316	0.93	320	316	0.91	106	108	0.75	317	315	0.93
320	316	0.55	106	108	0.76	319	315	0.75	318	316	0.55	318	316	0.72
102	165	0.36	70	168	0.58	102	167	0.61	102	167	0.41	70	168	0.53
102	167	0.35	102	167	0.57	70	168	0.59	70	169	0.35	74	168	0.52
70	169	0.31	67	168	0.57	103	108	0.54	103	108	0.32	67	168	0.51
319	294	0.31	103	155	0.46	319	294	0.48	317	316	0.96	102	165	0.40
319	316	0.96	70	169	0.43	70	171	0.46	317	315	0.93	70	169	0.38
319	315	0.75	81	165	0.42	103	167	0.46	106	108	0.75	103	108	0.35
106	108	0.55	101	167	0.41	320	292	0.46	318	316	0.55	317	316	0.95
320	316	0.55	102	166	0.41	348	160	0.40	102	167	0.41	106	108	0.94
102	165	0.36	67	167	0.41	347	159	0.39	70	169	0.35	317	315	0.93
102	167	0.35	75	166	0.41	345	158	0.37	103	108	0.32	318	316	0.72
70	169	0.31	319	296	0.33	345	157	0.35				70	168	0.53
319	294	0.31	106	153	0.32	318	293	0.31				74	168	0.52
			106	151	0.32	73	292	0.31				67	168	0.51
			330	163	0.31	73	169	0.30				102	165	0.40
			102	155	0.31	70	170	0.30				70	169	0.38
			319	316	0.96	103	165	0.30				103	108	0.35
			319	315	0.94	319	316	0.96						
			320	316	0.93	106	108	0.95						
			106	108	0.76	320	316	0.91						
			70	168	0.58	319	315	0.75						
			102	167	0.57	102	167	0.61						
			67	168	0.57	70	168	0.59						
			103	155	0.46	103	108	0.54						
			70	169	0.43	319	294	0.48						
			81	165	0.42	70	171	0.46						
						103	167	0.46						

Supplementary Table 4: B-factors of key network residues, derived from PDB 3HEG.

**Residue**	**B-factor** (Å^2)
PRO29	0.434
ARG49	0.509
LYS66	0.206
ILE141	0.199
ASP150	0.342
ASP168	0.497
TYR188	0.111
THR203	0.207
TRP207	0.056
ILE212	0.107
ILE235	0.098

Supplementary Table 5: Pathways to and from different pockets.

Apo Pathway (red)	Allosteric Pocket to Activation Loop	{ K66 R67 }<br>{ R67 L167 }<br>{ L167 D168 }<br>{ D168 G170 }<br>{ G170 A172 }<br>{ A172 R173 }<br>{ R173 T175 }<br>{ T175 Y182 }<br>{ Y182 V183 }<br>{ V183 A184 }<br>{ A184 Y188 }
	Lipid Pocket to Activation Loop	{ R186 W187 }<br>{ W187 Y188 }<br>{ Y188 R189 }<br>{ R189 D227 }<br>{ D227 I229 }<br>{ I229 Q231 }<br>{ Q231 K233 }<br>{ K233 I235}
	Allosteric Pocket to lipid Pocket	{ K66 R67 }<br>{ R67 L167 }<br>{ L167 D168 }<br>{ D168 G170 }<br>{ G170 A172 }<br>{ A172 R173 }<br>{ R173 T175 }<br>{ T175 A176 }<br>{ A176 G178 }<br>{ G178 M179 }<br>{ M179 T221 }<br>{ T221 L222 }<br>{ L222 P224 }<br>{ P224 L232 }<br>{ L232 I235 }
Holo Pathway (purple)	Allosteric Pocket to Activation Loop	{ K66 D168 }<br>{ D168 F169 }<br>{ F169 L171 }<br>{ L171 R173 }<br>{ R173 T175 }<br>{ T175 A176 }<br>{ A176 G178 }<br>{ G178 T180 }<br>{ T180 G181 }<br>{ G181 V183 }<br>{ V183 T185 }<br>{ T185 Y188 }
	Lipid Pocket to Activation Loop	{ Y188 R189 }<br>{ R189 Y188 }<br>{ Y188 T221 }<br>{ T221 K233 }<br>{ K233 I235 }
	Allosteric Pocket to lipid Pocket	{ K66 D168 }<br>{ D168 F169 }

		{ F169 L171 }<br>{ L171 R173 }<br>{ R173 T175 }<br>{ T175 A176 }<br>{ A176 G178 }<br>{ G178 T180 }<br>{ T180 G181 }<br>{ G181 V183 }<br>{ V183 A184 }<br>{ A184 R186 }<br>{ R186 Y188 }<br>{ Y188 T221 }<br>{ T221 K233 }<br>{ K233 I235 }

Supplementary Table 6: Statistical analyses were based on n = 5 independently initiated 1-µs MD trajectories per system, using the final 200 ns of each trajectory. The observed difference in mean normalized correlation was used as the test statistic. WT and each mutant were compared using a two-sided non-parametric permutation test with 10,000 label permutations. No adjustment for multiple comparisons was applied, and the reported p values are unadjusted. Hedges' g is reported as the standardized effect size. The table provides 95% bootstrap confidence intervals for the mean differences. Degrees of freedom are not applicable to the permutation test.

Apo protein pathway							
**Pathway**	**Comparison**	**Variant mean norm**	**Mean diff**	**95% CI low**	**95% CI high**	**Hedges' g**	**p-value**
**Allosteric → Activation**	WT vs 49	0.95	-0.05	-0.1	0.01	-0.84	0.1862
	WT vs 150	0.95	-0.05	-0.11	0	-0.87	0.1664
	WT vs 168	0.93	-0.07	-0.14	0	-0.93	0.1414
	WT vs 207	0.88	-0.12	-0.2	-0.04	-1.44	0.0394
**Activation → Lipid**	WT vs 49	0.9	-0.1	-0.19	-0.02	-1.17	0.0701
	WT vs 150	0.93	-0.07	-0.14	0	-0.98	0.1582
	WT vs 168	0.88	-0.12	-0.21	-0.04	-1.39	0.0425
	WT vs 207	0.92	-0.08	-0.14	-0.01	-1.08	0.1273
**Allosteric → Lipid**	WT vs 49	0.9	-0.1	-0.16	-0.06	-2.1	0.0171
	WT vs 150	0.85	-0.15	-0.21	-0.11	-3.06	0.0026
	WT vs 168	0.84	-0.16	-0.21	-0.11	-3.03	0.0022
	WT vs 207	0.78	-0.22	-0.28	-0.18	-4.55	0.0005
Sorafenib bound protein pathway							
**Pathway**	**Comparison**	**Variant mean norm**	**Mean diff**	**95% CI low**	**95% CI high**	**Hedges' g**	**Bootsrap p**
**Allosteric → Activation**	LWT vs WT	0.98	-0.02	-0.14	0.08	-0.17	0.7758
	LWT vs L49	0.98	-0.02	-0.16	0.13	-0.11	0.8465
	LWT vs 49	0.99	-0.01	-0.13	0.09	-0.07	0.9056

	LWT vs L150	1.06	0.06	-0.09	0.2	0.44	0.4681
	LWT vs 150	1.03	0.03	-0.1	0.15	0.25	0.6795
	LWT vs L168	1.07	0.07	-0.05	0.18	0.62	0.3202
	LWT vs 168	0.91	-0.09	-0.22	0.02	-0.73	0.2472
	LWT vs L207	0.99	-0.01	-0.15	0.12	-0.07	0.9079
	LWT vs 207	1.02	0.02	-0.14	0.16	0.11	0.8481
**Activation → Lipid**	LWT vs WT	0.99	-0.01	-0.02	0.01	-0.39	0.5132
	LWT vs L49	1	0	-0.02	0.02	0	1
	LWT vs 49	1	0	-0.01	0.02	0.24	0.6847
	LWT vs L150	0.93	-0.07	-0.19	0	-0.67	0.3079
	LWT vs 150	0.94	-0.06	-0.19	0.02	-0.53	0.407
	LWT vs L168	0.94	-0.06	-0.19	0.01	-0.55	0.3925
	LWT vs 168	0.99	-0.01	-0.03	0.01	-0.38	0.5226
	LWT vs L207	0.93	-0.07	-0.2	0	-0.66	0.3135
	LWT vs 207	0.93	-0.07	-0.19	0	-0.68	0.3013
**Allosteric → Lipid**	LWT vs WT	0.93	-0.07	-0.19	0.04	-0.6	0.3397
	LWT vs L49	0.95	-0.05	-0.18	0.07	-0.38	0.5246
	LWT vs 49	0.91	-0.09	-0.21	0.01	-0.83	0.2093
	LWT vs L150	1	0	-0.15	0.13	0	0.9953
	LWT vs 150	0.96	-0.04	-0.17	0.07	-0.36	0.554
	LWT vs L168	1.01	0.01	-0.12	0.13	0.07	0.9076
	LWT vs 168	0.89	-0.11	-0.23	0	-0.95	0.1568
	LWT vs L207	0.99	-0.01	-0.16	0.13	-0.07	0.9113
	LWT vs 207	1	0	-0.16	0.13	-0.02	0.9789

Supplementary Table 7: Docking results of WT and mutant with Sorafenib at the allosteric pocket (in kcal/mol).

**WT**	**R49A**	**D150A**	**D168A**	**W207A**
-8.74	-3.69	-3.58	-6.56	-3.20
-7.74	-2.70	-3.35	-6.01	-0.35
-7.14		-2.72	-5.67	-0.35
-6.08		-1.70		-0.30
				-0.25

Supplementary Table 8: K_M, v_{max} and EC_{50} values. Note that v_{max} (needs to be read as relative values as the conversion between fluorescence and concentrations is unknown) is not reliable as the protein concentrations and degree of phosphorylation might be slightly different between samples.

Protein	WT	D168A	R49A	D150A	W207A	
K_M (µM)	3.0+/-0.4	8.0+/-0.7	6.3+/-0.8	5.1+/-0.3	4.0+/-0.7	*from ATP plot*
v_{max} (a.u.)	5.4+/-0.1	3.9+/-0.2	3.7+/-0.1	3.4+/-0.1	2.8+/-0.1	
R-sq	0.93	0.97	0.96	0.94	0.94	
$K_{0.5}$ /EC_{50} (nM)	56.34 ± 5.0	95.89 ± 4.7	116.24 ± 5.9	95.82 ± 6.5	88.44 ± 11.7	*from substrate plot*
v_{max} (a.u.)	6.03+/-0.3	5.47+/-0.5	5.09+/-0.6	5.98+/-0.4	5.75+/-0.3	
r^2	0.99	0.99	0.99	0.99	0.99	

Supplementary Table 9 : Mean CSP and their calculated error using Linewidth in ppm

Mutant	Mean CSP	Mean Linewidth error
D150	0.051054	0.069773
D168	0.034724	0.069684
R49	0.020492	0.06874
V38	0.003105	0.069105

Supplementary Table 10: Important regions with CSP > 0.12 for all mutants

Residue	Mutants	Region
31	D168A	P-loop / glycine-rich loop
50	R49A, D150A	Allosteric pocket / N-lobe pocket
69	R49A	Allosteric pocket / N-lobe pocket
82	R49A	Other / distal region
86	R49A, D150A	Other / distal region
95	D150A	Other / distal region
100	D150A	Other / distal region
122	D150A	Other / distal region
143	R49A, D150A	HRD / catalytic loop region
148	D150A	HRD / catalytic loop region
154	D150A	Other / distal region
157	D168A	Other / distal region
164	D168A	Other / distal region
177	D150A	Other / distal region
182	D150A	Activation loop / phosphorylation region
209	D150A	Other / distal region
210	D168A	Other / distal region
212	R49A	Other / distal region
228	D150A	Lipid pocket / MAP kinase insert

265	R49A	Lipid Pocket connect
267	D150A	Other / distal region
285	D150A, D168A	Other / distal region
311	R49A	Other / distal region
320	D150A	Other / distal region
359	D168A	Other / distal region

Supplementary Table 11: Important regions around the protein shown by all mutants, determined by difference in R_2

**Residue**	**Mutants**	**Region**	**Mean delta** (mutant – WT, Hz)
310	D150A, D168A, R49A	C-terminal docking / common docking region	64.23073
164	D150A, D168A, R49A	Other / distal region	-60.5253
260	D150A, D168A, R49A	Lipid pocket connector	55.59837
115	D150A, D168A, R49A	Other / distal region	37.86612
79	D150A, D168A, R49A	Other / distal region	35.68239
227	D150A, R49A	Lipid pocket connector	39.281
267	D150A, D168A	Lipid pocket connector	38.34436
273	D150A, D168A	Lipid pocket connector	36.94594
60	D150A, D168A	N-lobe regulatory / allosteric-pocket-adjacent region	35.57432
270	D168A, R49A	Lipid pocket connector	-7.73536
89	D168A, R49A	Other / distal region	33.83916
86	D150A, R49A	Other / distal region	-33.2876
228	D150A	Lipid pocket connector	84.3543
359	D168A	Other / distal region	68.40611
212	D168A	W207-adjacent substrate-binding groove / C-lobe region	58.71044
156	R49A	HRD/catalytic-loop-adjacent region	54.3454
208	D168A	W207-adjacent substrate-binding groove / C-lobe region	52.40458
328	D168A	Other / distal region	52.28157
182	D150A	Activation loop / phosphorylation region	45.21017
294	D150A	Other / distal region	-43.3047
203	D150A	W207-adjacent substrate-binding groove / C-lobe region	42.5236
158	R49A	Other / distal region	-40.7218
320	D150A	C-terminal docking / common docking region	39.67871

254	D150A	Lipid pocket / MAP kinase insert	38.59222
69	R49A	alphaC-helix region	-37.6729
108	R49A	ATP-site hinge region	-37.5516
271	D168A	Lipid pocket connector	37.33149
59	D168A	N-lobe regulatory / allosteric-pocket-adjacent region	36.69748
51	D150A	N-lobe regulatory / allosteric-pocket-adjacent region	35.7457
181	D150A	Activation loop / phosphorylation region	35.425
226	R49A	Lipid pocket connector	34.71639
285	R49A	Lipid pocket connector	-33.5478
247	D150A	Lipid pocket / MAP kinase insert	33.18465
179	R49A	Activation loop / phosphorylation region	32.99165
217	R49A	Other / distal region	32.57734
243	D150A	Lipid pocket / MAP kinase insert	31.54835
244	D168A	Lipid pocket / MAP kinase insert	30.91248
157	R49A	HRD/catalytic-loop-adjacent region	-30.2921
333	D168A	Other / distal region	-28.6189
312	D168A	C-terminal docking / common docking region	28.22658
177	D168A	Other / distal region	-27.9597
261	R49A	Other / distal region	27.84709
54	R49A	N-lobe regulatory / allosteric-pocket-adjacent region	-26.6474

Supplementary Table 12: Important regions around the protein shown by all mutants, determined by difference in hetNOE

**Residue**	**Mutants**	**Region**	**Mean delta** (mutant – WT)
178	D150A, D168A, R49A	Activation Loop	-0.42479
307	D150A, D168A, R49A	C-terminal docking / common docking region	-0.41298
261	D150A, D168A, R49A	Lipid pocket connector	0.398453
208	D168A, R49A	W207-adjacent substrate-binding groove / C-lobe region	0.809916
272	D150A, D168A	Lipid pocket connector	-0.54573
96	D150A, D168A	Other / distal region	-0.43552

31	D150A, R49A	P-loop / glycine-rich loop	-0.42107
207	D150A, R49A	W207-adjacent substrate-binding groove / C-lobe region	-0.39838
35	D168A, R49A	P-loop / glycine-rich loop	0.39117
84	D150A, R49A	Other / distal region	-0.38912
55	D150A, R49A	N-lobe regulatory / allosteric-pocket-adjacent region	-0.36746
142	D150A, R49A	Other / distal region	-0.3359
157	D168A, R49A	HRD/catalytic-loop-adjacent region	-0.30069
308	D168A	C-terminal docking / common docking region	-0.66113
68	D150A	alphaC-helix region	-0.61569
312	D168A	C-terminal docking / common docking region	0.581011
254	D150A	Lipid pocket / MAP kinase insert	-0.53586
285	D150A	Other / distal region	-0.50107
103	D168A	Other / distal region	-0.45686
42	R49A	Other / distal region	-0.45419
209	D150A	W207-adjacent substrate-binding groove / C-lobe region	-0.44567
54	D150A	N-lobe regulatory / allosteric-pocket-adjacent region	-0.42724
271	R49A	Lipid pocket connector	-0.42172
144	D168A	Other / distal region	-0.41166
40	D150A	P-loop / glycine-rich loop	-0.40812
95	D150A	Other / distal region	-0.39941
335	R49A	Other / distal region	-0.39584
280	D150A	Other / distal region	-0.38341
59	D168A	N-lobe regulatory / allosteric-pocket-adjacent region	0.381286
268	D168A	Lipid pocket connector	-0.36994
44	D150A	Other / distal region	-0.36641
27	R49A	Other / distal region	-0.36142
147	R49A	HRD/catalytic-loop-adjacent region	0.35499
303	D150A	C-terminal docking / common docking region	-0.35133
25	R49A	Other / distal region	-0.33643
37	D168A	P-loop / glycine-rich loop	-0.33586
189	D168A	Other / distal region	-0.33503
206	R49A	W207-adjacent substrate-binding groove / C-lobe region	-0.33385

79	D168A	Other / distal region	-0.33104
330	D168A	Other / distal region	-0.31373
196	D168A	Other / distal region	0.310641
47	R49A	N-lobe regulatory / allosteric-pocket-adjacent region	-0.30215
49	D168A	N-lobe regulatory / allosteric-pocket-adjacent region	0.30025
295	R49A	Other / distal region	-0.29854
