# Supplementary material for: Mapping of dynamic allostery within p38 alpha kinase via network analyses and NMR spectroscopy
Source: Nat Commun. 2026 Aug 22;17:10069. doi: 10.1038/s41467-026-76885-7 (PMC13594348; doi:10.1038/s41467-026-76885-7)
Supplement: Supplementary file 2 [file 41467_2026_76885_MOESM2_ESM.pdf]

nature portfolio

Corresponding author(s): Rasmus Linser, Rafael C. Bernardi

Last updated by author(s): 27.07.26

Reporting Summary

Nature Portfolio wishes to improve the reproducibility of the work that we publish. This form provides structure for consistency and transparency in reporting. For further information on Nature Portfolio policies, see our Editorial Policies and the Editorial Policy Checklist.

Statistics

For all statistical analyses, confirm that the following items are present in the figure legend, table legend, main text, or Methods section.

n/a	Confirmed	
☐	☒	The exact sample size (*n*) for each experimental group/condition, given as a discrete number and unit of measurement
☐	☒	A statement on whether measurements were taken from distinct samples or whether the same sample was measured repeatedly
☐	☒	The statistical test(s) used AND whether they are one- or two-sided<br>*Only common tests should be described solely by name; describe more complex techniques in the Methods section.*
☒	☐	A description of all covariates tested
☒	☐	A description of any assumptions or corrections, such as tests of normality and adjustment for multiple comparisons
☐	☒	A full description of the statistical parameters including central tendency (e.g. means) or other basic estimates (e.g. regression coefficient) AND variation (e.g. standard deviation) or associated estimates of uncertainty (e.g. confidence intervals)
☐	☒	For null hypothesis testing, the test statistic (e.g. *F*, *t*, *r*) with confidence intervals, effect sizes, degrees of freedom and *P* value noted<br>*Give P values as exact values whenever suitable.*
☒	☐	For Bayesian analysis, information on the choice of priors and Markov chain Monte Carlo settings
☒	☐	For hierarchical and complex designs, identification of the appropriate level for tests and full reporting of outcomes
☒	☐	Estimates of effect sizes (e.g. Cohen's *d*, Pearson's *r*), indicating how they were calculated

Our web collection on statistics for biologists contains articles on many of the points above.

Software and code

Policy information about availability of computer code

Data collection	Commercial software only: NAMD 3.0/QwikMD plugin, Bruker Topspin 4.2
Data analysis	Commercial software only: VMD (version 1.9.4)/Dynetan (version 2.2), CCPN 3.1, NESSY (version 1), Origin 10.2

For manuscripts utilizing custom algorithms or software that are central to the research but not yet described in published literature, software must be made available to editors and reviewers. We strongly encourage code deposition in a community repository (e.g. GitHub). See the Nature Portfolio guidelines for submitting code & software for further information.

Data

Policy information about availability of data

All manuscripts must include a data availability statement. This statement should provide the following information, where applicable:

- Accession codes, unique identifiers, or web links for publicly available datasets
- A description of any restrictions on data availability
- For clinical datasets or third party data, please ensure that the statement adheres to our policy

The data publication, to be found under https://doi.org/10.17877/RESOLV-2026-MK5LFITB, includes all experimental as well as in silico raw data. This link is provided in the data availability statement.
A source data file has been provided for line graphs, violin, and box plots in Figs. 3, 4, and 5.
Previous crystal structure data used in this study are PDB 3GCS (https://www.rcsb.org/structure/3GCS) and PDB 3HEG (https://www.rcsb.org/structure/3HEG).
Previous assignment data used here are listed under BMRB 17471 (https://bmrb.io/data_library/summary/index.php?bmrbId=17471).

Research involving human participants, their data, or biological material

Policy information about studies with human participants or human data. See also policy information about sex, gender (identity/presentation), and sexual orientation and race, ethnicity and racism.

Reporting on sex and gender	No human data
Reporting on race, ethnicity, or other socially relevant groupings	No human data
Population characteristics	No human data
Recruitment	No human data
Ethics oversight	No human data

Note that full information on the approval of the study protocol must also be provided in the manuscript.

Field-specific reporting

Please select the one below that is the best fit for your research. If you are not sure, read the appropriate sections before making your selection.

[x] Life sciences [ ] Behavioural & social sciences [ ] Ecological, evolutionary & environmental sciences

For a reference copy of the document with all sections, see nature.com/documents/nr-reporting-summary-flat.pdf

Life sciences study design

All studies must disclose on these points even when the disclosure is negative.

Sample size	NMR samples: one sample of 280 µl volume/450 µM, HTRF (Cisbio KinEASE assay): concentration of 0.2 mg/mL<br>NMR sample size is the maximum possible (detection volume), assay size (within a commercial kit) is as requested by the manufacturer.
Data exclusions	No data was excluded.
Replication	5 replicas for MD simulations, one sample/12 data points per mutant for HTRF assays, one sample per mutant for NMR
Randomization	No randomization applied. All "samples" (identical experiments) are equivalent, the order does not matter.
Blinding	No blinding is possible for these kinds of experiments, neither for NMR samples, nor for MD runs or the replicas within the HTFR experiments.<br>No group allocation or prior knowledge/differences between samples apply.

Reporting for specific materials, systems and methods

We require information from authors about some types of materials, experimental systems and methods used in many studies. Here, indicate whether each material, system or method listed is relevant to your study. If you are not sure if a list item applies to your research, read the appropriate section before selecting a response.

Materials & experimental systems

n/a	Involved in the study	
[ ]	[x]	Antibodies
[x]	[ ]	Eukaryotic cell lines
[x]	[ ]	Palaeontology and archaeology
[x]	[ ]	Animals and other organisms
[x]	[ ]	Clinical data
[x]	[ ]	Dual use research of concern
[x]	[ ]	Plants

Methods

n/a	Involved in the study	
[x]	[ ]	ChIP-seq
[x]	[ ]	Flow cytometry
[x]	[ ]	MRI-based neuroimaging

Antibodies

Antibodies used	HTRF PAb anti-phospho-ATF2 Thr69/71–Eu conjugate:<br>Polyclonal anti-phospho-ATF2 Thr69/71 antibody conjugated to europium cryptate; Cisbio/Revvity; catalogue no. 61P12KAZ. The reagent was prepared using a 1:200 working dilution and was used at a concentration of 0.666 nM in the detection solution. It was used to detect phosphorylated GST-ATF2 in the p38α HTRF kinase assay.

HTRF MAb anti-GST–d2 conjugate:
Monoclonal anti-GST antibody conjugated to d2; Cisbio/Revvity; catalogue no. 61GSTDLB. The reagent was reconstituted to 400 µg/mL, corresponding to approximately 2.67 µM, and used at an approximately 1:27 dilution to obtain a concentration of 100 nM in the detection solution. It was used to detect GST-tagged ATF2 in the p38α HTRF kinase assay.

Validation

Anti-phospho-ATF2 Thr69/71–Eu:
According to the manufacturer, the antibody recognizes ATF2 phosphorylated at Thr71 and ATF2 dually phosphorylated at Thr69/Thr71 and does not detect the corresponding non-phosphorylated ATF2 sequence. The antibody was used in a biochemical kinase-assay format.

Anti-GST–d2:
According to the manufacturer, the antibody is an IgG2a monoclonal antibody raised against glutathione S-transferase and has been validated for recognition of GST-tagged fusion proteins. It was used to detect the GST tag on the GST-ATF2 substrate.

Plants

Seed stocks

no

Novel plant genotypes

no

Authentication

no
