# Supplementary material for: Mapping of dynamic allostery within p38 alpha kinase via network analyses and NMR spectroscopy
Source: Nat Commun. 2026 Aug 22;17:10069. doi: 10.1038/s41467-026-76885-7 (PMC13594348; doi:10.1038/s41467-026-76885-7)
Supplement: Supplementary file 3 [file 41467_2026_76885_MOESM3_ESM.pdf]

nature portfolio

# Peer Review File

# Mapping of dynamic allostery within p38 alpha kinase via network analyses and NMR spectroscopy

Corresponding Author: Professor Rasmus Linser

**This file contains all reviewer reports in order by version, followed by all author rebuttals in order by version.**

Version 0:

Reviewer comments:

Reviewer #1

(Remarks to the Author)
The manuscript by Linser and coworkers presents a rigorous assessment of allostery in the p38a MKP, focused on synergy between molecular dynamics simulations and NMR. The atomic details of the allosteric pathway are nicely supported by functional assays. Overall, the work is well executed, the figures are high quality, and the manuscript is well-written. The following comments should be addressed before the manuscript can be considered further.

1)The authors picked one representative residue from each of the clusters in the allosteric network for further computational analyses. Would it be expected to change the atomic details of the computed pathways and correlations if other nodes were used instead? Does the size of each cluster, and therefore the number of possible nodes, affect the data in any way?

2)Does the simulation return more than one plausible pathway? Does the number of plausible pathways change in response to mutations?

3)The authors used Km as a quantitative proxy for substrate binding, but could this not have been directly measured via NMR? Table S7 shows that Vmax also changes in response to mutation (Vmax not reported for substrate). This makes it seem like binding (via Km) is a contributing factor but so is altered communication along the allosteric network (via Vmax or kcat). Perhaps the authors can discuss.

4)Fig. 3 is arranged seemingly out of order, where panels H and I are placed oddly. Perhaps this can be rearranged.

5)Regarding panels H and I of Fig 3, it is difficult to discern any quantitative differences in Km here. Perhaps there is a way to emphasize any change?

6)Does the V38A negative control show any change in the functional studies? It might be useful to add this into the assays just to show that allosteric mutation-induced changes are outside of normal variance.

7)Do the subtle variations in the CSP plots of Fig 4D hint at any hierarchy of importance for specific regions of the protein and its allosteric network? The allosteric mutants generally activate the same regions, showing some reciprocity in the structural impact, but the effects are not uniform.

8)The het-NOE data in Fig S18 have several values that are substantially elevated (>1.2-1.5). Can the authors comment on the reasons for this? Perhaps dipole-CSA cross-correlation, an unusually high degree of rigidity, or perdeuteration effects could be at play, or failure to reestablish Boltzmann equilibrium due to a short recycle delay.

9)Perhaps the authors can focus on site-specific changes in motion, related to data in Fig. 5? There appears to be a fairly significant overlap/similarity in the box plots for motional parameters. So, discussing the trends broadly, as in some parts of the text, may be quite difficult.

10)It may be useful to add a similar box plot for Rex to main text Fig. 5, just to round out the data and add some additional

means to separate the variants. In Fig. S19B, it actually looks like there may be a similar trend for Rex.

11)Some references appear to formatted incorrectly or incomplete. For example, Ref 26 should be Skeens E, Lisi GP "Analysis……" and Ref 60 has some extra parentheses and may be unintentionally a draft version (?). Please check.

Reviewer #2

(Remarks to the Author)
The paper by Acharyya et al. presents an interesting combination of MD simulations and spectroscopic techniques to study one of the best known dynamics systems: p38 MAP kinase.
I would not say that the results open a new frontier in the field as the dynamic connection between different parts of the protein have been known for a long time, but it provides support to previous models and open the possibility of new studies highlighting cryptic cavities. In this respect, I found interesting the discussion on the lipid pocket.
The authors ignore a very important part of the literature on p38 MAP kinase. For example, a very deep study on ative/inactive transition (Kuzmanic et al., eLife 2017, 6, 22175) and the impact of enzyme phosphorylation. From the same group a more recent paper (González et al., Nature Comm., 2003, 14, 3318) that is focuss in allosteric inhibitors, in particular mimicking the action of a natural coactivator TAB1.
The lack of attention of previous work hindered the ability of authors to perform a really deep analysis on the role of different well know elements, such as the P loop, the TAB1 binding domains, activation loop,… I would strongly recommend to review in detail previous works in the area and explain current results in light of previous findings and models.
Technical questions
- The authors fully rely on one of the many information transfer algorithm, particularly that from Schulten. It will be important to check at least one more to see how robust are the results to change in the way in which dynamic couplings are computed.
- No clear in which conditions are the NMR experiments done: phosphorylation state of the protein, apo/holo form, allosteric modulation in/out,…? Without this information, it remains challenging to understand the real meaning of the changes in the spectra shown here.
- It is also unclear which version of the protein was considered. In particular whether the protein was phosphorylated or not, and the reason for the election.
- It has been suggested the biologically-relevant formation of a disulphide bridge between Cys119 and Cys 211 (Pous et al Nature Comm, 2023, 14:7920). The same paper suggest that the absence of this bridge in experimental structure might be related to the buffer conditions. Is bridge formation consistent with unbiased simulations shown here, what would be the impact of this covalent restriction in the communication network outlined here?

Reviewer #3

(Remarks to the Author)
This manuscript by Suchandra Roy Acharyya et al. shows a comprehensive and integrative study on dynamic allostery in the p38α kinase, a significant protein in cell signalling and an important target for cancer therapy drugs.
In their study, the researchers used a multidisciplinary approach by integrating long timescale molecular dynamic simulations, dynamical network analysis by Dynetan, solution-state NMR spectroscopy, and activity assays. Through their approach, they found a long-range dynamic allosteric network between the N- and C-lobes of the kinase. The key residues are those with high betweenness centrality, including R49, D150, D168, and W207.
A significant contribution of the study is the integration of the lipid binding domain and its cryptic pocket into the dynamic allosteric network. The predictions were validated by site-directed mutagenesis, HTRF activity assays, and extensive NMR measurements. The results showed that mutations at hotspots in the network induced global changes in conformation and dynamics, leading to a decrease in activity.
The study also showed that binding with a kinase inhibitor, Sorafenib, affects the dynamic network propagation. The results indicate a new mechanism for kinase inhibition.
Overall, the study provides new insights into kinase function and is a promising approach for identifying new allosteric drug targets.

Major Comments
1. Integration and significance
The combination of approaches used in the study, which integrates computational and experimental approaches, is a strength of the work, providing strong evidence for dynamic allosteries. However, the work should be contextualized with respect to previous studies, for example, p38γ and other kinases, to clearly indicate the conceptual advance of the work.
2. Lipid pocket involvement
The incorporation of the lipid pocket domain into the allosteric network is a novelty of the work, which might be important. However, the relevance of this domain to the function of the kinase, from a pharmacological point of view, seems to be a bit speculative.
3. Experimental limitations
A limitation of the work, which might affect its validation, relates to the lack of NMR data for the W207A mutant. Although the V38A mutant serves as a good negative control, its inclusion cannot compensate for the lack of NMR data for the former.
4.. Ligand-binding conclusions
The conclusion that the binding of an inhibitor to the kinase rigidifies the structure of the kinase, which might result in a lack of allosteric communication, seems to be a bit extreme, considering the experimental validation of the results.

Minor Comments
• Clarity of figures, especially network figures, should be enhanced, as well as the labelling of figures
• Fig S19a should include x-axis label.
• Effect sizes or confidence intervals should be included with p-values
• The relationship of other kinases to p38α-specific features should be made clearer
• The authors make use of subordinate clauses, but they aren't structured correctly. For example, the sentences at lines 354-355. Other slightly off grammar abounds (eg lines 227-229, 431-434).
• Fig 4a, an indole resonance also disappears. If this is W207, it would further support their hypothesis.

Spelling
Line 73 Similaly to Similarly

Reviewer #4

(Remarks to the Author)
I co-reviewed this manuscript with one of the reviewers who provided the listed reports. This is part of the Nature Communications initiative to facilitate training in peer review and to provide appropriate recognition for Early Career Researchers who co-review manuscripts.

Version 1:

Reviewer comments:

Reviewer #1

(Remarks to the Author)
The authors have addressed each of the initial critiques with a high level of detail and careful consideration. I believe the manuscript can now be accepted for publication.

Reviewer #2

(Remarks to the Author)
I think the authors have done a very serious work to follow all my suggestions. I think the paper is acceptable for publication

Reviewer #3

(Remarks to the Author)
I want to thank the authors for revising this manuscript carefully. They answered all my questions and the manuscript is much better now. It's ready for publication.

Reviewer #4

(Remarks to the Author)
I co-reviewed this manuscript with one of the reviewers who provided the listed reports. This is part of the Nature Communications initiative to facilitate training in peer review and to provide appropriate recognition for Early Career Researchers who co-review manuscripts.

**Open Access** This Peer Review File is licensed under a Creative Commons Attribution 4.0 International License, which permits use, sharing, adaptation, distribution and reproduction in any medium or format, as long as you give appropriate credit to the original author(s) and the source, provide a link to the Creative Commons license, and indicate if changes were made.
In cases where reviewers are anonymous, credit should be given to 'Anonymous Referee' and the source.
The images or other third party material in this Peer Review File are included in the article's Creative Commons license, unless indicated otherwise in a credit line to the material. If material is not included in the article's Creative Commons license and your intended use is not permitted by statutory regulation or exceeds the permitted use, you will need to obtain permission directly from the copyright holder.
To view a copy of this license, visit https://creativecommons.org/licenses/by/4.0/

**Reviewer 1:**

The manuscript by Linser and coworkers presents a rigorous assessment of allostery in the p38a MKP, focused on synergy between molecular dynamics simulations and NMR. The atomic details of the allosteric pathway are nicely supported by functional assays. Overall, the work is well executed, the figures are high quality, and the manuscript is well-written. The following comments should be addressed before the manuscript can be considered further.

We thank the reviewer for his or her appreciation!

1) The authors picked one representative residue from each of the clusters in the allosteric network for further computational analyses. Would it be expected to change the atomic details of the computed pathways and correlations if other nodes were used instead? Does the size of each cluster, and therefore the number of possible nodes, affect the data in any way?

We would like to clarify that in dynamical network analysis, the term "node" can refer to two distinct aspects: (i) the definition of the node itself (i.e., which atom or group of atoms represents a residue), and (ii) the choice of a specific residue/node for downstream analysis. We address both points below.

Regarding node definition, in our implementation each residue is represented by a single node located at the Cα atom (as commonly adopted in residue-level dynamical network analyses). This choice constitutes a level of coarse-graining of the underlying atomic system. In principle, using a different representation (e.g., side-chain centers of mass or multiple nodes per residue) could affect the quantitative details of correlations and therefore the precise shortest paths identified. However, previous work has shown that such coarse-grained representations preserve the global topology of communication networks, including community structure and major allosteric pathways, while greatly improving computational efficiency and interpretability.
Thus, while atomic-level details may vary slightly with node definition, the dominant communication features are expected to remain robust.

Regarding the selection of representative residues for pathway analysis, residues were not chosen arbitrarily from each cluster. Instead, they were selected based on their high betweenness centrality, which identifies nodes that are part of a large fraction of shortest communication pathways anywhere in the protein and therefore act as key mediators of allosteric signaling throughout. Because betweenness centrality reflects the number of shortest paths traversing a node, residues with high centrality are expected to provide a robust description of dominant communication routes. Consequently, while choosing a different residue may alter the exact microscopic pathway, nodes with similarly high centrality would be expected to probe the same underlying communication network. In contrast, residues with low centrality would likely have a more localized effect.

Regarding the reviewer's second point, the size of a cluster does not directly bias shortest-path calculations or correlation analyses. In dynamical network analysis, clusters (or communities) represent groups of residues with highly correlated motions and reflect collective dynamical behavior. In contrast, shortest paths and betweenness centrality are computed independently on the weighted network graph (in our case using generalized correlation-based edge weights and Floyd–Warshall algorithms). Therefore, while larger

clusters contain more residues, cluster size itself does not influence the determination of shortest paths. Instead, the relevant factor is the topological role of individual nodes within the network.

Taken together, while both node definition and node selection can influence atomic-level details, the dominant features of the allosteric network are robust to these choices when using established coarse-grained representations and centrality-based selection criteria. Importantly, the dynamical network analysis framework employed here is a well-established methodology that has been extensively tested and validated over many years in the literature. (The goal of the present work was not to develop new network methods, but rather to apply these robust approaches to uncover and experimentally validate dynamic allosteric communication in p38α.) Still, these underlying thoughts were apparently insufficiently clear upon introduction of the framework to reader and have now been explained in a slightly more detailed way.

2) Does the simulation return more than one plausible pathway? Does the number of plausible pathways change in response to mutations?

In dynamical network analysis, communication between two regions is not limited to a single pathway. In general, a residue interaction network supports multiple plausible communication routes, including one optimal (shortest) path and a hierarchy of suboptimal paths that also contribute to signal propagation with different, lesser effect. This multiplicity reflects the distributed nature of allosteric communication in proteins, where information transfer typically occurs through an ensemble of partially redundant pathways rather than a single unique route.

Generally, the absolute number of contributing pathways is difficult to count as more and more pathways could be considered that, conversely, provide a lesser and lesser relative contribution to the communication. In the present study, we thus deliberately focused on the dominant shortest pathway identified in the wild-type structure as a well-defined and reproducible reference. This choice allows for a consistent comparison across systems and provides a sensitive probe of how mutations affect the most efficient communication route present in the native network. We tried to clarify this in the updated version of the manuscript.

To address the reviewer's second point, our results indicate that mutations do not simply weaken this pathway, but instead lead to a reorganization of the communication network. Specifically, we observe a pronounced decrease in correlation along the wild-type shortest path upon mutation. At the same time, the underlying network connectivity is altered such that the original wild-type pathway is no longer the dominant route in the mutant systems (see figure). This indicates that mutations can both redistribute communication across alternative pathways and potentially change the relative importance of these routes, even if the full ensemble of suboptimal paths was not explicitly enumerated in this analysis.

Importantly, across the five independent replicas for each system, the wild-type optimal pathway was highly reproducible (see figure), supporting that this route is a robust feature of the wild-type network rather than a replica-specific (random) artifact. Therefore, our conclusions are based on the consistent disruption of this dominant communication pathway and the associated redistribution of correlated motions between the two pockets. We have clarified this strategy and its implications in the revised manuscript.

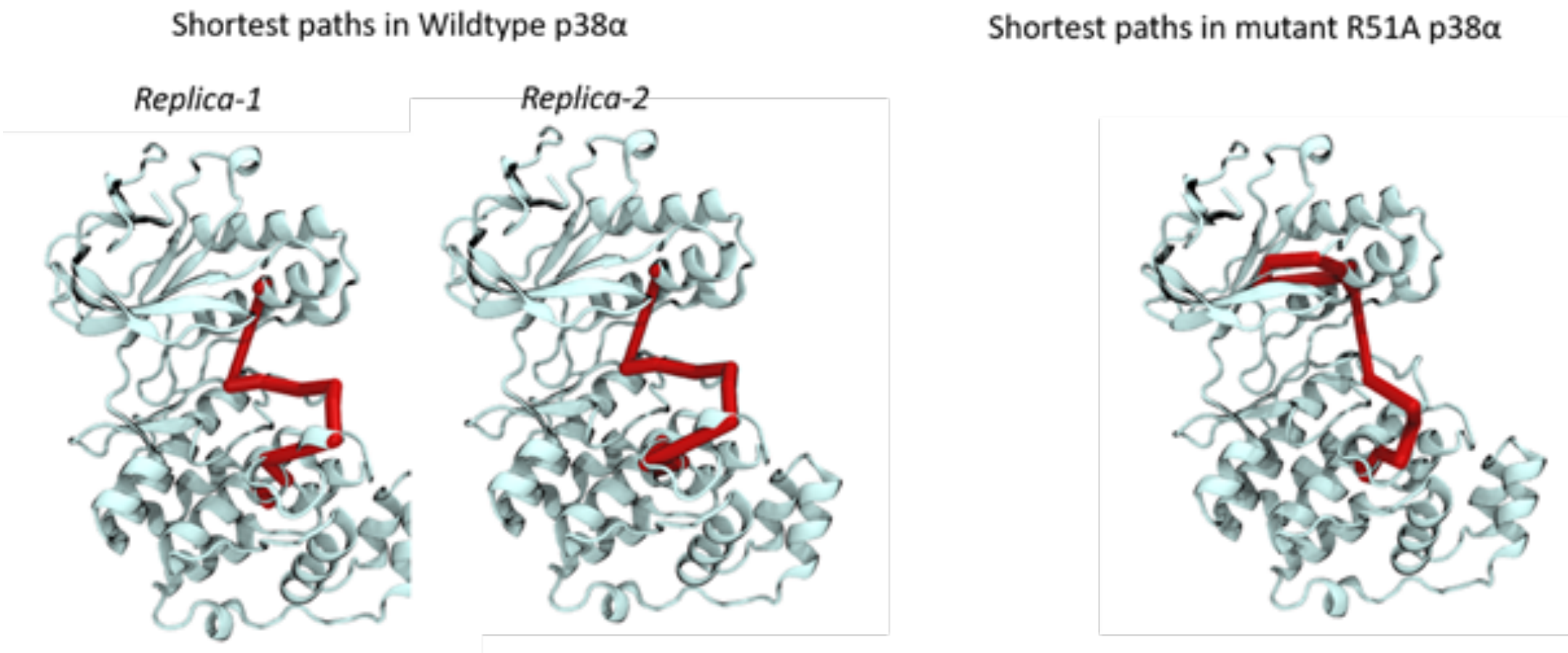


3) The authors used Km as a quantitative proxy for substrate binding, but could this not have been directly measured via NMR? Table S7 shows that Vmax also changes in response to mutation (Vmax not reported for substrate). This makes it seem like binding (via Km) is a contributing factor but so is altered communication along the allosteric network (via Vmax or kcat). Perhaps the authors can discuss.

We thank the reviewer for this valuable comment. We agree that substrate binding could, in principle, be probed more directly by NMR, which can report on protein-ligand interactions and, under appropriate (inactivated) conditions, provide binding affinities such as $K_D$. In the present study, however, we chose to use the enzymatic HTRF-based functional readout as a more integrative assay, so that the computational and NMR analyses could be related directly to a functional biochemical output.

We also agree that $K_M$ should not be interpreted as a direct measure of substrate binding affinity. In steady-state enzyme kinetics, $K_M$ is an apparent kinetic parameter and is only equivalent to the true enzyme-substrate dissociation constant $K_D$ under specific limiting assumptions. We have therefore revised the text to avoid overstating $K_M$ as a direct proxy for binding, and instead discuss it as reflecting changes in apparent substrate handling within the catalytic cycle.

The reviewer is also correct that the observed effects are not limited to $K_M$. The changes in $v_{max}$ indicate that the mutations also affect catalytic output, consistent with altered turnover and/or productive conformational transitions, rather than substrate recognition alone. We have now included the corresponding substrate $v_{max}$ values in the Supporting Information and refer to them explicitly in the revised manuscript (**Table** attached below). Taken together, the combined changes in $K_M$ and $v_{max}$ support the interpretation that these mutations perturb both the apparent substrate-dependent step and the efficiency of catalytic communication through the allosteric network.

Because our assay was designed as a comparative functional readout, and because the substrate measurements arise from a limited-point experimental format, we interpret the $v_{max}$ values primarily in a relative rather than absolute sense. In addition, the accuracy of these measurements, compared to the other values extracted, is rather weak. Nevertheless, the fact that both parameters are affected strengthens, rather than weakens, our conclusion that the mutations do not simply alter substrate binding in isolation, but more broadly disturb the allosterically coupled catalytic behavior of the kinase. We have now included the $v_{\mathrm{max}}$ values (grayed out as for the $v_{\mathrm{max}}$ values obtained for the ATP binding assay) back into the SI. This

interpretation, with due caution to imperfections experimentally, has been included into the updated manuscript.

| Protein | WT | R49A | D150A | D168A | W207A | |
|---|---|---|---|---|---|---|
| Vmax | 6.03+/-0.3 | 5.47+/-0.5 | 5.09+/-0.6 | 5.98+/-0.4 | 5.75+/-0.3 | From substrate plot |

4) Fig. 3 is arranged seemingly out of order, where panels H and I are placed oddly. Perhaps this can be rearranged.

We thank the reviewer for reflecting on this. However, we really think that this is still the best way to arrange the panels: a) is an overview, so it has to come first, the six panels b-g need to be displayed in such a 3x2 arrangement, and the biochemical data are and need to be described last in the text. Hence these panels should have the last two letters, h and i. If we rearranged, the caption would read odd, and/or the order would be different to the narrative/logics of the study. So we would leave it as is for now unless really needed.

5) Regarding panels H and I of Fig 3, it is difficult to discern any quantitative differences in Km here. Perhaps there is a way to emphasize any change?

We agree with the reviewer that the original presentation of panels H and I did not allow the differences in $K_m$ to be assessed clearly. To address this, we have revised the plots by normalizing the readout and emphasizing the transition region of the dose-response curve by plotting in a logarithmic fashion, where differences in apparent $K_m$ are most evident). In the new plots, each response was first normalized to its fitted $v_{max}$, and the concentration axis then shown logarithmically to emphasize the transition region of the dose-response curve, where shifts in apparent substrate or ATP responsiveness are most evident. This presentation makes the differences in curve position and saturation behavior visible. In the normalized representation, the visual trends are as expected from the earlier fit results. For the ATP titration, WT reaches a high fractional saturation at lower ATP concentrations than several allosteric mutants, whereas R49A and D168A show a more gradual or right-shifted response. For the substrate titration, WT also reaches saturation efficiently, while the mutant curves show altered transition behavior, with D150A and R49A appearing more right-shifted relative to WT. This revised presentation improves the visual comparison between variants and makes the shift in the fitted curves easier to appreciate quantitatively. The original plots were relegated into the SI.

6) Does the V38A negative control show any change in the functional studies? It might be useful to add this into the assays just to show that allosteric mutation-induced changes are outside of normal variance.

We thank the reviewer for this constructive suggestion. However, the PhD student that did these experiments has already left the university and would not be able to do it, such that someone else would have to get trained and execute the assays (which admittedly seems easy but is in fact associated with a wealth of preparative steps, calibrations etc. and requires specific experience). In addition, one would not be able to do the assay just for this one mutant, which

was included into the study at a later point in time, but all of the samples would have to be assessed in one go for consistency, meaning re-expressing and purification of all of the mutants again. Even though certainly nice to have, the effort (multiple weeks) was already deemed insufficiently justified by everyone when we first introduced the V38A mutant and at this point would really be a major practical problem. Note that to approximate the variance for an unperturbed case, we measured the wild type affinities in triplicates. Even though another "silent case" would certainly have extended the data further and would have been nice to have, a second benign case would not be anything that the story depends upon.
The following, site-specific NMR studies, by contrast, were designed as the actual experimental validation of the networks (with various different aspects assessed). These data are much more relevant and detailed than the rough binding affinities probed by the biochemical assay, which arguably provide rather limited and convoluted insights (also compare question 3) and mostly served as a first experimental test that encouraged us (and hopefully the reader) to dive into the NMR experiments with an atomic-level read-out.
We reformulated in such a way that it becomes clearer now that the activity assays by themselves provided very limited insights compared to the much more in-depth NMR results in focus and really are to be understood as a mere motivation for the follow-up studies.

7) Do the subtle variations in the CSP plots of Fig 4D hint at any hierarchy of importance for specific regions of the protein and its allosteric network? The allosteric mutants generally activate the same regions, showing some reciprocity in the structural impact, but the effects are not uniform.

We are grateful to the reviewer for this suggestion! To address whether the CSP profiles reveal a hierarchy of affected regions, we performed a residue-wise comparison of CSP values across the allosteric mutants R49A, D150A, and D168A using a threshold of CSP > 0.12. This analysis identified residues that were perturbed across two or three mutants, whereas others appeared only once or not at all. We have now annotated the principal high-CSP residues in Fig. 4 and Fig. S18 and summarized the shared and mutation-specific patterns in Fig. 4E and Table S9. To identify broader perturbation trends, predefined protein regions were mapped against the mutation-induced changes. Regions affected in more than one mutant were marked and consolidated across all mutants to reveal common patterns in the dynamic-network response. As the CSPs, $T_2$ relaxation, and hetNOE changes all depend on different mechanisms/time scales, we did the same analyses for all three aspects (the other two were updated into Fig. 5 and Tables S10/11.)
The analysis indicates that the allosteric mutants indeed perturb partially overlapping (but non-identical) regions of p38α. Commonly affected regions include the N-lobe/C-lobe connecting interface, the substrate-binding groove in the C-lobe, and N-lobe regulatory regions comprising P-loop-adjacent elements, and the allosteric-pocket region. These shared perturbations support reciprocal structural coupling within the allosteric network, whereas the mutation-specific CSPs suggest that individual mutations enter and redistribute the network through distinct local environments. Thus, the CSP data do suggest a hierarchy of sensitivity within the p38α allosteric network: some regions are recurrently affected across mutants, whereas others respond in a mutation-specific manner. We have revised the text and stated explicitly to clarify this point.

8) The het-NOE data in Fig S18 have several values that are substantially elevated (>1.2-1.5). Can the authors comment on the reasons for this? Perhaps dipole-CSA cross-correlation, an unusually high degree of rigidity, or perdeuteration effects could be at play, or failure to reestablish Boltzmann equilibrium due to a short recycle delay.

We thank the reviewer for pointing this out. We agree that the unusually high hetNOE values in Fig. S18, particularly those exceeding approximately 1.0, should be interpreted with caution. In our dataset, these elevated values are also associated with the largest experimental uncertainties (as witnessed by the error bars), indicating that they are unlikely to represent robust site-specific dynamical differences. Practically, such outliers can arise from a combination of experimental and analysis-related factors, including imperfect recovery of Boltzmann equilibrium for very long $^{15}$N times (the rigid residues in perdeuterated systems), sensitivity to saturation conditions, and, depending on the system and acquisition setup, contributions from cross-correlation effects rather than genuine increases in rigidity.
To prevent overinterpretation, we have revised the presentation of these data in the Supporting Information (Fig S19A). Specifically, the affected datapoints are now visually de-emphasized, and we avoid using these outliers for structural mapping across the protein. Even though the site-specific NOE data are not further interpretated and do not really play a large role for the overall picture – we now mention specifically that any discussions must exclude these poorly constrained outliers.

9) Perhaps the authors can focus on site-specific changes in motion, related to data in Fig. 5? There appears to be a fairly significant overlap/similarity in the box plots for motional parameters. So, discussing the trends broadly, as in some parts of the text, may be quite difficult.

We agree with the reviewer and thank them for suggesting a more site-specific analysis of the motional data. Because the global box plots show substantial overlap between WT and mutant distributions, we now complement this analysis with a residue-wise comparison of $\Delta R_2$ and ΔhetNOE values for each mutant relative to WT. The residues showing the largest positive or negative differences are summarized in Table S10 and a consensus representation (of residues/regions that tend to be particularly impacted, as for comment 7) mapped onto the p38α structure in Fig. 5b and d. This analysis shows that the motional changes are not uniformly distributed across the kinase, but instead cluster in defined regulatory regions. For $R_2$, recurrent changes are observed around the lipid pocket/MAP kinase insert and neighboring C-lobe regions, supporting the interpretation that the lipid pocket is dynamically coupled to the broader allosteric network. For hetNOE, recurrent changes are observed in regulatory regions including the activation-loop boundary, HRD/catalytic-loop-adjacent region, and P-loop/glycine-rich loop, indicating that allosteric-network disruption also affects fast-timescale motions in canonical kinase-control elements. In both analyses, the C-terminal docking/common docking region also shows recurrent perturbation. Since this region forms part of the MAPK docking surface used by regulatory proteins, including TAB1-associated docking interactions, these data suggest that the docking groove is dynamically linked to the allosteric network.

10) It may be useful to add a similar box plot for Rex to main text Fig. 5, just to round out the data and add some additional means to separate the variants. In Fig. S19B, it actually looks like there may be a similar trend for Rex.

We thank the reviewer for this helpful suggestion. We have now added an $R_{ex}$ box plot to Fig. 5 to complement the $R_2$ and hetNOE analyses and to provide an additional view of slower-timescale exchange contributions. The revised panel shows that the overall $R_{ex}$ distributions overlap between variants, which is in coherence with the rather small differences between the distributions discussed in the text and shown in Fig. S20b. The spread and upper-range values

do differ between mutants. In particular, R49A shows the broadest distribution and largest deviations from the WT average, followed by D150A and D168A. In contrast, V38A remains most similar to WT, consistent with its use as a negative-control mutant. However, consistent with the other data in Fig. S19, the trends for the increase of conformational exchange are not really clear/substantial. The effect of the mutations on the network hence seems to be more important on the fast timescale, which is in consistency with the *in silico* dynamic-network analyses up to around 1 μs only.

11) Some references appear to formatted incorrectly or incomplete. For example, Ref 26 should be Skeens E, Lisi GP "Analysis……" and Ref 60 has some extra parentheses and may be unintentionally a draft version (?). Please check.

We are grateful for the reviewer to spot these mistakes, which we have corrected. Ref. 60 is the citation for the data repository.

We dearly thank the reviewer for the extraordinarily committed review and highly constructive feedback.

**Reviewer 2:**

The paper by Acharyya et al. presents an interesting combination of MD simulations and spectroscopic techniques to study one of the best known dynamics systems: p38 α MAP kinase.
I would not say that the results open a new frontier in the field as the dynamic connection between different parts of the protein have been known for a long time, but it provides support to previous models and open the possibility of new studies highlighting cryptic cavities. In this respect, I found interesting the discussion on the lipid pocket.
The authors ignore a very important part of the literature on p38 α MAP kinase. For example, a very deep study on ative/inactive transition (Kuzmanic et al., eLife 2017, 6, 22175) and the impact of enzyme phosphorylation. From the same group a more recent paper (González et al., Nature Comm., 2003, 14, 3318) that is focuss in allosteric inhibitors, in particular mimicking the action of a natural coactivator TAB1.
The lack of attention of previous work hindered the ability of authors to perform a really deep analysis on the role of different well know elements, such as the P loop, the TAB1 binding domains, activation loop,… I would strongly recommend to review in detail previous works in the area and explain current results in light of previous findings and models.

We thank the reviewer for this thoughtful and constructive comment, and we appreciate the recognition that our work provides support to existing models while highlighting new aspects such as the lipid pocket.

We agree that the previous work on p38α activation, phosphorylation-dependent conformational transitions, TAB1-mediated non-canonical activation, and allosteric inhibition provides an essential framework for interpreting our results. We have therefore revised the manuscript to better incorporate and discuss this literature.
Specifically, we now cite and discuss the study by Kuzmanic et al., which showed that p38α activation is not adequately described as a simple inactive-to-active structural transition, but rather as a progressive reshaping of the conformational free-energy landscape by activation-loop phosphorylation, nucleotide binding, and substrate/docking interactions. We have added

this discussion to the Introduction to clarify the importance of dual phosphorylation at T180/Y182 in canonical p38α activation.
We have also incorporated the more recent work by González et al. on inhibitors of TAB1-induced p38α autophosphorylation. This study is now discussed in the context of regulatory surfaces outside the ATP-binding site and supports the broader concept that pathway-specific modulation of p38α can be achieved through distal or non-canonical regulatory regions. Surprisingly, a suggestion by Reviewer 1 enabled us to perform region-specific interpretation of the relaxation NMR and CSPs, collectively for the mutants that revealed C-terminal docking site as well as part of the P-loop as an affected region when the dynamic network is disrupted. And thus, in response to the reviewer's suggestion, we have further revised the Results and Discussion to interpret our findings explicitly in relation to established p38α regulatory elements. These include the activation loop/DFG motif, HRD catalytic motif, hinge/interlobe interface, TAB1-associated regulatory elements, P-loop, substrate-binding groove, and lipid-binding/MAP kinase insert region.
Our findings are consistent with these earlier studies in that they support the existence of long-range dynamic coupling between regulatory regions of the kinase. We believe these revisions strengthen the manuscript by making clear how our residue-resolved dynamic network relates to previous findings on p38α activation, TAB1-mediated regulation, and allosteric inhibition. The conceptual advance of the present work is the experimental and computational mapping of a p38α communication network that links canonical regulatory motifs with the MAP kinase-specific lipid-binding domain and its associated cryptic lipid pocket.

Importantly, while prior work has characterized conformational states and regulatory mechanisms, our approach focuses on quantifying communication pathways and identifying key nodes governing information flow, including the involvement of the lipid-binding domain and its associated cryptic pocket. The identification of this pocket as an integrated component of the dynamic network provides a potential mechanistic link between distal ligand binding and functional regulation, thereby extending previous models.

Technical questions
- The authors fully rely on one of the many information transfer algorithm, particularly that from Schulten. It will be important to check at least one more to see how robust are the results to change in the way in which dynamic couplings are computed.

We agree that different network-analysis approaches may vary in how dynamic couplings are quantified, and that it is therefore important to assess whether the main conclusions are robust with respect to the choice of method. In the present study, we employed the Luthey-Schulten framework based on generalized correlation and graph-theoretical analysis, as it is a well-established and widely used approach for identifying allosteric communication pathways in biomolecular systems.

In response to the reviewer's comment, we performed an additional analysis of the wild-type trajectory using an independent method (griNN) that employs a different formalism for defining residue-residue interactions and network connectivity (see figure provided in the response). Encouragingly, the key residues identified in our study were consistently recovered: all residues highlighted in our analysis are found within the top-ranking positions (top 20 out of ~360 residues) in the griNN-based betweenness centrality ranking. In addition, residues such as K66 and R136, which emerged as important nodes in our analysis, were likewise identified among

the highest-ranking positions in the independent approach. This level of agreement supports that the principal findings are not an artifact of a single computational framework.

At the same time, we note that exact agreement between different network-analysis methods is not expected. Variations in node definition, edge weighting, and correlation metrics can lead to differences in the detailed topology of the network and in the specific pathways identified. However, the recovery of the same high-centrality residues across distinct methodologies indicates that the key features of the communication network are robust, even if fine-grained details may differ.

Importantly, we emphasize that our conclusions are not based solely on computational analysis, but are further supported by experimental validation through NMR and biochemical assays. This combined approach provides an additional level of robustness that mitigates potential method-dependent biases inherent to any single computational framework.

As this additional analysis was performed specifically to address the reviewer's concern, we provide it here for reference rather than incorporating it into the main manuscript, as a full benchmarking of different network approaches would be beyond the scope of the present study. We have, however, added a statement in the revised manuscript acknowledging the existence of alternative methodologies and the importance of complementary experimental validation. Also, we stressed again that the *exact* details of the network depend on a variety of factors and are unlikely to be obtained accurately in any simulation, such that the findings of this study, as well as in many other dynamic-network studies need to be understood with respect to the existence or absence of communication from/to different parts of the protein in a qualitative way, rather than being able to nail down protein physiology in absolute detail.

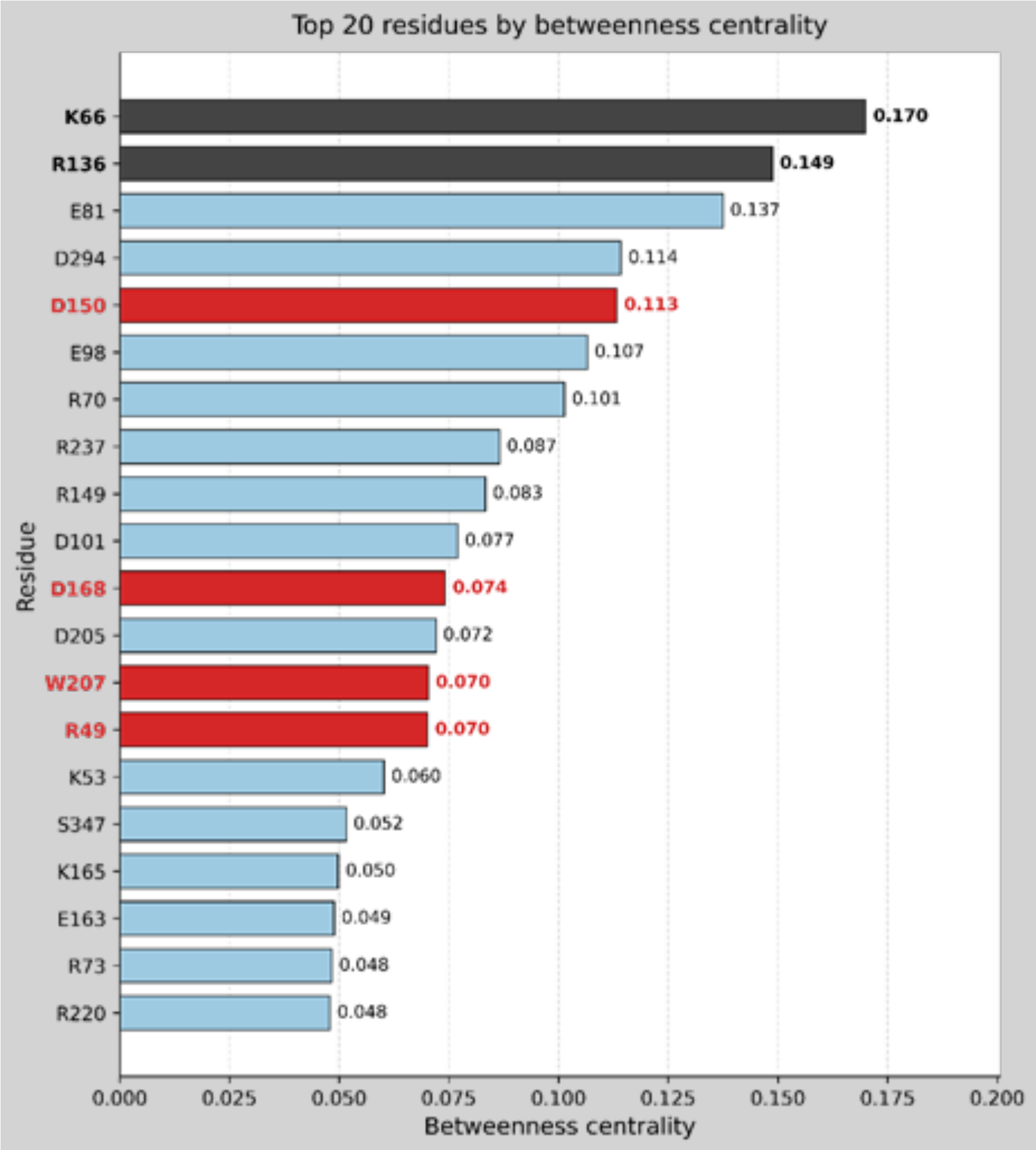


- No clear in which conditions are the NMR experiments done: phosphorylation state of the protein, apo/holo form, allosteric modulation in/out,…? Without this information, it remains challenging to understand the real meaning of the changes in the spectra shown here.

All NMR experiments were performed on the apo, unphosphorylated form of p38α, in the absence of any bound ligands or allosteric modulators. We have clarified these experimental conditions explicitly in the revised manuscript.

In addition, we note that the molecular dynamics simulations used for comparison were also performed on the apo system, ensuring consistency between the computational and experimental analyses.

We would like to emphasize that, under these conditions, the protein should not be interpreted as occupying a single rigid conformational state. Rather, the NMR observables reflect a dynamic conformational ensemble in solution. Based on available structural and biochemical data, the DFG-in conformation is expected to be the predominant state in the absence of phosphorylation and ligand binding. However, the observed chemical shift perturbations and relaxation properties report on changes in the entire underlying ensemble and its dynamics, rather than transitions between well-defined static structures.

We have added clarifications in the manuscript to better guide the interpretation of the NMR data in this context.

- It is also unclear which version of the protein was considered. In particular whether the protein was phosphorylated or not, and the reason for the election.

The protein studied in this work corresponds to the apo, unphosphorylated form of p38α, and this has now been clarified explicitly in the revised manuscript.

This choice was motivated by both practical and conceptual considerations. From an experimental standpoint, obtaining sufficiently high quantities of triple-labeled, phosphorylated and/or ligand-bound kinase suitable for backbone-detected NMR is highly challenging. As a result, the apo, unphosphorylated state represents the most accessible and well-controlled system for detailed NMR characterization.

Importantly, this same state was used consistently in both the NMR experiments and the MD simulations, ensuring direct comparability between computational and experimental results.

From a conceptual perspective, our goal was to characterize the intrinsic dynamic communication network of the kinase architecture, independent of specific activation or binding events. While phosphorylation and ligand binding are known to modulate the conformational ensemble and potentially reshape communication pathways, the qualitative existence or absence of long-range dynamic coupling between functional regions can be considered fundamental properties of kinase structures. In this sense, the apo, unphosphorylated form does provide a well-defined baseline from which such intrinsic communication features can be identified, even though in the life-cycle of the kinase multiple chemical states exist, each of which could be functionally/beneficially affected by the communication pathways as such or negatively affected by, e.g., binding of suitable allosteric effectors into the lipid pocket.

We have added a discussion in the revised manuscript to clarify this point and to outline how such effects could be explored in future work.

- It has been suggested the biologically-relevant formation of a disulphide bridge between Cys119 and Cys 211 (Pous et al Nature Comm, 2023, 14:7920). The same paper suggest that the absence of this bridge in experimental structure might be related to the buffer conditions. Is bridge formation consistent with unbiased simulations shown here, what would be the impact of this covalent restriction in the communication network outlined here?

We thank the reviewer for making us aware of the disulfide bridge between Cys119 and Cys162 reported to potentially be formed under specific cellular conditions. (We think this is what the reviewer refers to, it is what the mentioned paper describes. Cys211, on the other hand, exists but is too far away.) This putative disulfide bridge would sit at the very back/on the left side of the protein (referring to the conventional orientation in depiction of kinases) outside the network, whereas the routes of communication between the N- and the C-lobe are found on the front right (compare manuscript figure 2b). Hence, whether the two adjacent cysteines are covalently connected or not would very likely not disrupt the pathways and interdependencies between the lobes. An additional argument against a major impact on the fundamental findings of our study is that a covalent bond (within an overall maintained protein architecture) would rather lead to an increase of motional correlations instead of

putting the revelations of existing allosteric communication at risk. However, while a fundamental disruption of the basal communication network and a substantial bearing the associated conformational ensemble of p38α even under those cellular conditions is unlikely, a potential modulation of the network cannot be ruled and has now been mentioned as a possibility to further tune the characteristics of the kinase along its life cycle.

We generally thank the reviewer for his/her highly reflective and constructive feedback, hence helping us to improve the manuscript in the various ways mentioned above.

**Reviewer 3:**

This manuscript by Suchandra Roy Acharyya et al. shows a comprehensive and integrative study on dynamic allostery in the p38α kinase, a significant protein in cell signalling and an important target for cancer therapy drugs.
In their study, the researchers used a multidisciplinary approach by integrating long timescale molecular dynamic simulations, dynamical network analysis by Dynetan, solution-state NMR spectroscopy, and activity assays. Through their approach, they found a long-range dynamic allosteric network between the N- and C-lobes of the kinase. The key residues are those with high betweenness centrality, including R49, D150, D168, and W207.
A significant contribution of the study is the integration of the lipid binding domain and its cryptic pocket into the dynamic allosteric network. The predictions were validated by site-directed mutagenesis, HTRF activity assays, and extensive NMR measurements. The results showed that mutations at hotspots in the network induced global changes in conformation and dynamics, leading to a decrease in activity.
The study also showed that binding with a kinase inhibitor, Sorafenib, affects the dynamic network propagation. The results indicate a new mechanism for kinase inhibition.
Overall, the study provides new insights into kinase function and is a promising approach for identifying new allosteric drug targets.

We dearly thank the reviewer for his or her appreciation!

Major Comments
1. Integration and significance
The combination of approaches used in the study, which integrates computational and experimental approaches, is a strength of the work, providing strong evidence for dynamic allosteries. However, the work should be contextualized with respect to previous studies, for example, p38γ and other kinases, to clearly indicate the conceptual advance of the work.

We have more clearly pointed out and specifically compared our results on this protein with past work on other proteins. In the Results, we already relate the p38α community structure to experimentally derived dynamic clusters in p38γ and to previously described community architectures in PKA. We also compare specific network hotspots and communication pathways with regulatory elements identified in p38γ, PKA, Abl, EGFR, and related kinases, including the DFG motif, HRD catalytic motif, hinge region, regulatory spines, substrate-binding groove, and lipid-binding/MAP kinase insert region. These comparisons show that our p38α network is consistent with established principles of kinase dynamic allostery while also revealing p38α-specific features, particularly the integration of the lipid-binding domain and cryptic lipid pocket into the communication network (Lines 141-207). In particular, as also mentioned by Reviewer 2 above, part of the existing literature had previously been missing in this comparison, and we were grateful to be made aware (lines 47-51). In addition,

we now mention again the technical differences of our study with respect to the various examples of the related literature (lines 80-106) in the final sentences of the introduction (lines 132-136). As already mentioned by the reviewer, apart from applying network analysis to a kinase target that had not been specifically interrogated by this means before, our study represents an integrated assessment based on various methods and specifically focuses on the potential allosteric addressability of the target via the MAPK insert and its associated lipid pocket. We have updated all of these aspects into the revised version of the manuscript.
To address the reviewer's point more directly, we have now updated the Discussion section, emphasizing that the conceptual advance of the present work is not simply the observation of dynamic allostery in p38α. Rather, our study provides a residue-resolved and experimentally supported dynamic-network model that links canonical kinase regulatory motifs with the MAP kinase-specific lipid-binding domain and its associated cryptic lipid pocket. This revision clarifies how our combined computational and experimental approach extends previous work on p38γ and other kinases by identifying specific communication routes through which the lipid pocket is dynamically integrated with established regulatory elements of p38α.

2. Lipid pocket involvement
The incorporation of the lipid pocket domain into the allosteric network is a novelty of the work, which might be important. However, the relevance of this domain to the function of the kinase, from a pharmacological point of view, seems to be a bit speculative.

We thank the reviewer for this thoughtful comment. We agree that, in the current form, the possible pharmacological relevance of the lipid pocket was stated too strongly and could appear speculative. We have therefore revised the text, hinting more specifically to the persistent conundrum in the field whether or not the lipid pocket deserves more stringent investigation or not, and have stated very clearly that our results do not confirm the pocket as a validated target. Indeed, owing to insufficiently strong binders, a pharmacological role, harnessing (long-sought) allosteric effectors of MAP kinases, has *not* been established. The fact that this region now emerges in our studies as an allosterically coupled region within the communication network of p38α may hence serve as a new motivation for further medical-chemistry work. This interpretation, cautiously formulated, is nicely consistent with recent, unexplained experimental observations that ligands engaging this region can influence kinase dynamics. We have tried to rephrase these questions and thoughts in a more precise way.

3. Experimental limitations
A limitation of the work, which might affect its validation, relates to the lack of NMR data for the W207A mutant. Although the V38A mutant serves as a good negative control, its inclusion cannot compensate for the lack of NMR data for the former.

We agree with the reviewer that the presence of NMR data for the W207A mutant would have strengthened the experimental validation of the computational analysis further. We have noted in the manuscript more specifically that an eventual achieving of isotope-labeled expression of W207A in sufficient quantities for NMR measurements after the multiple failed attempts would have increased the completeness and consistency of the NMR data with the initial computation. On the bright side and as now explained more clearly, we were, however, able to produce limited amounts of the corresponding *unlabeled* protein, which allowed us to assess this mutant in the HTRF assay and confirm that it remains functionally perturbed at the biochemical level, albeit without the structural resolution provided for the other mutants by NMR. Most importantly, however, *all* of the various network mutations would have served/do serve the same purpose of qualitatively validating the general finding of allosteric communication

between the lobes, including the MAP kinase insert. Indeed, we had only started to generously include biochemical and NMR characterization of *four* network mutants (a vast amount of work and resources experimentally) to be "on the safe side", i.e., already taking biochemical pitfalls, which can never be ruled out up front, into account. Hence, even with the eventual three experimental validations and one negative control added later only, the computational findings can faithfully be assumed to be correct.
By contrast, V38A indeed serves as a *negative control* in the broader context of the study, rather than being a "compensation" for the lack of NMR characterization of W207A. (Indeed, these are two different aspects, and the wording may have been misleading.) In addition to more specifically explaining the aim of broadly validating the computational work by a number of network mutations in general, opposed to verifying individual nodes, we have revised the wording on the different rationale behind the V38A mutant to avoid any misunderstanding on the readers' side.

4. Ligand-binding conclusions
The conclusion that the binding of an inhibitor to the kinase rigidifies the structure of the kinase, which might result in a lack of allosteric communication, seems to be a bit extreme, considering the experimental validation of the results.

We thank the reviewer for raising this important concern. We agree that the original wording was too strong. In particular, describing inhibitor binding as causing "rigidification" of the kinase is an oversimplification and is not directly established by the experimental data presented here.
Our data support a more limited conclusion, namely that ligand binding is associated with a reduction in the correlations observed along the principal communication pathways between different regions of the kinase. However, the underlying physical basis of this effect cannot be assigned unambiguously from the present study alone. It may reflect altered conformational dynamics, redistribution of the ensemble, dampening of specific coupled motions, or other changes in network communication, rather than rigidification in a strict sense.
We have therefore revised the text to remove this overinterpretation and now describe the effect more cautiously as a ligand-dependent attenuation of allosteric communication, without assigning a specific mechanistic origin beyond what is directly supported by the data.

Minor Comments
• Clarity of figures, especially network figures, should be enhanced, as well as the labelling of figures

We thank the reviewer for suggesting the changes. We have revisited all figures to improve clarity and labelling.

• Fig S19a should include x-axis label.

We thank the reviewer for pointing out the mistake. FigS19A (now Fig. S20A) now has an x-axis label.

• Effect sizes or confidence intervals should be included with p-values

Confidence intervals and other statistical parameters are now included in detail. This is the new Table S6.

• The relationship of other kinases to p38α-specific features should be made clearer

We have analysed our results with previous findings and other kinases in much more detail and we have made it clearer in the main text.

• The authors make use of subordinate clauses, but they aren't structured correctly. For example, the sentences at lines 354-355. Other slightly off grammar abounds (eg lines 227-229, 431-434).

We thank the reviewer for noticing the mistakes. Even though we are not entirely sure about the line numbering in the pdf provided to the reviewers, the reviewer probably refers to the usage of a comma after "both", which has been eradicated where previously used incorrectly. We have tried to broadly improve the sentence constructions and grammar corrections in the main text.

• Fig 4a, an indole resonance also disappears. If this is W207, it would further support their hypothesis.

We thank the reviewer for pointing this out. We agree that assignment of the disappearing indole-like resonance would be valuable, especially if it corresponded to W207. We searched the available p38α assignment data, including the extensive work by Shimada and co-workers, but did not find an assignment for this resonance. Therefore, although the disappearance of this peak suggests perturbation of a tryptophan indole environment, we cannot confidently assign it to W207 and have not used it as direct evidence for W207-specific effects.

Spelling
Line 73 Similaly to Similarly

We have corrected the spelling mistake.

We are very grateful to the reviewer, his/her commitment, and the very constructive feedback. The above queries have been extremely helpful to eradicate various flaws and strengthen the manuscript in various ways.

Reviewer #4 (Remarks to the Author):

I co-reviewed this manuscript with one of the reviewers who provided the listed reports. This is part of the Nature Communications initiative to facilitate training in peer review and to provide appropriate recognition for Early Career Researchers who co-review manuscripts.

We also thank this reviewer for the time and reflections on the manuscript!
